# Estimating Mass Properties of Dinosaurs Using Laser Imaging and 3D Computer Modelling

**DOI:** 10.1371/journal.pone.0004532

**Published:** 2009-02-19

**Authors:** Karl T. Bates, Phillip L. Manning, David Hodgetts, William I. Sellers

**Affiliations:** 1 Adaptive Organismal Biology Research Group, Faculty of Life Sciences, University of Manchester, Jackson's Mill, Manchester, United Kingdom; 2 The Manchester Museum, University of Manchester, Manchester, United Kingdom; 3 School of Earth, Atmospheric and Environmental Science, University of Manchester, Manchester, United Kingdom; Quinnipiac University, United States of America

## Abstract

Body mass reconstructions of extinct vertebrates are most robust when complete to near-complete skeletons allow the reconstruction of either physical or digital models. Digital models are most efficient in terms of time and cost, and provide the facility to infinitely modify model properties non-destructively, such that sensitivity analyses can be conducted to quantify the effect of the many unknown parameters involved in reconstructions of extinct animals. In this study we use laser scanning (LiDAR) and computer modelling methods to create a range of 3D mass models of five specimens of non-avian dinosaur; two near-complete specimens of *Tyrannosaurus rex*, the most complete specimens of *Acrocanthosaurus atokensis* and *Strutiomimum sedens*, and a near-complete skeleton of a sub-adult *Edmontosaurus annectens*. LiDAR scanning allows a full mounted skeleton to be imaged resulting in a detailed 3D model in which each bone retains its spatial position and articulation. This provides a high resolution skeletal framework around which the body cavity and internal organs such as lungs and air sacs can be reconstructed. This has allowed calculation of body segment masses, centres of mass and moments or inertia for each animal. However, any soft tissue reconstruction of an extinct taxon inevitably represents a best estimate model with an unknown level of accuracy. We have therefore conducted an extensive sensitivity analysis in which the volumes of body segments and respiratory organs were varied in an attempt to constrain the likely maximum plausible range of mass parameters for each animal. Our results provide wide ranges in actual mass and inertial values, emphasizing the high level of uncertainty inevitable in such reconstructions. However, our sensitivity analysis consistently places the centre of mass well below and in front of hip joint in each animal, regardless of the chosen combination of body and respiratory structure volumes. These results emphasize that future biomechanical assessments of extinct taxa should be preceded by a detailed investigation of the plausible range of mass properties, in which sensitivity analyses are used to identify a suite of possible values to be tested as inputs in analytical models.

## Introduction

The mass properties of dinosaurs have been the subject of on-going scientific investigation for over a century [1]–[7], reflecting not only their unique range of body forms but also the fundamental importance of mass properties as morphological, physiological and ecological traits in biological organisms. Extant vertebrate body size shows complex but discernable relationships with species geographic range size [8]–[10], abundance [11]–[12], population size [13] and latitude [14]–[15]. The pervasive inter-relationship with these and many other biotic and abiotic variables is clearly crucial to our understanding of macroevolutionary dynamics and palaeobiogeographic trends through deep time [16]–[17]. Indeed, body size has featured prominently in attempts to explain temporal and spatial trends in fossil species duration [18]–[20], directional changes within lineages [21]–[26] and survivorship patterns during mass extinction events [27]; [but see 28]. Body mass is also considered the single most important factor affecting locomotor mechanics and performance in terrestrial vertebrates [29]–[35]. Assessment of biomechanical function and performance requires full quantitative description of mass properties; in addition to body mass, the location of the centre of mass (CM) and the inertial resistance of each body segment are needed to analyze accelerations and translational movements through space [36]. Accurate quantitative predictions of mass properties are therefore fundamental to biomechanical analyses of extinct organisms and to understanding patterns of diversification and extinction in the fossil record.

Body mass reconstructions of extinct dinosaurs are most robust when complete to near-complete skeletons allow realistic physical or digital models to be produced [3]–[7]. Unique body dimensions means that indirect assessments using regression analyses to extrapolate from living forms should be cautiously applied to non-avian dinosaurs [7], [37]–[41]. However, constructing life-size physical models is clearly impractical in the case of the largest dinosaurs, while scaled modelling requires a high-level of artistic skill. It is therefore more logical to construct digital models, which are typically more efficient in terms of time and cost. The digital medium also allows the full spectrum of mass properties to be investigated with relative ease; whilst it is relatively simple to extract total body mass and CM from physical models [4] it remains extremely challenging to calculate both the mass and inertial properties of each respective body segment [see for example 42]–[43 for work on extant animals]. Furthermore, the digital environment allows incorporation of inhomogeneous density within and between body segments of a model. The importance of this feature has been demonstrated in the effects of low-density internal organs (e.g. lungs, air sacs) on mass predictions for dinosaurs [4]–[7], [44]. The ability to infinitely modify model properties non-destructively also means that sensitivity analyses can be conducted to investigate the effect of the numerous assumptions necessary in reconstructions of extinct animals. This facility is crucial given the level of subjectivity involved in constructing body and respiratory structure volumes and the choice of values for other unknown parameters such as bulk tissue density [7]. Finally, high quality visualization inherent in computational methods provides useful illustration of results and accurate comparison of mass properties between taxa.

A variety of methods have been used to digitize fossil skeletons for the purpose of mass property calculations. Henderson [6] first produced 3D body volumes from reconstructed sagittal and frontal drawings, with mass computations made by summing the mass properties of independent transverse slices through body volume. In a recent study, Hutchinson et al. [7] measured a selection of 3D landmark coordinates on the skeleton of *Tyrannosaurus rex* MOR 555 by manually gridding the museum floor beneath the mount. Skeletal landmarks were entered into a custom written Computer-Aided Design (CAD) package and combined with CT-scans of hind limb and pelvic bones to create a low resolution framework around which Hutchinson et al. [7] constructed a range of body cavities for *Tyrannosaurus*. Recently laser scanning and computer modelling technology has also been applied to create more detailed digital models of skeletons [45]–[47]. Scanning whole mounted skeletons to produce a data set in which the digitized bone retains its spatial position and articulation would be a highly effective approach to achieving fast and reliable estimates of mass and inertial properties. The scanned skeleton could be imported into a CAD package and a body outline constructed, allowing mass properties to be calculated using estimates of density for the body volume as in previous studies [6]–[7], [45], [47].

Light Detection And Range (LiDAR) imaging is a highly accurate non-invasive method of collecting 3D geometrical data that shows great promise for a variety of applications in paleobiology [48]–[50]. The facility to rapidly capture sub-centimetre surface geometry of objects from distances of up to 800 m [49]–[50] suggests the method represents an ideal tool to digitize mounted skeletons of even the largest vertebrates. In this paper we describe the digitization of five skeletal mounts of four different species of non-avian dinosaur using LiDAR imaging. The resulting digital skeletal models have been used to construct complete 3D volumetric models, which allow the mass, centres of mass and moments of inertia to be calculated for each body segment. The flexible modelling framework has also allowed us to conduct a detailed sensitivity analysis to test the effect of critical assumptions in the model reconstructions. This provides critical evaluation of the total plausible range of mass properties for these dinosaurian taxa and represents the first step towards assessing their functional anatomy using numerical biomechanical methods.

## Materials and Methods

### Study specimens

**Figure 1:**
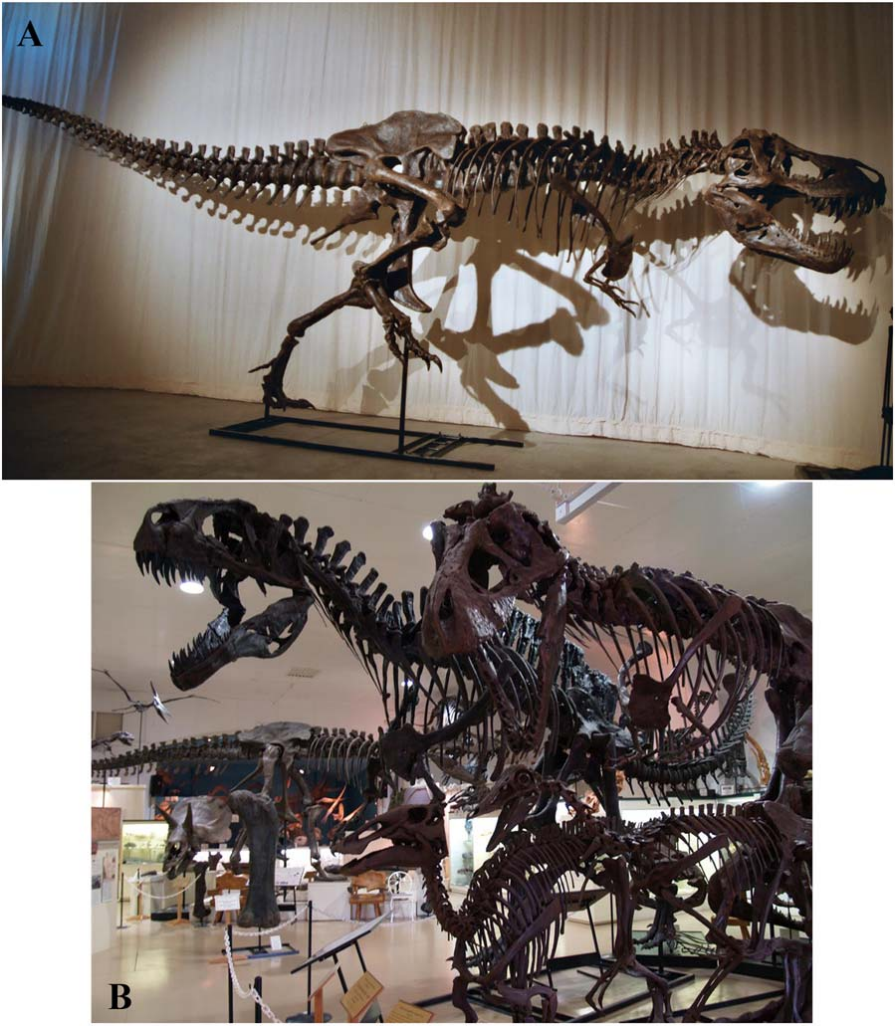
Photographs of the mounted skeletons of the five non-avian dinosaurs modelled. (A) *Tyrannosaurus rex* BHI 3033 in lateral view, and (B) *Acrocanthosaurus atokensis* NCSM 14345, *Tyrannosaurus rex* MOR 555, *Edmontosaurus annectens* BHI 126950 and *Struthiomimus sedens* BHI 1266 (top left to bottom right).

Five specimens of four species of non-avian dinosaur (Fig. 1) were chosen because of their near-complete skeletal anatomy and significant range in body size. The two specimens of *Tyrannosaurus rex* modelled are MOR 555 (46% complete) and BHI 3033 (65% complete), the latter representing the second largest and second most complete specimen currently known. The most complete specimen (54% complete) of *Acrocanthosaurus atokensis* (NCSM 14345) was chosen to provide a different taxon of similar size to *Tyrannosaurus*. The skeleton of *Struthiomimus sedens* (BHI 1266) modelled here also represents the most complete (50%) currently known and offered a wholly different body size and form to the larger theropods. A juvenille *Edmontosaurus annectens* (BHI 126950) was the only ornithischian modelled and was the smallest and least complete (approximately 40%) specimen studied here.

### Data Acquisition

A RIEGL LMS-Z420i 3D terrestrial laser scan system was used in this study. The scanner uses a near-infrared laser that is eye safe and requires no additional safety precautions, making it ideal for scanning in museum or public galleries. The scanner is able to rapidly acquire dense 3D point data with high accuracy (maximum error of 5 mm). The unit has a range of 800 m, 80° vertical and 360° horizontal fields of view and can be powered by a 24V or 12V car battery. The scanner was operated from a laptop with an Intel Core 1.83 GHz. CPU, two gigabytes of RAM, and Microsoft Windows XP. The software package RiSCAN PRO enables an operator to acquire, view and process 3D data as it is acquired, increasing the level of quality control on scan data [50]. Measurements of the lengths of proximal limb bones (femur and fibula) taken from raw scan data matched those measured manually using a tape measure.

**Figure 2:**
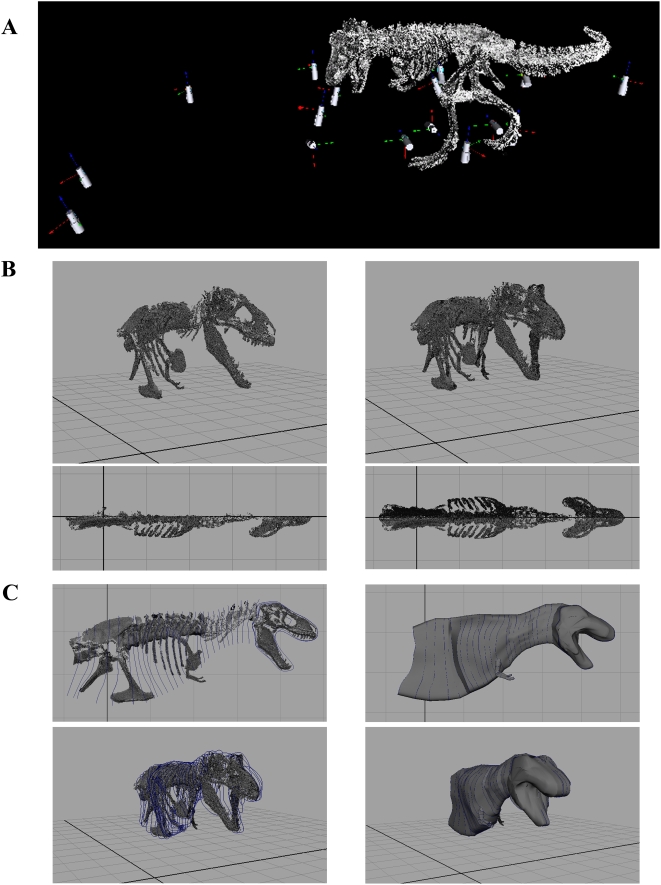
LiDAR data collection and processing. (A) The mounted skeletons were scanned from a variety of perspectives to provide full 3D coverage and eliminate ‘shadows’ in the data set. (B) The segmented right-hand side of the skeleton was aligned with Maya's x axis and mirrored to produce complete symmetrical models (*T. rex* MOR 555 in oblique right craniolateral and dorsal views). (C) Body outlines were constructed using Non-Uniform Rational B-Spline (NURBs) circles, with a single NURBs used to define the body outline around each vertebrae in the body segments (neck, thorax, sacrum and tail). Closed body cavities surfaces were then generated by ‘lofting’ a continuous surface through consecutive NURBS circles to produce discrete body volumes for each segment (*T. rex* MOR 555 in right lateral and oblique right craniolateral views).

Scan resolution describes the number of X, Y, and Z points per unit area in the scan (i.e. the density of points within the resulting 3D point cloud). High-resolution scans are characterised by a small spacing between scan points, producing high density 3D point clouds. Previous palaeontological applications of LiDAR have shown the REIGL LMS-Z420i is capable of sub-centimetre modelling of object geometry from a variety of ranges [49]–[50]. Multiple scan stations were used to capture the full 3D geometry of the mounted skeletons (Fig. 2a). At each scan station a standard 360 degree panorama scan (1998000 scan points) was performed to acquire a single scan of the entire museum gallery. Viewed on the laptop, panorama scans were then used as templates to select an area (i.e. the mounted skeleton) for higher resolution scanning. At least one higher resolution scan (0.008–0.01 m point spacing) of each mounted skeleton was acquired from each scan station. Digitizing all five mounted skeletons using this approach was extremely rapid and took just one day of scanning.

### Processing scan data

It is first necessary to align scan data collected from each discrete scan station in order to merge the point clouds into a single 3D model [50]. The LiDAR panorama scans from each scan station were imported into the PolyWorks software package (www.innovmetric.com) and merged to create the alignment matrices for each individual scan station. The ‘n-point pair alignment’ function was used to manually pick three or more points that were easily identifiable in two overlapping scans. The point clouds were then automatically aligned using an automatic ‘Best-fit function’ tool that uses a least squares algorithm to give a statistical best-fit between two scans [50]–[51]. This process is repeated until all panorama scans form a merged network of point clouds, aligned to extremely high precision (standard deviation of less than 10^−7^ in a project's coordinate system).

Having aligned the data set, RiSCAN PRO was used to simultaneously merge and filter overlapping scans. A merged model of each mounted skeleton was produced using all points from the panaroma and higher-resolution scans, with unwanted points (e.g. gallery walls and floor) manually deleted. Each skeleton was then divided into discrete body segments to allow their individual mass properties to be calculated. An octree filter was applied to most segments of the models to reduce the number of points and increase manageability of the data set with minimal cost to resolution. The octree filter divides the total area of the scans into cubes with specified edge lengths and calculates a singe representative point for each cube. The point clouds representing each skeletal segment were then triangulated in RiSCAN PRO. The resulting triangulated mesh can then be decimated in areas of low topographic variation to reduce the number of triangles in the mesh without affecting the gross geometry. This again greatly improved the manageability of the data set, particularly in the cases of the larger skeletons modelled.

### Constructing body segment outlines

The CAD package Maya (www.autodesk.com/maya) was used to construct body outlines around the digital skeletal models. The triangulated mesh of each skeletal segment from the right side of each dinosaur were imported into Maya individually, retaining their original spatial coordinates. This allowed the long axis or mid-line of each skeleton to be aligned with the x axis in *Maya* without disarticulation and the need to digitally remount each segment. Each right-hand segment was then copied and mirrored to produce the left sides of the skeletons and complete bilaterally symmetrical skeletal models (Fig 2b). An effort was made to minimize re-articulation of skeletons in order to retain comparability with the physical mounts; only the limb segments of *Acrocanthosaurus*, *Struthiomimus* and *Edmontosaurus* were re-articulated to improve the ease of the volumetric reconstructions.

Body outlines were constructed using Non-Uniform Rational B-Spline (NURBs) circles, whose geometry was defined by 30 landmark points (Fig. 2c). NURBs represent a highly flexible shape modelling format and can be used to generate standard geometries (such as parabolic curves, circles, and ellipses) in addition to complex free-form curves. Body outlines could therefore be constructed without geometrical restriction and the choice of thirty landmarks points to define NURBs circles was more than sufficient for the complexity desired. For the body segments (neck, thorax, sacrum and tail) a single NURBs was used to define the body outline around each vertebrae (Fig. 2d). For limb and skull segments the number of NURBs circles varied according to the complexity required to model the segment outline in its respective articulation. Closed body cavities surfaces were then generated by ‘lofting’ a continuous surface through consecutive NURBS circles to produce discrete body volumes for each segment (Fig. 2d).

### Modelling lungs and air sacs

The CAD environment allows easy incorporation of objects within reconstructed body volumes. This enabled us to reconstruct the size and shape of embedded respiratory structures on the basis of osteological and phylogenetic inferences of anatomy [52]–[54], without being restricted to simplified geometric shapes.

**Figure 3:**
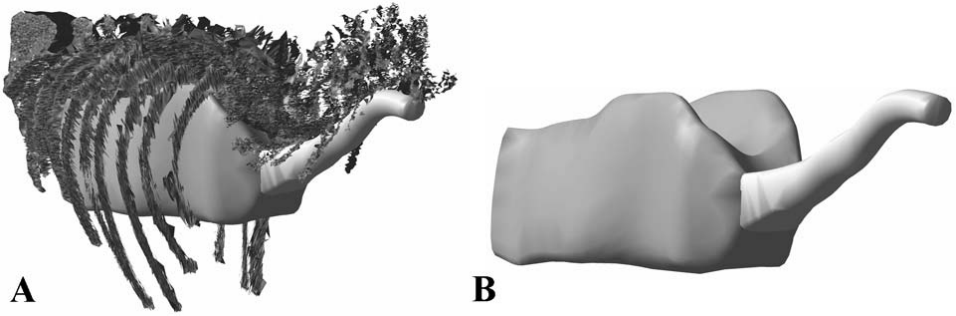
Best estimate reconstructions of thoracic and pharyngeal air sacs in *Tyrannosaurus rex* MOR 555, shown in oblique right craniolateral views.

Respiratory structures were originally created as simple NURBs cylinders and subsequently re-modelled or ‘deformed’ into the required shapes (Fig. 3). The thoraxic segments of the theropod models included a single dorsal cavity to represent lungs and their associated air sacs (Fig. 3). These bodies were shaped so that they filled the cavity between the centra of the dorsal vertebrae and the ribs, following reconstructions based on the pneumaticity of the axial skeletons of non-avian theropods [52]–[54]. The thoracic air sac volume extended from the junction between the neck and thoracic segments (where it joined the pharyngeal air sac, see below) to just in front of the pelvis, at the border between the thoracic and sacral body volumes. The facility to zoom in to high magnifications and rotate the skeletons to any orientation allowed the desired 3D shape of the lung to be modelled with high precision. In accordance with the avian-like pulmonary anatomy favoured for non-avian theropods [53] we incorporated a pharyngeal cavity in the neck segment to mimic the trachea and oesophagus (Fig. 3). Again this cavity was shaped around the centra of the (cervical) vertebrae and ribs where present. Head segments also included small air sacs filling the antorbital and cranial sinuses, as in previous reconstructions [7].

Our initial theropod models did not include abdominal air sacs, which are currently poorly supported by phylogenetic and osteological evidence [52]–[54]. However, the effect of these structures on mass set results have been tested in the sensitivity analysis (see below). The respiratory anatomy of Ornithischian dinosaurs has received comparatively little attention and any reconstruction is likely to suffer from weaker phylogenetic support. We therefore follow the approach of previous workers in constructing a single lung cavity within the thoracic segment [6], and an additional air sac in the skull.

### Calculating mass and inertial properties

Completed models were imported into the engineering CAD pack Formz (www.formz.com) which is able to automatically calculate the volume, mass, CM and moments of inertia of any arbitrary closed shape about its principle axes based on a bulk density value input by the user. Each segment was given a density of 1000 kg m^−3^, in accordance with previous studies [3]–[4], [7].

Once the mass properties of each body segment and respiratory structures are defined in the model's coordinate system it is relatively straightforward to calculate the mass properties of the whole model. Total body mass was calculated by summing the mass of all body segments minus the mass of the air sac volume at a density of 1000 kg m^−3^ (Equation 1), such that
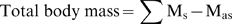
(1)where M_s_ is the mass of the segments and M_as_ is the mass of the air sac at a density of 1000 kg m^−3^. The centres of mass for the trunk or ‘HAT’ (Head-Arms-Torso), legs and whole body were calculated by multiplying the segment masses by the Cartesian coordinates of their centres of mass and dividing the sum of these by the total body mass (Equation 2), so that
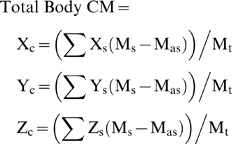
(2)where X_s_, Y_s_ and Z_s_ are the Cartesian coordinates of the segments CMs and M_t_ is the total body mass. Calculating the moments of inertia for each segment and subsequently aggregated segments is significantly more complicated, since Formz outputs the moments of inertia for each segment about its own principle axes, and its own CM. Parallel axis theorem is required to transfer these moments to the coordinate system of the aggregate body, which is located at its CM. This means calculating the distance from the CM of the aggregate body to each segment's CM and the necessary orientation change. The total moment of inertia is then given by summing the moments of inertia of each segment about the CM of the aggregate body. All calculations were performed in a custom written Mathematica script (http://www.wolfram.com), using the MechanicalSystems add-on package which contains an automated parallel axis theorem function that greatly simplifies the calculations. We calculated the moments of inertia of the head, forelimbs, thorax, sacrum and tail about their combined CM (i.e. the HAT segment CM) which then allows them to be simply summed. The moments of inertia of the hind limb segments were individually calculated about their own neutral axis, in accordance with our previous studies [35].

### Sensitivity Analysis

Although guided by skeletal morphology and phylogenetic information our initial models nevertheless constitute best estimate reconstructions with an unknown level of certainty surrounding many parameters. Soft tissue reconstructions of extinct animals inevitably contain a high-degree of subjective estimation [55], in this case in the geometry and structure of body segment volumes and airs sacs, in addition to the choice of tissue density values. To investigate the effect of our assumptions and attempt to produce a realistic range of mass set results we conducted a sensitivity analysis on each of our models. For each dinosaur we calculated the mass properties of a single slimmer and two larger models. In the slimmer models we reduced the diameter of the NURBs circles in the neck, thoraxic, sacral, tail and hind limb (thigh, shank and metatarsal) segments by 7.5%, while in the two larger models these segments were increased by 7.5% and 15% with respect to the best estimate models. This allowed us to modify our models relatively quickly and easily so that a large number of different models could be produced. However, because the NURBs circles were rarely aligned perfectly with Maya's Cartesian axes (x,y,z) it was necessary to modify their diameter in the x, y and z directions by 7.5%. This resulted in non-uniform changes in diameter (i.e. slightly less or greater than 7.5%), with the absolute value varying according to the degree of misalignment with the Cartesian axes. Whilst this is not ideal, manually altering the geometry of the NURBs circles would have been extremely time-consuming and would have likely resulted in even less standardized changes to segment volumes.

Hutchinson et al. [7] conducted a more detailed sensitivity analysis of their *Tyrannosaurus rex* mass model, in which multiple combinations of body segment volumes and air sacs were created to produce a broad range of mass set results. Whilst this approach is time consuming and may produce a suite of improbable mass set combinations it does provide important information about the possible range in combinations of segment mass properties, which may have important implications for subsequent higher-level evolutionary or biomechanical analyses [7]. For example, contention surrounding the locomotor capabilities of the largest non-avian theropods largely reflects uncertainty about the ratio of hind limb muscle mass to body mass in these animals [7], [35], [56]–[57]. To examine the effects on the overall mass set results we conducted a more detailed sensitivity analysis in the style of Hutchinson et al. [7] for each taxon, in which we experimented with a combination of trunk and leg segments from the initial sensitivity analysis. In addition to segment volumes, we also test the effects of having larger and smaller zero density respiratory structures in our thoracic and neck segments.

### Methodological validation: Extant Ostrich model

In soft tissue, functional and biomechanical studies of extinct taxa it is important that methodologies are validated using experimental data from extant species. In this case it must be emphasized that accurate volumetric modelling of a modern animal with known morphology does not increase nor decrease the ‘accuracy’ of any single prediction about the mass properties of an extinct animal with unknown soft tissue morphology. However, recent physical and digital reconstructions of extinct non-avian dinosaurs have typically been accompanied by similarly constructed models of extant taxa for the purpose of methodological validation [4], [6]–[7]. In addition to sensitivity analysis of the dinosaur models, we have constructed a volumetric model of an extant ostrich (*Struthio camelus*) using exactly the same digitization and CAD modelling procedures used for our dinosaur reconstructions. Previous workers have typically employed one of two approaches in using modern animals to validate mass predictions methods in non-avian dinosaurs. In the first approach a ‘generic’ model of an extant species is made and compared to an accepted suite of average mass properties for that particular species [4], [6]. The second, more thorough approach, involves experimentally measuring the mass properties of a dead carcass of a particular individual animal, and then comparing the predictions from a subsequent physical or digital volumetric model of that individual to the experimental values obtained directly from the specimen [7]. Our validation follows the former approach, as no mass data was available for the mounted Ostrich skeleton digitized in this study. The Ostrich skeleton used (BB.3462) is currently on display at the Manchester Museum (University of Manchester, UK). Data from the volumetric reconstruction is compared to published mass data on extant Ostriches from the literature [7], [58]

## Results

**Figure 4:**
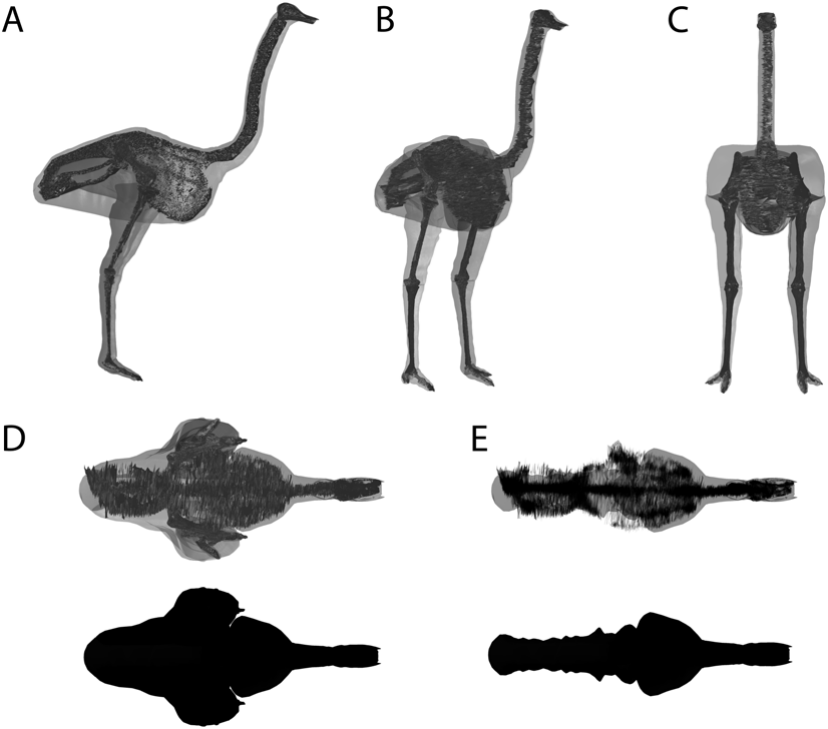
Volumetric model of an extant ostrich (*Struthio camelus*) based on a specimen (BB.3462) mounted at the Manchester Museum (UK), shown in (A) right lateral, (B) oblique right craniolateral, (C) cranial and (D–E) dorsal views (E with hind limb segments removed).

**Figure 5:**
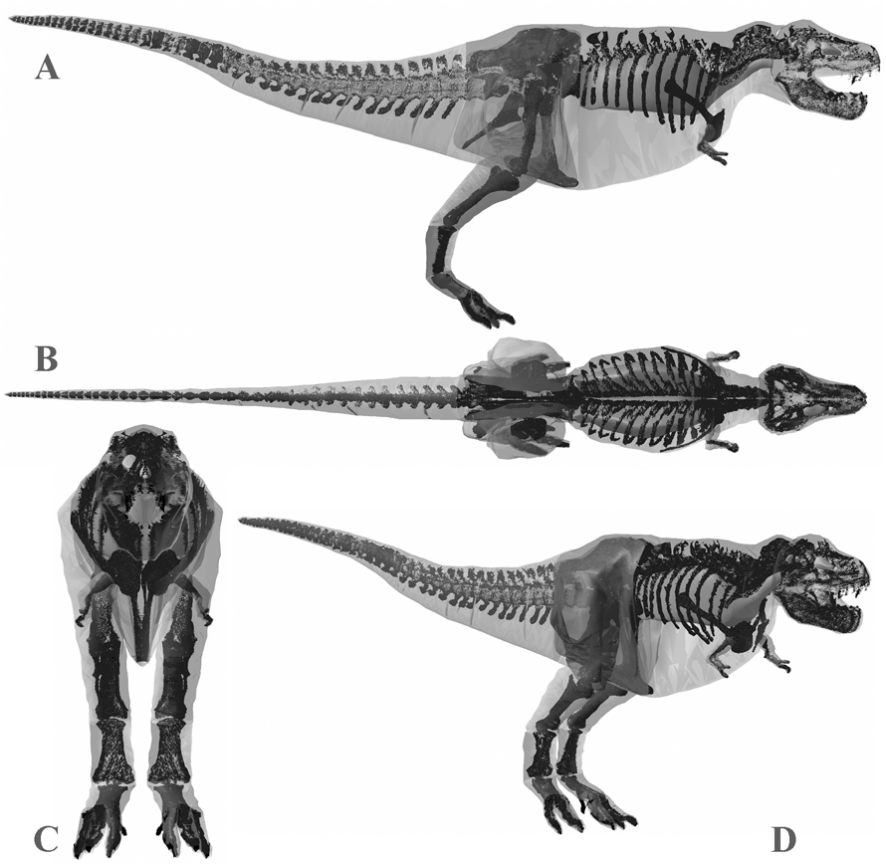
Best estimate reconstruction of *Tyrannosaurus rex* BHI 3033 in (A) right lateral, (B) dorsal, (C) cranial and (D) oblique right craniolateral views (not to scale).

**Figure 6:**
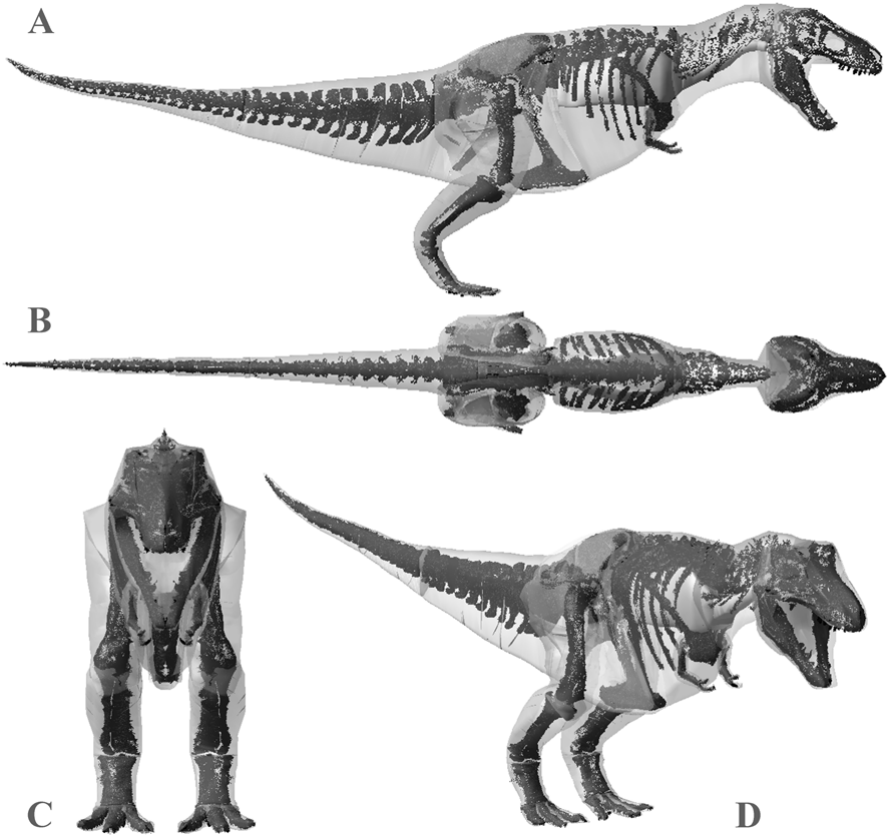
Best estimate reconstruction of *Tyrannosaurus rex* BHI MOR 555 in (A) right lateral, (B) dorsal, (C) cranial and (D) oblique right craniolateral views (not to scale).

**Figure 7:**
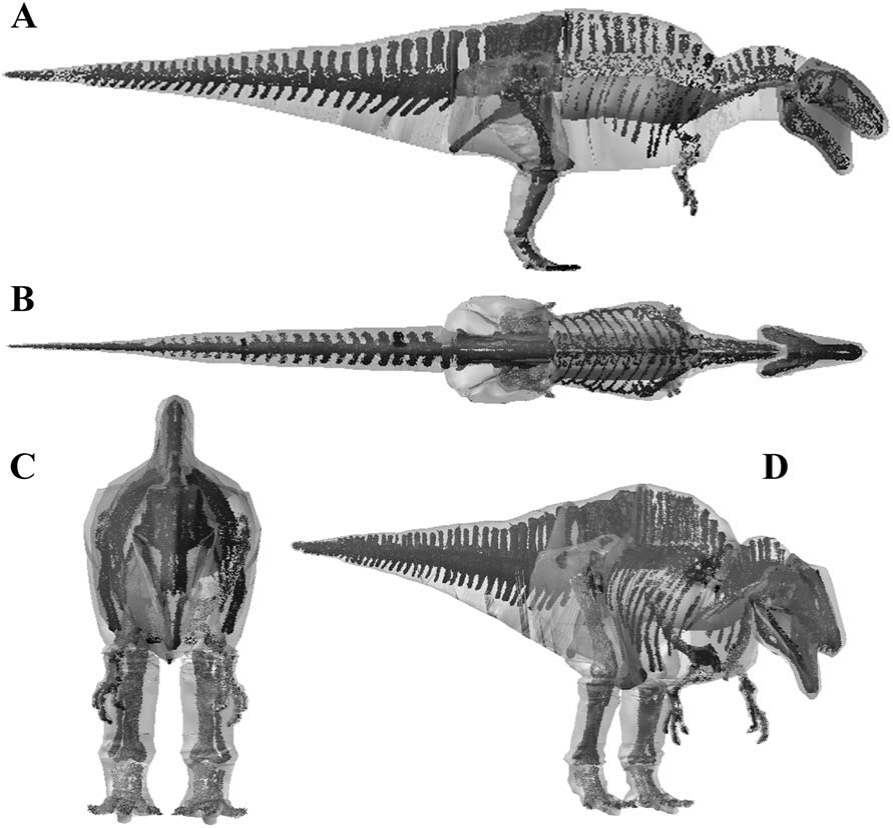
Best estimate reconstruction of *Acrocanthosaurus atokensis* NCSM 14345 in (A) right lateral, (B) dorsal, (C) cranial and (D) oblique right craniolateral views (not to scale).

**Figure 8:**
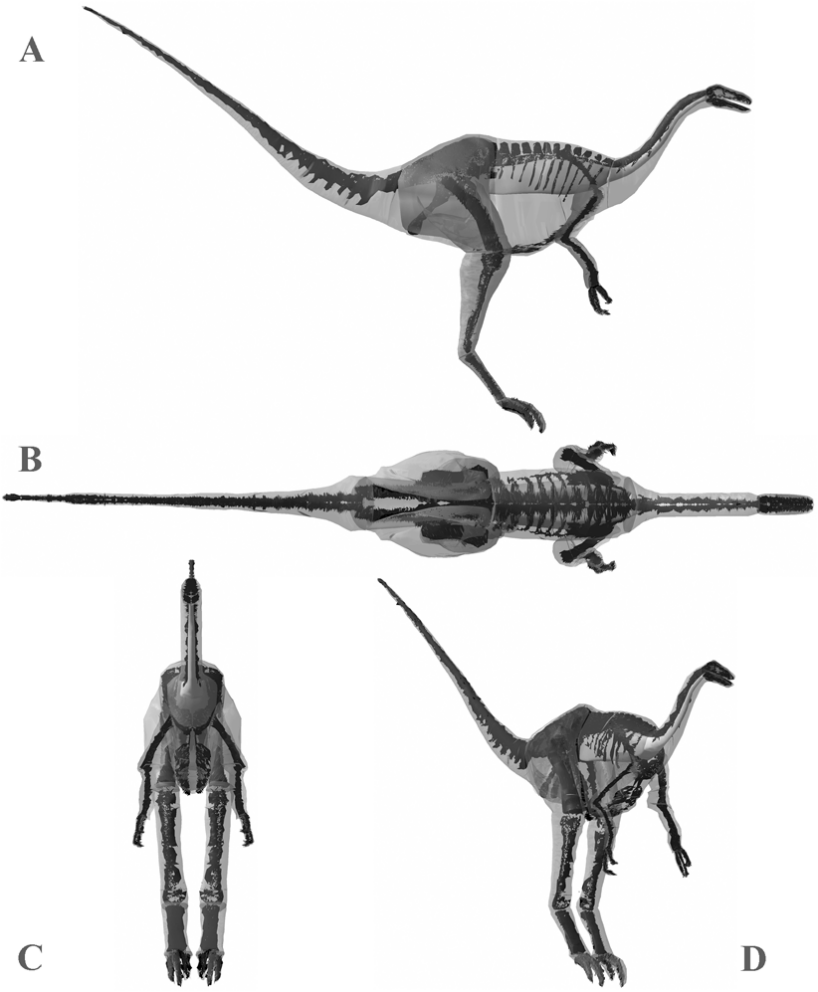
Best estimate reconstruction of *Struthiomimus sedens* BHI 1266 in (A) right lateral, (B) dorsal, (C) cranial and (D) oblique right craniolateral views (not to scale).

**Figure 9:**
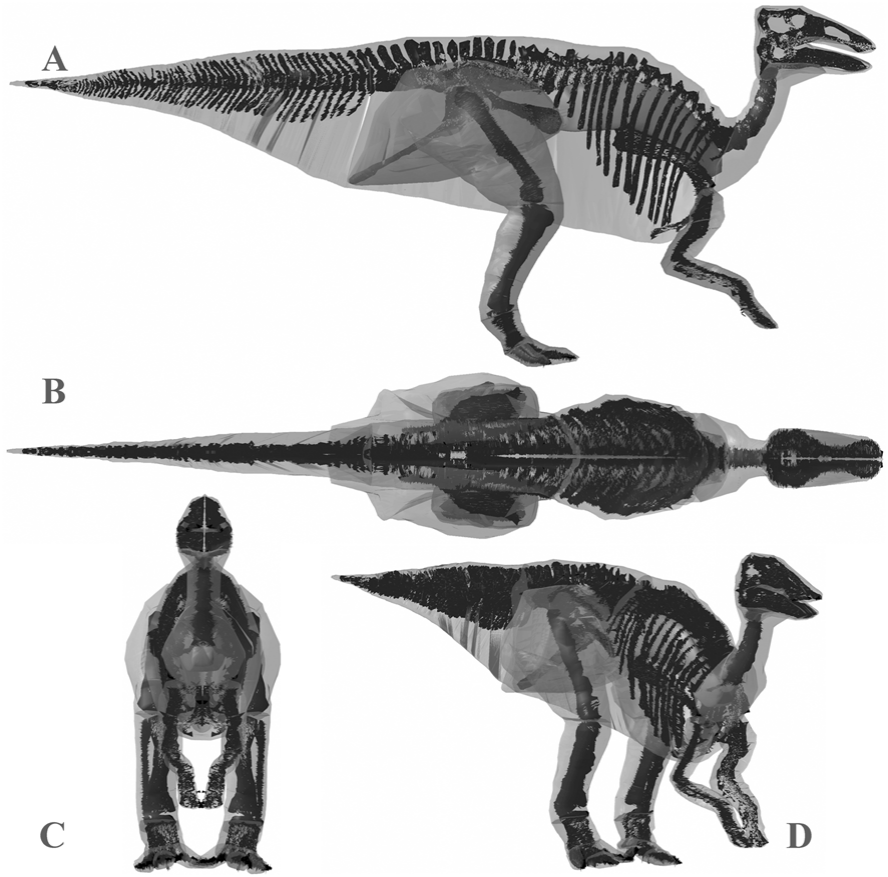
Best estimate reconstruction of *Edmontosaurus annectens* BHI 126950 in (A) right lateral, (B) dorsal, (C) cranial and (D) oblique right craniolateral views (not to scale).

The volumetric reconstruction of the extant ostrich is shown in Figure 4 and the best estimate mass models for each dinosaur are shown in Figures 5, 6, 7, 8, 9 and the calculated mass set parameters are tabulated in Tables 1, 2, 3, 4, 5, 6. The total body mass estimate for the ostrich was 72.172 kg, and the position of the torso CM was found to be 0.176 m in front and 0.114 m below the acetabulum. Total body mass estimates of the four non-avian theropods range from 423 kg for *Struthiomimus* to 7655 kg for *Tyrannosaurus rex* BHI 3033. Table 7 summarizes the mass set data for each of the initial slimmer and larger models produced in the sensitivity analysis. The largest models represented an increase of 21–29.8% in total body mass over best estimate predictions, while the smallest models were 8.2–9.9% lighter than initial predictions. The results from all subsequent sensitivity analyses, in which we experimented with different mass set combinations, are summarized in Tables 8–9. Best estimate CM positions were most significantly affected by altering the combinations of body segment volumes; the combination of large thoracic and neck segments with reduced tail segments resulted in the most craniad CM positions (4.47–8.61% body length in front of the hip joint), while enlarged tails and reduced anterior body segments brought the CM closest to the acetabulum (0.78–6.2% body length anterior to the hip joint). However, the CM remained in front and below the hip joint in all models produced. As expected, body mass and inertial values showed a positive correlation with the heaviest models consistently having the largest principal moments of inertia. The implications of these results are discussed below. The full mass set results for every model created can be found in the electronic supplementary files on-line (Supporting Information Tables S1: 1–49).

**Table 1:** Results of the volumetric model of the ostrich (BB.3462).

Segment	Net Density (kg m−3)	Volume (m3)	Mass (kg)	CM (x,y,z) (m)	Ixx Iyy Izz (kg m2)
Head-Arms-Torso (HAT)	807.423	0.052	41.986	0.103, 1.064, 0	1.508, 3.35, 4.586
Pharyngeal cavity	0	0.0004	0	-	-
Thoracic air sacs	0	0.009	0	-	-
Thigh	1000	0.006	5.965	−0.008, 1.077, −0.166	
Shank	1000	0.007	7.368	−0.004, 0.834	
Metatarsus	1000	0.002	1.723	−0.118, 0.407, −0.168	
Digit III	1000	0.0003	0.303	−0.061, 0.12, −0.184	
Digit IV	1000	0.00008	0.094	−0.103, 0.13, −0.242	
Pes	1000				
Hind limb	1000	0.015	15.137	−0.02, 0.853, 0.174	-
Whole Body	857.03	0.167	72.172	0.051, 0.975, 0	-

**Table 2:** Results for the best estimate model of *Tyrannosaurus rex* BHI 3033.

Segment	Net Density (kg m−3)	Volume (m3)	Mass (kg)	CM (x,y,z) (m)	Ixx Iyy Izz (kg m2)
Head	990.6	0.685	678.561	2.553, 3.346, 0	147.908, 5947.62, 6042.42
Air sacs	0	0.01	0	-	-
Neck	905.3	0.369	334.056	1.690, 3.376, 0	71.135, 1471.68, 1519.58
Pharynegeal cavity	0	0.035	0	-	-
Thorax	746.46	3.01	2246.83	0.383, 2.958, 0	831.162, 2220.43, 2646.88
Lungs	0	0.764	0	-	-
Sacrum	1000	1.062	1062.439	−1.319, 3.004, 0	271.522, 1159.56, 1398.81
Tail	1000	1.106	1106.037	−3.530, 3.092, 0	295.412, 12329.8, 12595.7
Arm	1000	0.011	10.931	1.138, 2.398, 0.483	7.643, 28.202, 30.566
Digit I	1000	0.001	0.644	1.315, 2.115, 0.62	0.832, 2.122, 2.458
Digit II	1000	0.001	0.548	1.33, 2.197, 0.591	0.606, 1.815, 2.037
Fore limb	1000	0.013	12.123	1.156,2.374,0.495	9.081, 32.139, 35.062
Thigh	1000	0.744	743.937	−1.323, 2.853, 0.408	183.542, 78.453, 226.284
Shank	1000	0.215	214.664	−1.887, 1.687, 0.357	13.856, 21.791, 30.382
Metatarsus	1000	0.074	73.812	−2.431, 0.914, 0.298	3.380, 1.501, 3.258
Digit II	1000	0.023	22.332	−1.897,0.11,0.29	0.257, 0.259, 0.499
Digit III	1000	0.027	26.564	−1.811,0.134,0.457	0.308, 0.540, 0.781
Digit IV	1000	0.021	20.966	−1.998,0.09,0.571	0.241, 0.242, 0.452
Pes	1000	0.07	69.863	−2.112,0.681,0.72	-
Hind limb	1000	1.102	1102.276	−1.37335, 2.19418,0.410458	-
HAT	871.1	6.257	5450.16	−0.390195, 3.06656, 0	1635.301, 23193.44, 24273.56
Whole Body	904.6	8.462	7654.71	−0.673342 2.81531 0	-

**Table 3:** Results for the best estimate model of *Tyrannosaurus rex* MOR 555.

Segment	Net Density (kg m−3)	Volume (m3)	Mass (kg)	CM (x,y,z) (m)	Ixx Iyy Izz (kg m2)
Head	984.26	0.661	650.596	3.884, 3.063, 0	235.19, 5771.57, 5946.82
Air sacs	0	0.011	0	-	-
Neck	938.15	0.471	441.871	2.783, 3.101, 0	147.379, 1645.73, 1783.41
Pharynegeal cavity	0	0.029	0	-	-
Thorax	739.52	1.721	1272.71	1.387, 2.615, 0	343.275, 553.043, 857.709.
Lungs	0	0.449	0	-	-
Sacrum	1000	0.659	659.477	0.222, 2.272, 0	283.172, 386.339, 658.997
Tail	1000	1.079	1078.774	−1.828, 2.363, 0	174.893, 9462.73, 9615.77
Arm	1000	0.009	8.845	1.89, 2.097, 0.277	3.248, 9.386, 11.244
Digit I	1000	0.0003	0.355	2.16, 1.922, 0.202	0.185, 0.578, 0.733
Digit II	1000	0.001	0.822	2.172, 1.846, 0	0.520, 1.364, 1.816
Fore limb	1000	0.01	10.022	1.923, 0.294, 0.268	3.953, 11.327, 13.794
Thigh	1000	0.689	688.552	−0.114, 2.289, 0.363	149.268, 76.017, 194.264,
Shank	1000	0.212	212.459	−0.584, 1.114, 0.426	13.684, 19.713, 27.550
Metatarsus	1000	0.044	44.408	−0.859, 0.392, 0.417	1.204, 0.906, 1.343
Digit II	1000	0.007	7.128	−0.477, 0.077, 0.25	0.038, 0.166, 0.155
Digit III	1000	0.01	9.765	−0.432, 0.09, 0.451	0.036, 0.336, 0.331
Digit IV	1000	0.009	8.814	−0.489, 0.091, 0.614	0.055, 0.227, 0.203
Pes	1000	0.026	25.707	−0.464, 0.086, 0.449	-
Hind limb	1000	0.971	971.126	−0.221, 1.761, 0.382	-
HAT	894.02	4.612	4123.23	0.901, 2.614, 0	1191.813, 17842.03, 18890.29
Whole Body	926.43	6.554	6071.82	0.541, 2.340, 0	-

**Table 4:** Results for the best estimate model of *Acrocanthosaurus atokensis* NCSM 14345.

Segment	Net Density (kg m−3)	Volume (m3)	Mass (kg)	CM (x,y,z) (m)	Ixx Iyy Izz (kg m2)
Head	981.63	0.405	397.566	3.437, 2.137, 0	45.047, 4380.4, 4411.46
Air sacs	0	0.007	0	-	-
Neck	911.07	0.336	306.118	2.325, 2.181, 0	42.1869, 1502.13, 1533.1
Pharynegeal cavity	0	0.03	0	-	-
Thorax	760.07	2.42	1839.37	1.054, 2.169, 0	546.468, 2473.5, 2674.72
Lungs	0	0.58	0	-	-
Sacrum	1000	0.768	768.158	−0.54, 2.41, 0	194.869, 474.139, 654.687
Tail	1000	1.149	1148.734	−2.77, 2.465, 0	130.905, 10988, 11088.9
Arm	1000	0.01	10.024	1.694, 1.252, −0.414	12.594, 27.204, 36.223
Digit I	1000	0.0005	0.491	1.721, 0.919, −0.585	1.076, 1.454, 2.194
Digit II	1000	0.001	1.207	1.814, 0.852, −0.531	2.804, 3.875, 5.997
Digit IV	1000	0.0006	0.639	1.815, 0.898, −0.422	1.333, 1.988, 3.092
Forelimb	1000	0.012	12.361	1.713, 1.181, 0.433	17.807, 34.521, 47.505
Thigh	1000	0.664	663.709	−0.495, 2.067, 0.35	91.336, 97.478, 159.109
Shank	1000	0.142	142.12	−0.255,0.936,0.308	11.385, 5.102, 13.239
Metatarsus	1000	0.033	32.925	−0.277,0.236,0.32	0.670, 0.633, 0.762
Digit II	1000	0.002	2.257	0.023, 0.067, 0.185	0.003, 0.004, 0.004
Digit III	1000	0.003	3.647	0.105,0.026,0.341	0.006, 0.008, 0.008
Digit IV	1000	0.002	1.767	0.019, 0.071, 0.491	0.002, 0.002, 0.003
Pes	1000	0.007	7.677	0.062, 0.049, 0.33	-
Hind limb	1000	0.847	846.524	−0.441, 1.788, 0.342	-
HAT	881.83	5.227	4484.67	0.103, 2.279, 0.	940.818, 19907.42, 20480.81
Whole Body	911.63	6.912	6177.04	−0.046, 2.144, 0	-

**Table 5:** Results for the best estimate model of *Struthiomimus sedens* BHI 1266.

Segment	Net Density (kg m−3)	Volume (m3)	Mass (kg)	CM (x,y,z) (m)	Ixx Iyy Izz (kg m2)
Head	974.01	0.0016	1.649	1.894, 2.302, 0	0.649, 4.15, 4.799
Air sacs	0	0.004	0	-	-
Neck	901.81	0.02	18.029	1.340, 1.919, 0	1.877, 19.779, 21.771
Pharynegeal cavity	0	0.002	0	-	-
Thorax	809.72	0.142	114.988	0.650, 1.659, 0	5.344, 19.320, 22.561
Lungs	0	0.027	0	-	-
Sacrum	1000	0.082	81.723	−0.019, 1.639, 0	2.839, 12.688, 14.979
Tail	1000	0.037	37.067	−0.854, 1.827, 0	3.749, 57.530, 61.112
Arm	1000	0.008	8.042	0.887, 1.384, 0.227	1.353, 3.164, 3.637
Digit I	1000	0.0001	0.157	0.887, 1.384, 0.227	0.100, 0.104, 0.169
Digit II	1000	0.0001	0.141	1.053, 0.953, 0.335	0.104, 0.087, 0.158
Digit III	1000	0.0001	0.132	0.981, 0.904, 0.326	0.049, 0.041, 0.074
Fore limb	1000	0.008	8.472	0.894, 1.36, 0.232	1.607, 3.396, 4.039
Thigh	1000	0.049	49.349	0.057, 1.604, 0.175	1.975, 1.273, 2.979
Shank	1000	0.02	19.988	0.188, 0.966, 0.15	0.864, 0.165, 0.953
Metatarsus	1000	0.004	4.446	0.221, 0.393, 0.114	0.082, 0.089, 0.066
Digit II	1000	0.001	0.656	0.444, 0.159, 0.051	0.001, 0.003, 0.004
Digit III	1000	0.001	0.807	0.51, 0.157, 0.106	0.003, 0.006, 0.009
Digit IV	1000	0.001	0.799	0.441, 0.157, 0.142	0.002, 0.004, 0.005
Pes	1000	0.003	2.262	0.466,0.158,0.103	-
Hind limb	1000	0.076	76.045	0.088, 1.196, 0.163	-
HAT	902.84	0.299	270.557	0.310, 1.677, 0	17.673, 120.261, 133.300
Whole Body	935.76	0.452	422.647	0.230, 1.504, 0	-

**Table 6:** Results for the best estimate model of *Edmontosaurus annectens* 126950.

Segment	Net Density (kg m−3)	Volume (m3)	Mass (kg)	CM (x,y,z) (m)	Ixx Iyy Izz (kg m2)
Head	962.4	0.028	27.17	1.082, 1.623, 0	5.288, 44.997, 50.065
Air sacs	0	0.006	0	-	-
Neck	1000	0.025	25.36	0.837, 1.202, 0	0.937, 27.506, 28.161
Thorax	764.97	0.264	201.952	0.240, 1.073, 0	18.363, 50.790, 61.312
Lungs	0	0.062	0	-	-
Sacrum	1000	0.177	176.939	−0.589, 1.275, 0	9.363, 42.509, 49.708
Tail	1000	0.071	71.281	−1.556, 1.36, 0	3.369, 140.812, 143.865
Fore limb	1000	0.011	11.031	0.618, 0.578, 0.129	4.734, 7.663, 11.986
Thigh	1000	0.112	112..431	−0.588, 1.184, 0.197	3.984, 6.623, 9.137
Shank	1000	0.023	22.757	−0.346, 0.575, 0.237	0.655, 0.261, 0.788
Metatarsus	1000	0.005	4.582	−0.396, 0.2, 0.203	0.023, 0.023, 0.020
Digit II	1000	0.001	1.158	−0.299, 0.084, 0.051	0.003, 0.004, 0.003
Digit III	1000	0.001	1.457	−0.296, 0.095, 0.184	0.003, 0.007, 0.007
Digit IV	1000	0.001	0.925	−0.296, 0.061, 0.281	0.002, 0.003, 0.003
Pes	1000	0.004	4.473	−0.27,0.077,0.171	-
Hind limb	1000	0.144	144.243	−0.507, 0.976, 0.203	-
HAT	893.98	0.587	524.764	−0.195, 1.194, 0	46.78793, 321.9393, 357.0826
Whole Body	929.43	0.875	813.25	−0.306, 1.116, 0	-

**Table 7:** Results of the alternative mass models of each the five modelled specimens.

Model	Net Density (kg m−3)	Volume (kg m2)	Mass (kg)	CM (x,y,z) (m)	HAT Ixx Iyy Izz
*Tyrannosaurus* BHI 3033 Plus 15%	903.18	11.006	9940.43	−0.659, 2.806, 0	2376.198, 29342.41, 30733.88
*Tyrannosaurus* BHI Plus 7.5%	893.07	9.965	8899.45	−0.704, 2.83,1 0	1869.518, 26018.87, 27149.74
*Tyrannosaurus* BHI 3033 Minus 7.5%	866.32	7.97	6904.67	−0.636, 2.830, 0	1250.244, 21001.73, 21781.5
*Tyrannosaurus* MOR 555 Plus 15%	918.3	8.384	7699.53	0.492, 2.622, 0	1759.118, 21907.18, 23358.74
*Tyrannosaurus* MOR 555 Plus 7.5%	910.36	7.641	6956.06	0.499, 2.349, 0	1448.034, 19784.4, 21020.42
*Tyrannosaurus* MOR 555 Minus 7.5%	890.76	6.264	5579.7	1.121, 2.649, 0	1071.807, 16237.88, 17195.49
*Acrocanthosaurus* NCSM 14345 Plus 15%	917.1	8.45120698	7750.61	−0.091, 2.134, 0	1647.604, 25178.05, 26119.1
*Acrocanthosaurus* NCSM 14345 Plus 7.5%	921.94	7.462207	7026.64	0.746,1 2.278, 0	1224.917, 9567.658, 10306.4
*Acrocanthosaurus* NCSM 14345 Minus 7.5%	914.21	6.092207	5569.56	−0.015, 2.146, 0	975.996, 20120.7, 20677.1
*Struthiomimus* BHI 1266 Plus 15%	946.55	0.554	524.139	0.241, 1.492, 0	24.287, 154.96, 172.847
*Struthiomimus* BHI 1266 Plus 7.5%	939.29	0.496	465.829	0.234, 1.499, 0	20.525, 136.412, 151.557
*Struthiomimus* BHI 1266 Minus 7.5%	925.83	0.412	381.444	0.239, 1.511, 0	16.615, 106.906, 118.318
*Edmontosaurus* BHI 126950 Plus 15%	941.52	1.045	983.889	−0.174, 1.088, 0	63.989, 198.191, 244.798
*Edmontosaurus* BHI 126950 Plus 7.5%	935.83	0.959	897.458	−0.178, 1.101, 0	54.936, 183.743, 224.065
*Edmontosaurus* BHI 126950 Minus 7.5%	924.95	0.803	742.736	−0.314, 1.132, 0	40.286, 287.616, 318.421

**Table 8:** Results of mixed HAT segments sensitivity analysis.

Model	Thorax/neck	Tail	HAT CM			Whole body CM		
			Coordinates (x,y,z) (m)	Relative to hip joint (x,y) (m)	%body length craneal to hip joint	Coordinates (x,y,z) (m)		Relative to hip joint (x,y) (m)
*Tyrannosaurus rex* BHI 3033	Best estimate	Best estimate	−0.390, 3.07, 0	0.855, −0.046	7.33	−0.673, 2.815, 0	0.572, −0.297	4.9
*Tyrannosaurus rex* BHI 3033	Plus 15%	Minus 7.5%	−0.148, 3.05, 0	1.098, −0.062	9.41	−0.462, 2.830, 0	0.784, −0.282	6.71
*Tyrannosaurus rex* BHI 3033	Minus 7.5%	Plus 7.5%	−0.654, 3.08, 0	0.591, −0.035	5.06	−0.860, 2.824, 0	0.385, −0.288	3.3
*Tyrannosaurus rex* MOR 555	Best estimate	Best estimate	0.901, 2.614, 0	0.828, −0.052	7.47	0.541, 2.340, 0	0.468, −0.326	4.22
*Tyrannosaurus rex* MOR 555	Plus 15%	Minus 7.5%	1.119, 2.657, 0	1.045, −0.009	9.44	0.726, 2.394, 0	0.652, −0.272	5.89
*Tyrannosaurus rex* MOR 555	Minus 7.5%	Plus 7.5%	0.636, 2.593, 0	0.562, −0.074	5.08	0.368, 2.333, 0	0.295, −0.334	2.66
*Acrocanthosaurus atokensis* NCSM 14545	Best estimate	Best estimate	0.103, 2.279, 0.	0.472, −0.081	4.21	−0.046, 2.144, 0	0.322, −0.216	2.87
*Acrocanthosaurus atokensis* NCSM 14545	Plus 15%	Minus 7.5%	0.364, 2.234, 0.	0.732, −0.126	6.53	0.163, 2.122, 0	0.531, −0.237	4.74
*Acrocanthosaurus atokensis* NCSM 14545	Minus 7.5%	Plus 7.5%	−0.223, 2.309, 0	0.146, −0.051	1.3	−0.281, 2.170, 0	0.087, −0.190	0.78
*Struthiomimus sedens* 1266	Best estimate	Best estimate	0.310, 1.677, 0	0.324, −0.076	6.917	0.230, 1.504, 0	0.244, −0.249	5.21
*Struthiomimus sedens* 1266	Plus 15%	Minus 7.5%	0.397, 1.674, 0	0.411, −0.079	8.78	0.296, 1.518, 0	0.310, −0.235	6.63
*Struthiomimus sedens* 1266	Minus 7.5%	Plus 7.5%	0.235, 1.681, 0	0.249, −0.072	5.33	0.182, 1.506, 0	0.197, −0.247	4.2
*Edmontosaurus annectens* 126950	Best estimate	Best estimate	−0.195, 1.194, 0	0.409, −0.174	9.69	−0.304, 1.116, 0	0.299, −0.252	7.07
*Edmontosaurus annectens* 126950	Plus 15%	Minus 7.5%	−0.112, 1.154, 0	0.493, −0.215	11.66	−0.241, 1.010, 0	0.363, −0.273	8.61
*Edmontosaurus annectens* 126950	Minus 7.5%	Plus 7.5%	−0.261, 1.216, 0	0.344, −0.152	8.14	−0.343, 1.129, 0	0.262, −0.239	6.2

**Table 9:** Predicted hind limb mass proportions expressed as percentage of total body mass for models of each specimen.

Model	HAT	Legs	% hind limb mass
*Tyrannosaurus rex* BHI 3033	Best estimate	Best estimate	14.4
*Tyrannosaurus rex* BHI 3033	Plus 15%	Plus 15%	14.7
*Tyrannosaurus rex* BHI 3033	Plus 7.5%	Plus 7.5%	15.3
*Tyrannosaurus rex* BHI 3033	Minus 7.5%	Minus 7.5%	14.1
*Tyrannosaurus rex* BHI 3033	Plus 15%	Minus 7.5%	9.8
*Tyrannosaurus rex* BHI 3033	Minus 7.5%	Plus 15%	21.1
*Tyrannosaurus rex* MOR 555	Best estimate	Best estimate	16
*Tyrannosaurus rex* MOR 555	Plus 15%	Plus 15%	16.3
*Tyrannosaurus rex* MOR 555	Plus 7.5%	Plus 7.5%	16.7
*Tyrannosaurus rex* MOR 555	Minus 7.5%	Minus 7.5%	15.7
*Tyrannosaurus rex* MOR 555	Plus 15%	Minus 7.5%	11.4
*Tyrannosaurus rex* MOR 555	Minus 7.5%	Plus 15%	22.4
*Acrocanthosaurus atokensis* NCSM 14345	Best estimate	Best estimate	13.7
*Acrocanthosaurus atokensis* NCSM 14345	Plus 15%	Plus 15%	13
*Acrocanthosaurus atokensis* NCSM 14345	Plus 7.5%	Plus 7.5%	13
*Acrocanthosaurus atokensis* NCSM 14345	Minus 7.5%	Minus 7.5%	13.8
*Acrocanthosaurus atokensis* NCSM 14345	Plus 15%	Minus 7.5%	9.9
*Acrocanthosaurus atokensis* NCSM 14345	Minus 7.5%	Plus 15%	18
*Struthiomimus sedens* BHI 1266	Best estimate	Best estimate	18
*Struthiomimus sedens* BHI 1266	Plus 15%	Plus 15%	17.9
*Struthiomimus sedens* BHI 1266	Plus 7.5%	Plus 7.5%	17.8
*Struthiomimus sedens* BHI 1266	Minus 7.5%	Minus 7.5%	17.1
*Struthiomimus sedens* BHI 1266	Plus 15%	Minus 7.5%	12.4
*Struthiomimus sedens* BHI 1266	Minus 7.5%	Plus 15%	24.6
*Edmontosaurus annectens* BHI 126950	Best estimate	Best estimate	17.7
*Edmontosaurus annectens* BHI 126950	Plus 15%	Plus 15%	17.2
*Edmontosaurus annectens* BHI 126950	Plus 7.5%	Plus 7.5%	17.45
*Edmontosaurus annectens* BHI 126950	Minus 7.5%	Minus 7.5%	18.2
*Edmontosaurus annectens* BHI 126950	Plus 15%	Minus 7.5%	13.7
*Edmontosaurus annectens* BHI 126950	Minus 7.5%	Plus 15%	22.8

## Discussion

### Modelling approach

Our method of skeletal digitization and reconstructive modelling is fast, accurate and repeatable. All processing operations required to build the skeletal models from raw LiDAR data can be performed automatically by software programs (e.g. PolyWorks, RiSCAN PRO), allowing mathematically complex and time consuming processes to be carried out rapidly and efficiently. This makes the technique accessible to a wide audience of users and non-specialists, and minimises the impact of human error in the resulting models. This feature represents a major benefit and will be crucial to the wider application of the technique.

LiDAR's near infra-red laser is completely eye safe permitting its use in public galleries in museums without restricting access to the visiting public. To minimise interference between the scanner and the targeted skeletons, it is suggested that galleries be closed to the public. However, the facility to repeat and filter scans (so-called ‘scan sequence approach’; [see 50]) allows unwanted objects (such as passing people) to be systematically removed from the image data allowing scanning to be undertaken in busy periods when necessary. Filtering operations are also important to maintaining the manageability of the data sets. A multi-gigabyte data set can be generated in just a few hours scanning [49]–[51] and the automated filtering and decimation tools allow data size to be reduced with minimal cost to resolution. This allowed data collection, post-processing and modelling to be performed on a standard laptop computer.

**Figure 10:**
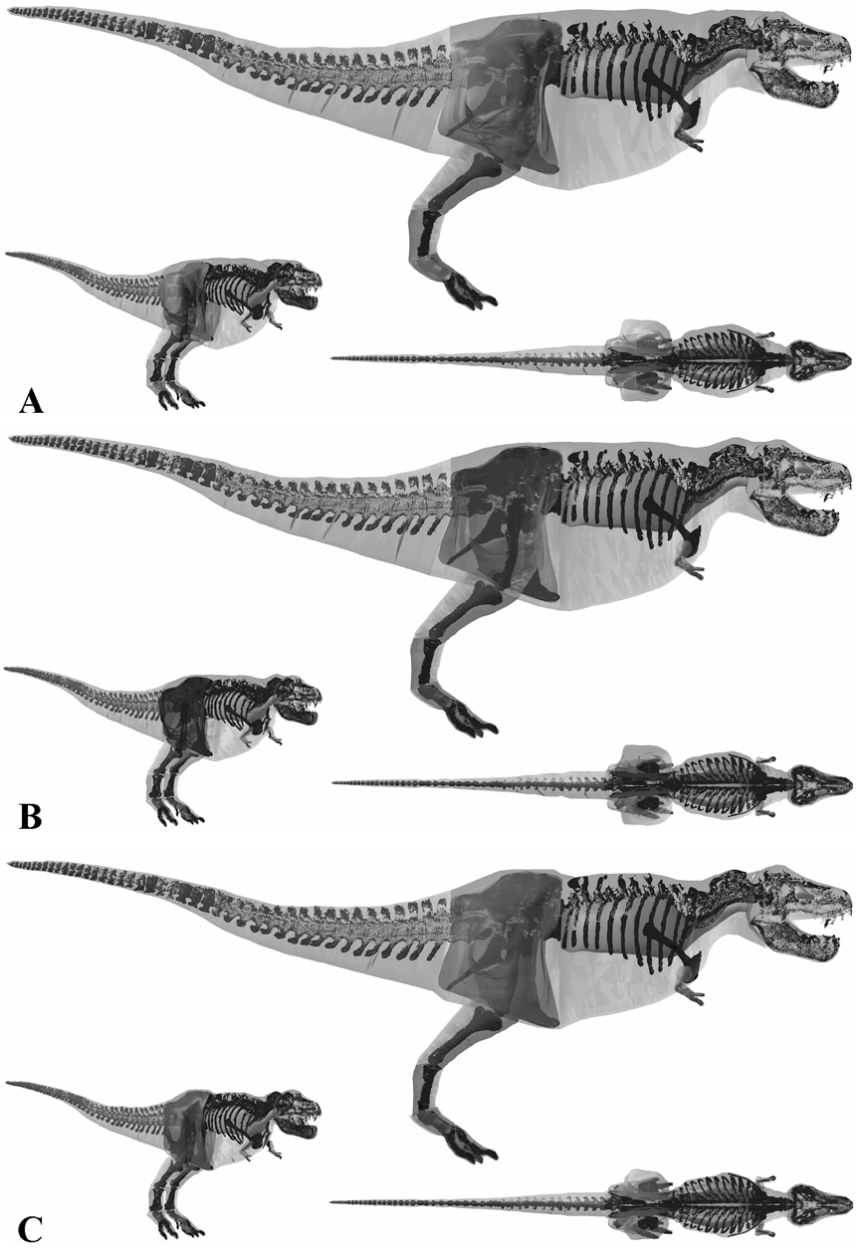
The three alternative models of *Tyrannosaurus rex* BHI 3033 in lateral, oblique right craniolateral and dorsal views. Neck, thoracic, sacral, tail and proximal hind limb segments have been increased by (A) 15% and (B) 7.5% in the two larger models, and (c) decreased by 7.5% in the smaller model.

**Figure 11:**
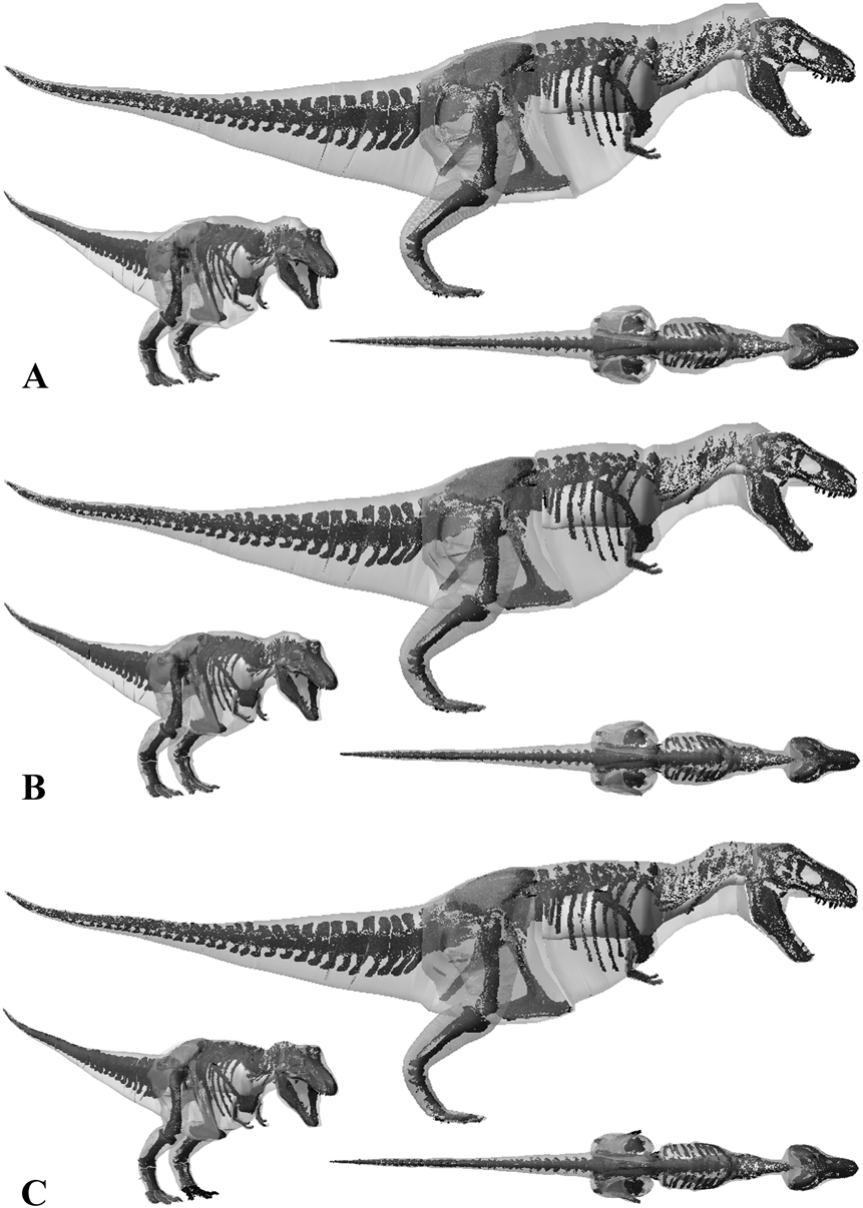
The three alternative models of *Tyrannosaurus rex* MOR 555 in lateral, oblique right craniolateral and dorsal views. Neck, thoracic, sacral, tail and proximal hind limb segments have been increased by (A) 15% and (B) 7.5% in the two larger models, and (c) decreased by 7.5% in the smaller model.

**Figure 12:**
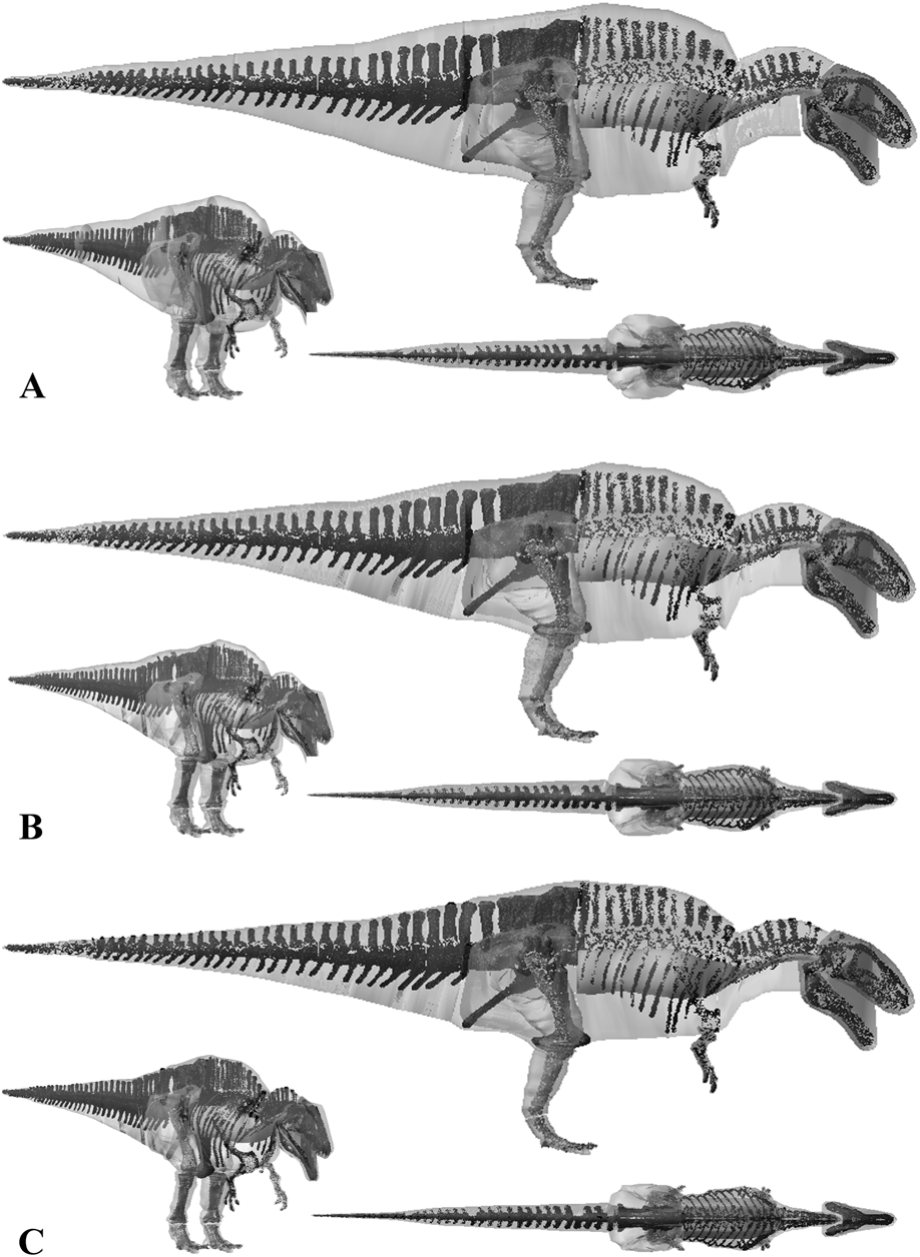
The three alternative models of *Acrocanthosaurus atokensis* NCSM 14345 in lateral, oblique right craniolateral and dorsal views. Neck, thoracic, sacral, tail and proximal hind limb segments have been increased by (A) 15% and (B) 7.5% in the two larger models, and (c) decreased by 7.5% in the smaller model.

**Figure 13:**
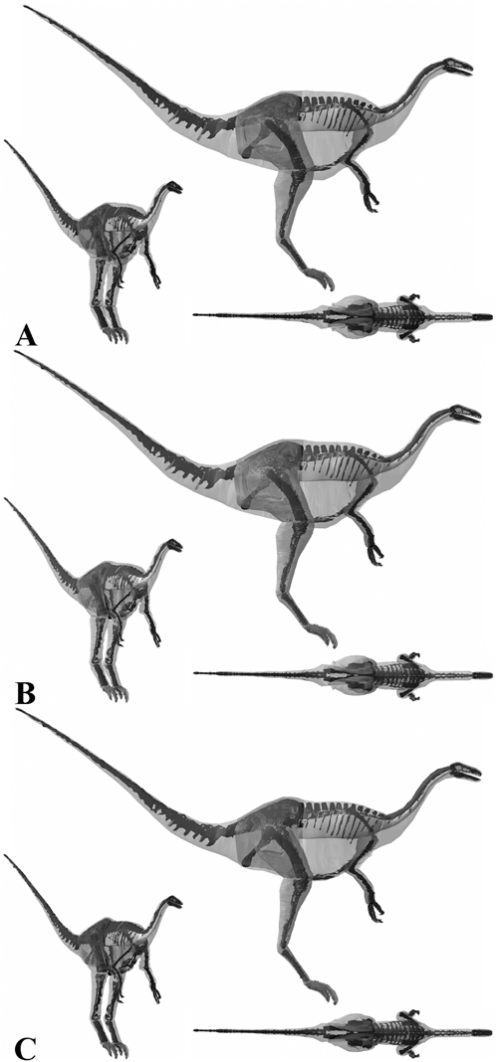
The three alternative models of *Struthiomimus sedens* BHI 1266 in lateral, oblique right craniolateral and dorsal views. Neck, thoracic, sacral, tail and proximal hind limb segments have been increased by (A) 15% and (B) 7.5% in the two larger models, and (c) decreased by 7.5% in the smaller model.

**Figure 14:**
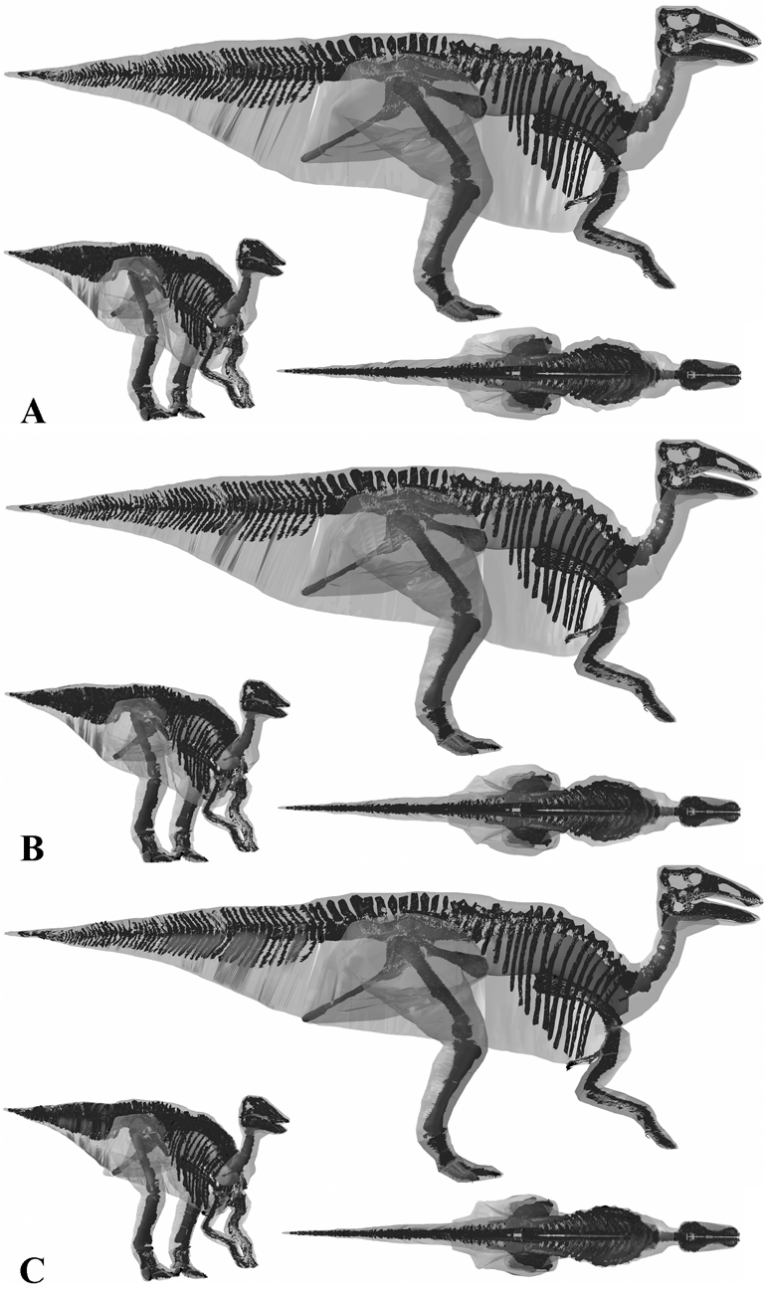
The three alternative models of *Edmontosaurus annectens* BHI 126950 in lateral, oblique right craniolateral and dorsal views. Neck, thoracic, sacral, tail and proximal hind limb segments have been increased by (A) 15% and (B) 7.5% in the two larger models, and (c) decreased by 7.5% in the smaller model.

The resolution offered by LiDAR point clouds was sufficient to capture the gross 3D geometry of the mounted skeletons and subsequently to guide reconstructions of body outlines and the geometry and placement of internal organs in the body and head (Figs 4, 5, 6, 7, 8, 9, 10, 11, 12, 13, 14). Bone surfaces are represented by millions of data points sampled directly from the specimen, which is clearly preferable to indirect digitization from literature-sourced photographs or drawings. Only the in case of *Tyrannosaurus rex* BHI 3033 was model resolution affected by constraints on data collection. The inability to scan from sufficient distances (i.e. plus 5 metres) from the specimen in lateral profile meant that the geometry of cervical and thoracic neural spines were captured at lower resolution than in other models. Although scan resolution in general is not sufficient to intricately model bone surface geometry, 3D data from CT or short range laser scanners can easily be incorporated into LiDAR models using either the alignment procedures described above or CAD tools.

The suite of modelling tools available within Maya meant that body volumes could be constructed in any shape and were not limited to strict ellipsoids or simple geometric shapes. The potential errors in estimations of mass parameters resulting from over-simplification of body outlines through the use of standard or uniform geometric shapes has been quantitatively demonstrated by Montani [60]. The automatic calculation of mass properties in Formz minimised the need for human calculation, which would have been extremely restrictive in terms of time and crucially would have limited the complexity of geometric shapes chosen to represent body and respiratory structure volumes. Our modelling approach therefore represents a highly accessible technique, one that may potentially be applied by a wide variety of researchers including those working on extant taxa. For those working on extinct animals it crucially allows the reconstructed body outline and internal organs to be displayed around the fossil skeleton, thereby offering explicit communication of the reconstruction and more meaningful comparisons with other models.

### Ostrich Validation

The volumetric reconstruction of an extant ostrich, based solely on a digitized mounted skeleton (BB. 3462), produced mass set predictions that closely match those published for this species [7], [58]. Smith et al. [58] measured a body mass of 70 kg for an adult ostrich (*Struthio camelus*), in which the lengths of the femora, tibiotarsus and tarsometatarsus were 0.28 m, 0.5 m and 0.45 m (Smith personal communication 2007). These are very close to lengths of the same segments in the ostrich digitized in this study (femora 0.26 m, tibiotarsus 0.471 and tarsometatarsus 0.426 m), which suggests that their overall mass properties should be comparable. It is therefore encouraging that the predicted body mass of our volumetric reconstruction (72.172 kg) essentially matches that measured by Smith et al. [58] for their specimen, although some caution is warranted as were unable to quantitatively validate estimated air sac volumes in our model. Similarly our reconstruction has 20.973% total body mass in a single limb, which closely matches the average value of 16.85% for total hind limb muscle mass in the ostrich [58]. Indeed, it is possible that removing bone volume from our reconstructed limb segments will bring this value closer to the measured hind limb muscle mass values of Smith et al. [58], which was obtained by summing the masses of dissected hind limb muscles rather than weighing whole limb segments. However, the predicted HAT CM does not closely correspond to published values for extant ostriches, being located 0.095 m craniad and 0.053 m ventral to the position calculated experimentally by Hutchinson et al. [7]. This discrepancy results from the manner in which mass has been apportioned between the thigh and posterior HAT segments in our model (Fig. 4D–E). Specifically, the sacral and post-sacral regions of the HAT segment are tightly constrained around the skeleton and the soft-tissue volume (corresponding to pelvic musculature) has been modelled as the proximal part of thigh segment (Fig.4D–E). If 50% thigh mass is included in the HAT segment then the latter CM shifts caudally to 0.089 m in front of the hip joint, matching the published calculation [7].

Whilst this demonstrates that our methodology is capable of producing broadly accurate predictions of mass properties in extant taxa with known morphology, it is again important to emphasize that this fact alone does not alter (i.e. enhance) the reliability of any single volumetric model of an extinct animal with unknown soft tissue morphology. Indeed, with numerous validation studies demonstrating accurate mass predictions of extant taxa from physical and digital volumetric models [4], [6]–[7; see above] we would argue that conducting sensitivity analyses on models of extinct taxa represents a far more significant measure of the extent to which meaningful mass predictions can be obtained for these animals.

### Dinosaur body dimensions

A century of research has proliferated body mass inferences for non-avian dinosaurs. Not surprisingly the majority of these studies have focused on *Tyrannosaurus rex*, and both MOR 555 [5], [7] and BHI 3033 [47] have been modelled in previous studies. Our reconstruction of *Edmontosaurus* (albeit a sub-adult) is the first of which we are aware for this genus. Henderson and Snively [59] provide the only body mass estimate for *Acrocanthosaurus* using digital modelling, and Christiansen and Farina [41] the only estimate for *Struthiomimus* using a physical model. Few studies have quantified the CM of these animals [4], [6]–[7] and only Hutchinson et al. [7] calculated inertial properties for the respective body segments of *Tyrannosaurus rex* MOR 555.

#### Body mass

Body mass results for *Tyrannosaurus rex* MOR 555 overlap those of previous workers. Our best estimate model (Fig. 6) of 6072 kg falls close to the 6583 kg obtained by Hutchinson et al. [7] and within the range of the upper estimates of Farlow et al. [5]. Our skinniest MOR 555 (Fig. 11c) has a total mass of 5580 kg (5543 kg with enlarged air sacs) but is highly emaciated, particularly in the torso, which when subjected to the full volume reduction actually invaded the rib cage. The largest MOR 555 (Fig. 11a) produced a mass estimate of 7700 kg (7997 kg with reduced air sacs), but is also highly unrealistic in many areas and contains an excessive amount of flesh around the torso, sacrum and proximal tail. However, all segments in the plus 7.5% model (Fig. 11b) still appear fairly reasonable, and we consider the total mass of 6956 kg perfectly valid for this animal. We therefore suggest the total body mass of MOR 555 is well constrained within 5750–7250 kg, as was similarly suggested by Hutchinson et al. [7]. However, it is noteworthy that the mass values obtained here for many of the individual body segments of MOR 555 differ significantly from those of Hutchinson et al. [7]. This largely emphasises degree of subjectivity and artistic freedom available when constructing these models. The larger neck cavity in our study may be partly explained by the smooth continuous transition between the thoracic and neck segments. In our models we reconstructed the ventral outline of the body passed smoothly under the scapula-coracoids, while Hutchinson et al. [7] chose a sharp inflexion in both the dorsal and ventral profile at the junction between the thoracic and neck segments thereby deceasing volume relative to our model. In our models we also chose to extend the neck to the ventral and dorsal surfaces of the head, rather than inserting solely into the posterior face of the head segment. By contrast the thoracic segment of Hutchinson et al. [7] is significantly larger than the equivalent segments (sacral and thoracic) in our model. The sacral and thoracic segments from the best estimate obtained in this study have a combined volume of 2.38 m^3^ compared to 4.19 m^3^ of Hutchinson et al. [2007]. Without skeletal landmarks figures it is difficult to judge the extent of the body outline relative to the skeleton in the model of Hutchinson et al. [7] and hence to make a fair comparison to our model. With the hind limb fully straightened beneath the hip joint the ventral outline of the body passes below the knee joint in the model of Hutchinson et al. [7]. By contrast, our outline passes close to the pelvis (ischium and pubis) even with the knee slightly flexed, based on consideration of pelvic musculature and the impressions of the pubic boot in trace fossils [61]. Around the pectoral girdle the lateral profile has to pass under scapula-coracoids and is unlikely to extend below the level of the arms, which would severely restrict their range of movement. The gastralia form a shallow convexity linking the pubis and sternum in non-avian theropods and we constructed the belly outline with a modest amount of flesh beneath this plane, based on the relationship between the gastralia and the abdominal wall in extant crocodilians [62]–[64]. We suspect that the greatest difference between the respective reconstructions is likely to lie in the mediolateral plane, which is generally considered to be the most uncertain dimension in trunk reconstructions of non-avian dinosaurs [6], [59]. Our best estimate reconstruction of MOR 555 had around 60 mm of soft tissue between the proximal end of the ribs and the wall of the thoracic cavity, which represents 17.8% of the mediolateral width of one side of the body. A similar ratio was obtained in all our best estimate models, but it is again difficult to compare this to previous reconstructions as we are the first to quantify the mediolateral extent of our volumetric reconstructions relative to the fossil skeletons. All five axial skeletons modelled here remained articulated in poses of the physical mounts, and the ‘accuracy’ of these reconstructions will inevitably influence the size and shape of body outlines in any physical or digital volumetric model. Particular uncertainty exists in the placement and orientation of the scapulocoracoids, forelimbs and ribs in non-avian dinosaurs, while correct spacing between vertebrae (representing the volume occupied by the intervertebral discs) is similarly unknown. Although well beyond the scope of this study, the ability to segment and re-articulate the digital skeleton means it would be perfectly possible to extend the sensitivity analysis to quantify the effects of the choice of skeletal articulation on mass set results.

The volume and hence mass assigned to hind limb segments is also an equally controversial aspect of non-avian dinosaur biology (see below). Only the shank segment of our best estimate model of MOR 555 (0.212 m^3^) is similar to the 0.208 m^3^ estimated by Hutchinson et al. [7]. Our larger thigh segment is probably explained by the manner in which we have chosen to model its attachment to the sacral segment. In all our models the thigh segments are expanded proximally (at the expense of the sacral segment) to encompass the volume for hypothesized tail and pelvic musculature inserting on the hind limb [65]. This proximal expansion appears larger than that in Hutchinson et al. [7], as do the hamstrings of our model, but again this is difficult to assess as their best estimate model is figured without the CT scans of the hind limb skeleton. Perhaps the most unexpected disparity occurs in head segment, where the final best estimate volume of Hutchinson et al. [7] is just 62.2% of our estimate of 0.661 m^3^, with the disparity in the final mass value increased by our smaller zero-density air cavities. We constructed a head volume that wrapped tightly around the skull without invading its surfaces. It is possible that all our theropod head volumes are slightly too low, as we did not greatly expand the cavity outline around the paraoccipital crest. The possession of crescent-shaped, expanded paraoccipital process in these theropods indicates a large muscle attachment at the back of the skull [66], and it is possible that the neck muscles attaching to the occiput achieved a thicker cross section than we account for in our best estimate reconstructions.

The skeleton of *Tyrannosaurus rex* BHI 3033 is considerably larger than MOR 555, for example body length is approximately 11.9 m versus 11.1 m (estimated from models). It is therefore no surprise that our best estimate model (Fig. 5) has a significantly higher total body mass of 7655 kg. Our best estimate reconstruction of BHI 3033 contrasts starkly with that of Stevens et al. [46], despite using an identical modelling approach. Stevens et al. [46] estimate of approximately 4400 kg falls well below our skinniest reconstruction (Fig. 10c) of 6905 kg (6777 kg with enlarged air sacs), in which the torso cavity was tightly oppressed to the rib cage. Indeed, we feel that our skinniest model is also too slender in the tail, neck and hind limb (Fig. 10c) thereby casting extreme doubt on estimates below 7000 kg for this animal. However, it should be noted that Stevens et al. [46] were principally concerned with calculating the CM of BHI 3033 rather than a robust total body mass value. As with our other models, the segment volumes of our plus 7.5% model (Fig. 10b) appear perfectly plausible given the inherent uncertainties, and so total body mass estimates of around 8899 kg appear to be reasonable. However, our plus 15% model (Fig. 10a) appears to have an excessive amount of flesh around all its HAT segments, suggesting the value of 9940 kg (10134 kg with reduced air sacs) far exceeds the maximum for this animal. We suggest the likely mass value lies in the range 7250–9000 kg. Overall, our reconstructions add to the growing convergence of estimates above 5750 kg total body mass for *Tyrannosaurus*, depending on size of individual studied [5]–[7].

The lack of attention received by non-Tyrannosaurid dinosaurs means there is little comparative mass data on *Acrocanthosaurus*, *Stuthiominmus* and *Edmontosaurus*. Our best estimate model of *Acrocanthosaurus* (Fig. 7) is heavier than that of Henderson and Snively [58] who estimated 5672 kg for NCSM 14345. Henderson and Snively [59] set the density of the post-cervical region to 1000 kg m^3^, and the cranio-cervical region to 900 kg m^3^ to account for pneumatization of the skeleton and associated air sacs. They also included a single zero density lung in the thoracic region, which measured approximately 10% body volume. This model of *Acrocanthosaurus* NCSM 14345 has subsequently been modified following recent work [53]–[54] on the skeletal pneumatization and pulmonary anatomy of non-avian theropods (Henderson personal communication 2008). In this new model, the volume surrounding the large neural spines in the sacral to cervical region (‘sagittal crest’) have been modelled with a density of 1000 kg m^3^, while the remainder of the pre-sacral volume has been set at 900 kg m^3^, thereby lowering the original mass estimate to 5072 kg (Henderson personal communication 2008). Approximate segmentation of this revised model suggests our best estimate model has a significantly larger tail volume (1.149 m^3^ versus ∼0.679 m^3^). Such a large disparity is perhaps not surprising given that the high-level of uncertainty surrounding the mediolateral extent of post-sacral body cavities (see above) applies equally to the tail segment. However, we feel that the mediolateral extent of our best estimate tail volumes are in fact quite conservative, particularly in the regions proximal to the transition point housing the large caudofemoralis musculature [67]–[68]. Certainly we feel the mediolateral extents of the tail volumes in our plus 7.5% models remains within the realistic range for each of the theropods, including *Acrocanthosaurus*. Our fore- and hind limbs also differ significantly; whilst our best estimate hind limbs (0.847 m^3^) are much larger than the 0.582 m^3^ reconstructed by Henderson and Snively [59], our fore limbs are a mere one-third the volume (0.012 m^3^ versus 0.036 m^3^). Henderson and Snively's [59] revised pre-sacral volume measures approximately 3.742 m^3^ and is therefore only moderately smaller than our value of 4.053 m^3^ for *Acrocanthosaurus* (i.e. 92.3% our volume). However, subtracting the lung volume gives a net density of 776.14 m^3^ and a mass of 2904 kg for Henderson and Snively's [59] modified post-sacral reconstruction (Henderson personal communication 2008). Rather than reduce tissue density to 900 kg m^3^ we included the full suite of hypothesised air sacs [52]–[53] in our models (Fig. 3), which reduced the net density of the post-sacral region to 847.52 kg m3 and the mass to 3435 kg. Thus it is largely this density contrast that is responsible for significant disparity in predicted body mass between the two models, such that Henderson and Snively's [59] post-sacral reconstruction has a mass of 84.6% of our best estimate model. The resulting total body value of 5072 kg is in fact considerably lower than our most gracile *Acrocanthosaurus* (5570 kg, or 5473.96 kg with enlarged air sacs), which we consider to be unreasonably emaciated in all HAT and proximal hind limb segments (Fig. 12c). As with the two *Tyrannosaurus* models, our plus 7.5% model (Fig. 12b) remains within the likely maximum range for body volumes, suggesting 7000 kg is not impossible for this animal. However, the largest model created appears highly implausible, having an unrealistic amount of external flesh around all its HAT segments (Fig. 12a). We therefore suggest 5750–7250 kg represents a plausible maximum body mass range for this specimen of *Acrocanthosaurus*.

Although no comparative data exists on *Struthiomimus sedens*, Christiansen and Farina [41] estimated 175 kg for *Strutiomimus altus* AHNM 5339 using a physical model, but the smaller skeleton of this individual (e.g. femoral length 486 mm versus 662 mm in *Struthiomimus sedens* 1266) makes meaningful comparisons difficult. Sensitivity analysis of body segment volumes produced a suite of models that in fact remained fairly reasonable in appearance throughout the full range tested (Figs 8 & 13), although the thoracic segment of the smallest model does appear unrealistically skinny (Fig. 13c). Whilst we feel our best estimate model (423 kg) represents the most realistic created (Fig. 8), we are less confident in assigning a realistic range for body mass than with the other theropods. Our largest model (Fig. 13a) has a mass of 524 kg (529 kg with reduced air sacs) and does not appear to have an unrealistic amount of flesh around the skeleton, despite the 22.6% increase in volume over the best estimate model. Although we consider the skinniest model (Fig. 13c) estimate of 381 kg (376 kg with enlarged air sacs) still to be valid based on uncertainties, it must be close to the minimum value as parts of the proximal tail and thorax are tightly pressed to the skeleton.

The volumetric proportions of body segments in our reconstructions of *Struthiomimus* differ significantly from those of the larger theropods studied. The reduction of tail and associated caudofemoralis musculature in derived non-avian theropods [67]–[68] is reflected in tail of our best estimate model which is 12.3% of the HAT volume, significantly less than the range of 17.7–23.4% estimated for the three larger theropods modelled. The relative contribution of the fore limbs was considerably greater in the best estimate model of *Struthiomimus*, measuring a combined 5.56% of the total HAT volume, versus 0.42% BHI 3033, 0.44% in MOR 555 and 0.47% in NCSM 14345. The relative volumes of the proximal hind limb segments of our best estimate *Struthiomimus* also differ significantly from the other theropods. In *Struthiomimus* the thigh segment is only 2.5 times larger than the shank, while the best estimate reconstructions of the more primitive theropods all have thigh segments more than three times larger than their shank segments (4.7 times larger in *Acrocanthosaurus*).

Although a similar body length to *Struthiomimus*, our best estimate reconstruction of *Edmontosaurus* (Fig. 9) is approximately twice the mass at 813 kg, owing largely to the significantly greater dorsoventral depth of the body segments. As with our theropod models, we reconstructed the ventral outline of the body close to the skeleton around the pelvic and pectoral girdles (Fig. 9a&c), based on previous myological reconstructions [69]–[70]. Whilst this helped constrain the likely ventral profile in the thoracic segment between the pubes and sternum, the dorsoventral depth given to the tail remained particularly subjective. Despite this level of uncertainty we feel our largest model (984 kg, or 994 kg with a reduced lung) considerably overestimates HAT volume, having a ventral profile that extends too far below the axial skeleton (Fig. 14a). In contrast to the theropods modelled, our smallest *Edmontosaurus* (743 kg, or 732 kg with an enlarged lung) retains a realistic ventral profile, albeit with extremely little flesh around the distal ischium (Fig. 14c). However, the HAT segments, particularly the thoracic volume, are tightly pressed mediolaterally against the skeleton, casting extreme doubt on the mass estimation. Given these reconstructions we suggest 775–925 kg represents a reasonable range for total body mass of this individual.

The results of our sensitivity analysis of air sac volumes largely concurs with previous analyses and assertions that suggest errors in lung volumes will have relatively little effect on body mass predictions in dinosaurs [7], [44]. Our initial air sacs ranged from 6.8–9.6% of total best estimate body volumes (or 10.2–12.9% HAT volume) in non-avian theropods and 7.8% (11.6% HAT volume) in *Edmontosaurus*. Larger body air sacs increased this volume to 7.9–11.1% in non-avian theropods and 9% in *Edmontosaurus*, while smaller air sacs ranged from 5.4–7.3% and 6.6% best estimate body volumes. Changing air sac volumes in the largest and smallest models to exaggerate mass effects had less than +/−2% effect on total body mass in these models. The caudal extent of the thoracic airs sacs lies just in front of the pelvis in each of the non-avian theropods modelled, which may be conservative for *Tyrannosaurs* and *Struthiomimus* based on evidence from skeletal pneumatisation [71]. Addition of an abdominal air sac to our best estimate models (Supporting Information Tables S1: 1–49) had a modest affect on mass predictions, reducing total body by between 1.3–2.98% in the non-avian theropods.

#### Centres Of Mass (CM)

**Figure 15:**
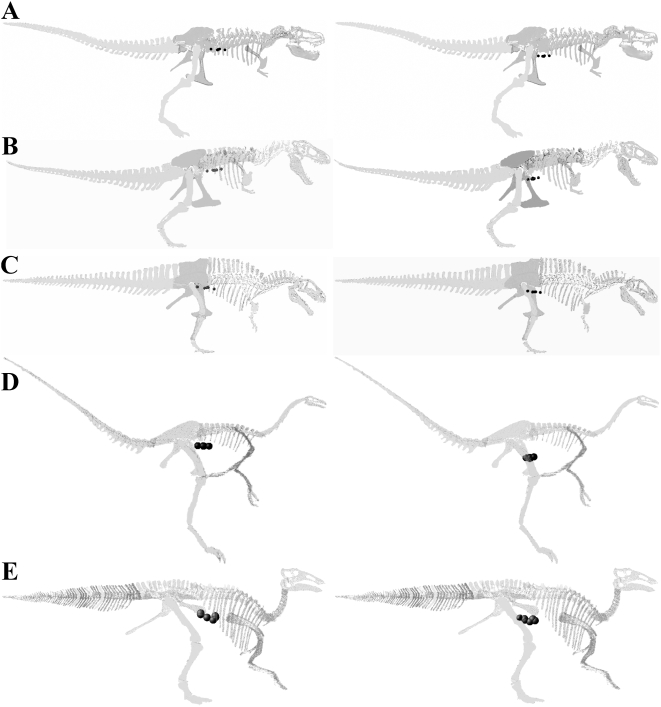
HAT (left) and whole body (right) centres of mass for each model of (A) *Tyrannosaurus rex* BHI 3033, (B) *Tyrannosaurus rex* MOR 555, (C) *Acrocanthosaurus atokensis* NCSM 14345, (D) *Struthiomimus sedens* BHI 1266 and (E) *Edmontosaurus annectens* BHI 126950 (not to scale).

As with body mass estimates, the majority of published CM predictions are for *Tyrannosaurus*, and our data set provides important new information on taxa from other dinosaurian groups. Our sensitivity analysis demonstrates that the whole body CM must lie well in front and below the hip joint in all five taxa studied (Table 8, Fig. 15), and therefore probably in all dinosaurian groups. Even in models with significantly enlarged tails and reduced thoracic and neck segments the CM still remained comfortably in front of the hip joint. Whilst this general conclusion has been reached before about CM positions, only Hutchinson et al. [7] have demonstrated that it is upheld within the bounds of uncertainties regarding body and air sac volumes as we do here. The best estimate MOR 555 of Hutchinson et al. [7] has a CM 0.51 m cranial of the hip joint, very close to the position (0.468 m) in our reconstruction. Our range of CM values for MOR 555 partially overlap that of Hutchinson et al. [7], with our more caudally distributed range (0.295–0.652 m cranial of the hip joint) explained by our smaller thoracic volume (see above).

Varying the volume of thoracic and pharyngeal air sacs had a relatively modest effect on CM positions in all five animals models (Supporting Information Tables S1: 26–49). For example, smaller and larger air sacs generally shifted the CM by just +/−0.01 m along x and y axes in *Edmontosaurus* and *Struthiomimus*, and on average by around +/−0.03 m in *Acrocanthosaurus* and the two Tyrannosaurs. This largely reflected the fact that air sac volumes were modified by simply raising or lowering ventral base of the cavities, rather expanding or contracting the volume in three dimensions as in previous studies [e.g. 7].

#### Moments Of Inertia

Hutchinson et al. [7] present the only comparable data set on inertial properties for a non-avian dinosaur. However, comparing the two data sets is difficult as we only calculate the moments of inertia for our combined HAT segments of MOR 555 versus the whole body calculation in Hutchinson et al. [7]. These authors do state the principal moments of the HAT segment of their skinniest model, which are understandably lower than our best estimates given the lower mass estimate, particularly for I_yy_ and I_zz_.

As stated previously, inertial values showed a positive correlation with body mass such that the heaviest models consistently had the largest principal moments of inertia. Differences in the relative values of principal moments across the studied animals appear to be size based rather than taxonomic. The ratios of the principal moments of best estimate models of BHI 3033 (0.07: 0.96: 1), MOR 555 (0.06: 0.94: 1) and *Acrocanthosaurus* (0.05: 0.97: 1) are almost identical, and contrast with the relatively higher values for I_xx_ attained for the smaller *Struthiomimus* (0.13: 0.90: 1) and *Edmontosaurus* (0.13: 0.90: 1). This may add support to the idea that larger theropods possessed body shape that minimized rotational inertia relative to smaller taxa [59], but clearly a more detailed analysis is required to evaluate this thoroughly.

### Mass predictions and biomechanical modelling

Information on the 3D distribution of mass is fundamental to biomechanical assessments in both extant and extinct taxa [36], and in recent times this data has been used in a variety of functional appraisals of non-avian dinosaurs. Using static models Hutchinson [56] and colleagues [7], [33] have demonstrated that values chosen for the CM and particularly the ratio of hind limb muscle mass to total body mass have a significant effect on the level of locomotor ability of bipedal non-avian dinosaurs. Sellers and Manning [35] performed a dynamic analysis of locomotion in the same taxa and used sensitivity analysis to demonstrate a positive correlation between hind limb muscle mass and locomotor ability in *Tyrannosaurus*. This sensitivity analysis was extended by Bates [57] who similarly demonstrated that the ratio of hind limb muscle mass to total mass has the single greatest effect on predictions of maximum running speed in bipedal dinosaurs.

Comparable published data on hind limb muscle mass in extant vertebrates is scarce, and many studies omit the total body mass of the specimens making it impossible for the ratio to be determined. The majority of studies weigh whole limb segments (i.e. including bones), which although not measures of muscle mass, are directly comparable to our models in which limb segments have been given uniform density. Our best estimate model of MOR 555 has a single hind limb mass equivalent to 16% total body mass (Table 9), close to the 14.2% estimated by Hutchinson et al. [7] for this animal. The revised *Acrocanthosaurus* model of Henderson and Snively [59] has 11.5% total body mass in a single hind limb, slightly lower than the 13.7% we estimate here. The best estimate models of the three large theropods therefore have a range of 13.7–16%, while *Struthiomimus* (18%) and *Edmontosaurus* (17.7%) have slightly higher values (Table 9), as might be predicted by their smaller size [56]. These values are substantially lower than the best estimate values used for non-avian theropods in analyses of Hutchinson [56] and Sellers and Manning [35]. These models had approximately 23.85% muscle mass in each hind limb, which exceeds the highest estimates possible for larger theropods using the body segment combinations created here. However these values are not strictly comparable since some of this muscle mass is contained in the HAT segment rather than in the legs. Only the model of *Struthiomimus* composed of the largest limb and smallest HAT volumes produces a ratio above 0.24. This casts significant doubt on maximum running speeds above 12 m/s for the largest non-avian theropods like *Tyrannosaurus*, based on current simulations. However, it should be noted that although our hind limb masses are inclusive of bone volume, they do not include the sizable contribution of major tail-based hip extensors such as the caudofemoralis longus. Clearly more precise values for dinosaur locomotor muscle mass should be sought by separating out bone volume and including tail-based musculature and this should be possible in the CAD environment. That said, we feel it unlikely that significantly higher values (i.e. plus 20% total body mass per limb) are realistic for medium to large non-avian theropods.

The relatively conservative range of CM values attained in this study is of some reassurance to those studying dinosaur locomotor biomechanics. In our previous work, varying the longitudinal position of the trunk or HAT CM had little effect on the predicted maximum running speed of *Allosaurus* [57], despite testing a relative range that extended significantly closer and farther cranial to the hip joint than predicted for taxa in this study (Table 8, Fig. 15). However, trunk orientation was not constrained in the relatively simple anatomical model of *Allosaurus* used in this investigation and subsequently the model responded to CM changes by progressively increasing the angle of the trunk with respect to the ground thereby maintaining the proximity of CM to the hip joint on the longitudinal axis. The development of anatomically realistic articulated digital models, such as those in this study, will allow the internal range of motion within the trunk to be constrained within realistic bounds in future locomotor simulations. When combined with sensitivity analyses like the one conducted here, a more detailed examination of the effects of CM positions in non-avian dinosaurs on locomotor mechanics will be possible.

### Conclusions

Our modelling approach represents a highly flexible non-invasive technique for estimating the mass properties of extinct animals, which can be equally well applied to extant forms. The high level of automated processing and data extraction greatly simplifies mathematically complex and time-consuming processes and simultaneously minimises the potential for human error. The rapidity with which models can be manipulated and modified has allowed a comprehensive evaluation of the full suite of mass set properties for five specimens of four species of non-avian dinosaurs using a detailed sensitivity analysis. This analysis allowed maximum likely plausible ranges of mass set values to be identified for each taxa, accounting for the effects of inevitable unknowns in these reconstructions. The importance of sensitivity analyses is emphasized further when mass set values are applied to biomechanical assessments of non-avian dinosaurs. Clearly, future biomechanical assessments of extinct taxa should be preceded by a detailed investigation of the plausible range of mass properties, in which sensitivity analyses are used to identify a suite of possible values to be tested as inputs in the biomechanical model [7], [57]. This emphasises that higher level biomechanical and evolutionary analyses of extinct taxa should be conducted in an iterative fashion, with on-going critical evaluation of mass and soft tissue properties used in analytical models.

## Supporting Information

Tables S1 Supplementary data tables to published in on-line appendix
(2.07 MB DOC)
