# Supplementary material for: Estimating Mass Properties of Dinosaurs Using Laser Imaging and 3D Computer Modelling
Source: PLoS One. 2009 Feb 19;4(2):e4532. doi: 10.1371/journal.pone.0004532 (PMC2639725; doi:10.1371/journal.pone.0004532)
Supplement: Tables S1 — Supplementary data tables to published in on-line appendix (2.07 MB DOC) [file pone.0004532.s001.doc]

Table S1. Results for the Plus 15% best estimate model of *Tyrannosaurus rex* BHI 3033.

| Segment | Net Density (kg m-3) | Volume (m3) | Mass (kg) | CM (x,y,z) (m) | Ixx Iyy Izz (kg m2) |
| --- | --- | --- | --- | --- | --- |
| Head | 990.6 | 0.685 | 678.561 | 2.552, 3.346, 0 | 156.657, 6082.01, 6185.55 |
| Air sacs | 0 | 0.01 | 0 | - | - |
|  |  |  |  |  |  |
| Neck | 926.17 | 0.472 | 437.151 | 1.701, 3.367, 0 | 112.285, 2011.3, 2083.61 |
| Pharyngeal cavity | 0 | 0.035 | 0 | - | - |
|  |  |  |  |  |  |
| Thorax | 689.41 | 4.72 | 3254.13 | 0.394, 2.949, 0 | 1517.66, 3542.39, 4270.27 |
| Lungs | 0 | 0.764 | 0 | - | - |
|  |  |  |  |  |  |
| Sacrum | 1000 | 1.166 | 1166.031 | -1.335, 2.984, 0 | 350.399, 1253.35, 1565.93 |
|  |  |  |  |  |  |
| Tail | 1000 | 1.468 | 1467.751 | -0.751, 3.079, 0 | 221.74, 16386.5, 16556.6 |
|  |  |  |  |  |  |
| Arm | 1000 | 0.011 | 10.931 | 1.138, 2.398, 0.483 | 7.336, 29.337, 31.394 |
| Digit I | 1000 | 0.001 | 0.644 | 1.315, 2.115, 0.62 | 0.806, 2.200, 2.507 |
| Digit II | 1000 | 0.001 | 0.548 | 1.33, 2.197, 0.591 | 0.586, 1.879, 2.081 |
| Total Fore limb | 1000 | 0.013 | 12.123 | 1.156, 2.374, 0.495 | 8.728, 33.412, 35.982 |
|  |  |  |  |  |  |
| Thigh | 1000 | 1.004 | 1004.155 | -1.337, 2.857, 0.46 | 263.403, 129.471, 319.34 |
| Shank | 1000 | 0.287 | 287.174 | -1.899, 1.69, 0.366 | 20.447, 32.873, 43.870 |
| Metatarsus | 1000 | 0.096 | 96.095 | -0.244, 0.921, 0.307 | 4.693, 2.604, 4.378 |
| Digit II | 1000 | 0.023 | 22.332 | -1.897, 0.11, 0.29 | 0.257, 0.259, 0.499 |
| Digit III | 1000 | 0.027 | 26.564 | -1.811, 0.134, 0.457 | 0.308, 0.540, 0.781 |
| Digit IV | 1000 | 0.021 | 20.966 | -1.998, 0.09, 0.571 | 0.241, 0.242, 0.452 |
| Pes | 1000 | 0.07 | 69.863 | -2.112, 0.681, 0.72 | - |
| Hind limb | 1000 | 1.457 | 1457.29 | -1.226, 2.229, 0.444 | - |
|  |  |  |  |  |  |
| HAT | 868.25 | 8.092 | 7025.86 | -0.424, 3.045, 0 | 2376.198, 29342.41, 30733.88 |
| Whole Body | 903.18 | 11.006 | 9940.43 | -0.659, 2.806, 0 | - |

Table S2. Results for the Plus 7.5% best estimate model of *Tyrannosaurus rex* BHI 3033.

| Segment | Net Density (kg m-3) | Volume (m3) | Mass (kg) | CM (x,y,z) (m) | Ixx Iyy Izz (kg m2) |
| --- | --- | --- | --- | --- | --- |
| Head | 990.6 | 0.685 | 678.561 | 2.553, 3.346, 0 | 151.59, 6025.07, 6123.54 |
| Air sacs | 0 | 0.01 | 0 | - | - |
|  |  |  |  |  |  |
| Neck | 917.07 | 0.417 | 382.418 | 1.698, 3.381, 0 | 92.1024, 1730.26, 1791.86 |
| Pharyngeal cavity | 0 | 0.035 | 0 | - | - |
|  |  |  |  |  |  |
| Thorax | 779.86 | 3.47 | 2706.12 | 0.381, 2.954, 0 | 1126.47, 2773.87, 3330.95 |
| Lungs | 0 | 0.764 | 0 | - | - |
|  |  |  |  |  |  |
| Sacrum | 1000 | 1.114 | 1113.67 | -1.328, 2.995, 0 | 307.523, 1203.05, 1475.72 |
|  |  |  |  |  |  |
| Tail | 1000 | 1.267 | 1267.278 | -3.535, 3.088, 0 | 173.975, 14220.8, 14356.3 |
|  |  |  |  |  |  |
| Arm | 1000 | 0.011 | 10.931 | 1.138, 2.398, 0.483 | 7.510, 28.854, 31.086 |
| Digit I | 1000 | 0.001 | 0.644 | 1.315, 2.115, 0.62 | 0.821, 2.165, 2.490 |
| Digit II | 1000 | 0.001 | 0.548 | 1.33, 2.197, 0.591 | 0.597, 1.851, 2.065 |
| Total Fore limb | 1000 | 0.013 | 12.123 | 1.156, 2.374, 0.495 | 9.108, 27.928, 30.878 |
|  |  |  |  |  |  |
| Thigh | 1000 | 0.963 | 962.577 | -1.333, 2.855, 0.458 | 245.816, 120.635, 298.198 |
| Shank | 1000 | 0.248 | 248.133 | -1.894, 1.689, 0.361 | 16.6462, 26.4, 36.012 |
| Metatarsus | 1000 | 0.084 | 84.008 | -2.436, 0.918, 0.303 | 3.996, 1.924, 3.715 |
| Digit II | 1000 | 0.023 | 22.332 | -1.897, 0.11, 0.29 | 0.257, 0.259, 0.499 |
| Digit III | 1000 | 0.027 | 26.564 | -1.811, 0.134, 0.457 | 0.308, 0.540, 0.781 |
| Digit IV | 1000 | 0.021 | 20.966 | -1.998, 0.09, 0.571 | 0.241, 0.242, 0.452 |
| Pes | 1000 | 0.07 | 69.863 | -2.112, 0.681, 0.72 | - |
| Hind limb | 1000 | 1.365 | 1364.58 | -1.37064, 2.25888, 0.444218 | - |
|  |  |  |  |  |  |
| HAT | 852.72 | 7.236 | 6170.29 | -0.410, 3.06, 0 | 1869.518, 26018.87, 27149.74 |
| Whole Body | 893.07 | 9.965 | 8899.45 | -0.704, 2.813, 0 | - |

Table S3. Results for the Minus 7.5% best estimate model of *Tyrannosaurus rex* BHI 3033.

| Segment | Net Density (kg m-3) | Volume (m3) | Mass (kg) | CM (x,y,z) (m) | Ixx Iyy Izz (kg m2) |
| --- | --- | --- | --- | --- | --- |
| Head | 990.6 | 0.685 | 678.561 | 2.553, 3.346, 0 | 142.375, 5754.62, 5843.88 |
| Air sacs | 0 | 0.01 | 0 | - | - |
|  |  |  |  |  |  |
| Neck | 896.83 | 0.342 | 306.716 | 1.707, 3.373, 0 | 56.1182, 1312.27, 1350.36 |
| Pharyngeal cavity | 0 | 0.035 | 0 | - | - |
|  |  |  |  |  |  |
| Thorax | 719.33 | 2.722 | 1958.02 | 0.372, 2.981, 0 | 661.541, 1736.12, 2062.65 |
| Lungs | 0 | 0.764 | 0 | - | - |
|  |  |  |  |  |  |
| Sacrum | 1000 | 1.044 | 1044.22 | -1.315, 3.003, 0 | 266.649, 1221.98, 1457.95 |
|  |  |  |  |  |  |
| Tail | 1000 | 0.942 | 942.361 | -3.526, 3.099, 0 | 104.913, 10916.1, 10999.7 |
|  |  |  |  |  |  |
| Arm | 1000 | 0.011 | 10.931 | 1.138, 2.398, 0.483 | 7.8551, 26.593, 29.1693 |
| Digit I | 1000 | 0.001 | 0.644 | 1.315, 2.115, 0.62 | 0.849, 2.016, 2.370 |
| Digit II | 1000 | 0.001 | 0.548 | 1.33, 2.197, 0.591 | 0.620, 1.724, 1.960 |
| Total Fore limb | 1000 | 0.013 | 12.123 | 1.156, 2.374, 0.495 | 9.324, 30.333, 33.499 |
|  |  |  |  |  |  |
| Thigh | 1000 | 0.661 | 660.703 | -1.329, 2.851, 0.386 | 163.536, 65.89, 198.758 |
| Shank | 1000 | 0.184 | 183.605 | -1.881, 1.684, 0.352 | 11.389, 17.820, 25.388 |
| Metatarsus | 1000 | 0.062 | 62.105 | -2.427, 0.911, 0.293 | 2.677, 1.094, 2.599 |
| Digit II | 1000 | 0.023 | 22.332 | -1.897,0.11,0.29 | 0.257, 0.259, 0.499 |
| Digit III | 1000 | 0.027 | 26.564 | -1.811, 0.134, 0.457 | 0.308, 0.540, 0.781 |
| Digit IV | 1000 | 0.021 | 20.966 | -1.998, 0.09, 0.571 | 0.241, 0.242, 0.452 |
| Pes | 1000 | 0.07 | 69.863 | -2.112,0.681,0.72 | - |
| Hind limb | 1000 | 1.103 | 976.276 | -1.382, 2.19, 0.398 | - |
|  |  |  |  |  |  |
| HAT | 822.88 | 6.018 | 4952.12 | -0.341, 3.081, 0 | 1250.244, 21001.73, 21781.5 |
| Whole Body | 866.32 | 7.97 | 6904.67 | -0.636, 2.830, 0 | - |

Table S4. Results for the Plus 15% best estimate model of *Tyrannosaurus rex* MOR 555.

| Segment | Net Density (kg m-3) | Volume (m3) | Mass (kg) | CM (x,y,z) (m) | Ixx Iyy Izz (kg m2) |
| --- | --- | --- | --- | --- | --- |
| Head | 984.26 | 0.661 | 650.596 | 3.884, 3.062, 0 | 230.681, 6039.22, 6209.96 |
| Air sacs | 0 |  | 0 | - | - |
|  |  |  |  |  |  |
| Neck | 951.77 | 0.607 | 577.727 | 2.799, 3.190, 0 | 254.048, 2337.68, 2572.06 |
| Pharyngeal cavity | 0 | 0.029 | 0 | - | - |
|  |  |  |  |  |  |
| Thorax | 804.21 | 2.29 | 1841.65 | 1.431, 2.638, 0 | 664.957, 1073.58, 1561.97 |
| Lungs | 0 | 0.449 | 0 | - | - |
|  |  |  |  |  |  |
| Sacrum | 1000 | 0.679 | 659.477 | 0.214, 2.26, 0 | 312.459, 347.888, 649.698 |
|  |  |  |  |  |  |
| Tail | 1000 | 1.421 | 1420.774 | -1.848, 2.178, 0 | 288.893, 12083.3, 12334.3 |
|  |  |  |  |  |  |
| Arm | 1000 | 0.009 | 8.845 | 1.89, 2.097, 0.277 | 3.31989, 10.6317, 12.5619 |
| Digit I | 1000 | 0.0003 | 0.355 | 2.16, 1.922, 0.202 | 0.189, 0.641, 0.800 |
| Digit II | 1000 | 0.001 | 0.822 | 2.172, 1.846, 0 | 0.530, 1.512, 1.974 |
| Total Fore limb | 1000 | 0.010 | 10.022 | 1.923, 0.294, 0.268 | 4.03849, 12.7845, 15.336 |
|  |  |  |  |  |  |
| Thigh | 1000 | 0.885 | 884.663 | -0.106, 2.328, 0.413 | 210.547, 104.1, 263.14 |
| Shank | 1000 | 0.285 | 284.567 | -0.596, 1.107, 0.432 | 20.6676, 29.2981, 39.6174 |
| Metatarsus | 1000 | 0.057 | 56.684 | -0.866, 0.382, 0.423 | 1.61698, 1.39136, 1.72567 |
| Digit II | 1000 | 0.007 | 7.128 | -0.477, 0.077, 0.25 | 0.038, 0.166, 0.155 |
| Digit III | 1000 | 0.01 | 9.765 | -0.432, 0.09, 0.451 | 0.036, 0.336, 0.331 |
| Digit IV | 1000 | 0.009 | 8.814 | -0.489, 0.091, 0.614 | 0.055, 0.227, 0.203 |
| Pes | 1000 | 0.026 | 25.707 | -0.464, 0.086, 0.449 | - |
| Hind limb | 1000 | 0.971 | 1251.62 | -0.210267, 1.78645, 0.418556 | - |
|  |  |  |  |  |  |
| HAT | 882.45 | 5.881 | 5189.96 | 0.832, 2.622, 0 | 1759.118, 21907.18, 23358.74 |
| Whole Body | 918.3 | 8.384 | 7699.53 | 0.492, 2.471, 0 | - |

Table S5. Results for the Plus 7.5% best estimate model of *Tyrannosaurus rex* MOR 555.

| Segment | Net Density (kg m-3) | Volume (m3) | Mass (kg) | CM (x,y,z) (m) | Ixx Iyy Izz (kg m2) |
| --- | --- | --- | --- | --- | --- |
| Head | 984.26 | 0.661 | 650.596 | 3.883, 3.062, 0 | 232.473, 5951.82, 6124.36 |
| Air sacs | 0 |  | 0 | - | - |
|  |  |  |  |  |  |
| Neck | 946.48 | 0.538 | 509.208 | 2.794, 3.157, 0 | 201.35, 2008.01, 2195.07 |
| Pharyngeal cavity | 0 | 0.029 | 0 | - | - |
|  |  |  |  |  |  |
| Thorax | 773.45 | 1.978 | 1529.89 | 1.394, 2.625, 0 | 481.96, 765.801, 1153.23 |
| Lungs | 0 | 0.449 | 0 | - | - |
|  |  |  |  |  |  |
| Sacrum | 1000 | 0.672 | 672.246 | 0.216, 2.271, 0 | 295.989, 361.337, 646.32 |
|  |  |  |  |  |  |
| Tail | 1000 | 1.25 | 1250 | -1.832, 2.188, 0 | 228.258, 10672.8, 10871.8 |
|  |  |  |  |  |  |
| Arm | 1000 | 0.009 | 8.845 | 1.89, 2.097, 0.277 | 3.2912, 10.2188, 12.1203 |
| Digit I | 1000 | 0.0003 | 0.355 | 2.16, 1.922, 0.202 | 0.187, 0.620, 0.778 |
| Digit II | 1000 | 0.001 | 0.822 | 2.172, 1.846, 0 | 0.526, 1.463, 1.921 |
| Total Fore limb | 1000 | 0.010 | 10.022 | 1.923, 0.294, 0.268 | 4.004, 12.302, 14.819 |
|  |  |  |  |  |  |
| Thigh | 1000 | 0.836 | 836.404 | -0.107, 2.325, 0.39 | 197.544, 97.4931, 250.569 |
| Shank | 1000 | 0.247 | 246.61 | -0.591, 1.11, 0.429 | 16.8463, 24.0017, 33.011 |
| Metatarsus | 1000 | 0.05 | 50.285 | -0.863, 0.388, 0.42 | 1.37835, 1.13679, 1.51686 |
| Digit II | 1000 | 0.007 | 7.128 | -0.477, 0.077, 0.25 | 0.038, 0.166, 0.155 |
| Digit III | 1000 | 0.01 | 9.765 | -0.432, 0.09, 0.451 | 0.036, 0.336, 0.331 |
| Digit IV | 1000 | 0.009 | 8.814 | -0.489, 0.091, 0.614 | 0.054, 0.227, 0.203 |
| Pes | 1000 | 0.026 | 25.707 | -0.464, 0.086, 0.449 | - |
| Hind limb | 1000 |  | 1159.01 | -0.208, 1.810, 0.401 | - |
|  |  |  |  |  |  |
| HAT | 870.13 | 5.323 | 4631.71 | 0.855, 2.619, 0 | 1448.034, 19784.4, 21020.42 |
| Whole Body | 910.36 | 7.641 | 6956.06 | 0.499, 2.349, 0 | - |

Table S6. Results for the Minus 7.5% best estimate model of *Tyrannosaurus rex* MOR 555.

| Segment | Net Density (kg m-3) | Volume (m3) | Mass (kg) | CM (x,y,z) (m) | Ixx Iyy Izz (kg m2) |
| --- | --- | --- | --- | --- | --- |
| Head | 984.26 | 0.661 | 650.596 | 3.883, 3.063, 0 | 227.171, 5551.07, 5718.31 |
| Air sacs | 0 |  | 0 | - | - |
|  |  |  |  |  |  |
| Neck | 931.82 | 0.425 | 396.024 | 2.784, 3.096, 0 | 121.262, 1393.27, 1507.48 |
| Pharyngeal cavity | 0 | 0.029 | 0 | - | - |
|  |  |  |  |  |  |
| Thorax | 718.6 | 1.594 | 1145.45 | 1.361, 2.646, 0 | 277.436, 408.321, 669.798 |
| Lungs | 0 | 0.449 | 0 | - | - |
|  |  |  |  |  |  |
| Sacrum | 1000 | 0.669 | 669.434 | 0.23, 2.279, 0 | 290.478, 441.153, 719.847 |
|  |  |  |  |  |  |
| Tail | 1000 | 0.941 | 940.792 | -1.803, 2.369, 0 | 147.244, 8423.74, 8554.46 |
|  |  |  |  |  |  |
| Arm | 1000 | 0.009 | 8.845 | 1.89, 2.097, 0.277 | 3.37721, 8.40197, 10.3895 |
| Digit I | 1000 | 0.0003 | 0.355 | 2.16, 1.922, 0.202 | 0.191845, 0.527091, 0.689694, 0.301011, 0.050658, -0.0861284 |
| Digit II | 1000 | 0.001 | 0.822 | 2.172, 1.846, 0 | 0.538, 1.246, 1.716 |
| Total Fore limb | 1000 | 0.010 | 10.022 | 1.923, 0.294, 0.268 | 4.107, 10.175, 12.795 |
|  |  |  |  |  |  |
| Thigh | 1000 | 0.63 | 629.98 | -0.116, 2.305, 0.349 | 134.877, 68.0129, 177.073 |
| Shank | 1000 | 0.181 | 181.07 | -0.576, 1.119, 0.423 | 10.990, 16.075, 22.792 |
| Metatarsus | 1000 | 0.039 | 38.872 | -0.855, 0.396, 0.413 | 1.041, 0.726, 1.186 |
| Digit II | 1000 | 0.007 | 7.128 | -0.477, 0.077, 0.25 | 0.038, 0.166, 0.155 |
| Digit III | 1000 | 0.01 | 9.765 | -0.432, 0.09, 0.451 | 0.036, 0.336, 0.331 |
| Digit IV | 1000 | 0.009 | 8.814 | -0.489, 0.091, 0.614 | 0.055, 0.227, 0.203 |
| Pes | 1000 | 0.026 | 25.707 | -0.464, 0.086, 0.449 | - |
| Hind limb | 1000 |  | 875.629 | -0.221, 1.789, 0.370 | - |
|  |  |  |  |  |  |
| HAT | 846.91 | 4.513 | 3822.1 | 0.959, 2.628, 0 | 1071.807, 16237.88, 17195.49 |
| Whole Body | 890.76 | 6.264 | 5579.7 | 0.587, 2.364, 0 | - |

Table S7. Results for the Plus 15% best estimate model of *Acrocanthosaurus atokensis* NCSM 14345.

| Segment | Net Density (kg m-3) | Volume (m3) | Mass (kg) | CM (x,y,z) (m) | Ixx Iyy Izz (kg m2) |
| --- | --- | --- | --- | --- | --- |
| Head | 981.64 | 0.405 | 397.566 | 3.437, 2.138, 0 | 43.4817, 4678.81, 4708.3, |
| Air sacs | 0 | 0.007 | 0 | - | - |
|  |  |  |  |  |  |
| Neck | 926.31 | 0.407 | 377.011 | 2.341, 2.18, 0 | 56.0413, 2067.17, 2107.96 |
| Pharyngeal cavity | 0 | 0.03 | 0 | - | - |
|  |  |  |  |  |  |
| Thorax | 790.99 | 3.214 | 2542.25 | 1.051, 2.127, 0 | 961.436, 3614.11, 4055.89 |
| Lungs | 0 | 0.58 | 0 | - | - |
|  |  |  |  |  |  |
| Sacrum | 1000 | 0.831 | 831.492 | -0.565,  2.435, 0 | 256.657, 462.896, 704.375 |
|  |  |  |  |  |  |
| Tail | 1000 | 1.57 | 1570.46 | -1.956, 2.471, 0 | 295.04, 14279.8, 14441.9 |
|  |  |  |  |  |  |
| Arm | 1000 | 0.01 | 10.024 | 1.694, 1.252, -0.414 | 12.341, 29.708, 38.475 |
| Digit I | 1000 | 0.0005 | 0.491 | 1.721, 0.919, -0.585 | 1.06, 1.579, 2.302 |
| Digit II | 1000 | 0.001 | 1.207 | 1.814, 0.852, -0.531 | 2.761, 4.199, 6.278 |
| Digit IV | 1000 | 0.001 | 0.639 | 1.815, 0.898, -0.422 | 1.311, 2.159, 3.242 |
| Forelimb | 1000 | 0.012 | 12.361 | 1.713,1.181,0.433 | 17.865, 33.135, 46.178 |
|  |  |  |  |  |  |
| Thigh | 1000 | 0.77 | 770.088 | -0.478, 2.048, 0.382 | 109.384, 122.51, 187.825 |
| Shank | 1000 | 0.185 | 185.173 | -0.259, 0.934, 0.308 | 15.431, 8.203, 18.217 |
| Metatarsus | 1000 | 0.041 | 40.864 | -0.289, 0.238, 0.319 | 0.903, 0.892, 0.947 |
| Digit II | 1000 | 0.002 | 2.257 | 0.023,0.067,0.185 | 0.003, 0.004, 0.004 |
| Digit III | 1000 | 0.003 | 3.647 | 0.105,0.026,0.341 | 0.006, 0.008, 0.008 |
| Digit IV | 1000 | 0.002 | 1.767 | 0.019,0.071,0.491 | 0.002, 0.002, 0.003 |
| Pes | 1000 | 0.007 | 7.671 | 0.062,0.049,0.33 | - |
| Hind limb | 1000 | 1.003 | 1003.89 | 0.426, 1.754, 0.366 | - |
|  |  |  |  |  |  |
| HAT | 890.23 | 6.452 | 5743.5 | 0.026, 2.266, 0 | 1647.604, 25178.05, 26119.1 |
| Whole Body | 917.1 | 8.451 | 7750.61 | -0.091, 2.134, 0 | - |

Table S8. Results for the Plus 7.5% best estimate model of *Acrocanthosaurus atokensis* NCSM 14345.

| Segment | Net Density (kg m-3) | Volume (m3) | Mass (kg) | CM (x,y,z) (m) | Ixx Iyy Izz (kg m2) |
| --- | --- | --- | --- | --- | --- |
| Head | 981.64 | 0.405 | 397.566 | 3.437, 2.138, 0 | 44.3345, 2975.41, 3005.75 |
| Air sacs | 0 | 0.007 | 0 | - | - |
|  |  |  |  |  |  |
| Neck | 921.88 | 0.379 | 349.394 | 2.334, 2.177, 0 | 50.8371, 947.927, 984.933 |
| Pharyngeal cavity | 0 | 0.03 | 0 | - | - |
|  |  |  |  |  |  |
| Thorax | 748.97 | 2.9 | 2172.85 | 1.051, 2.149, 0 | 699.608, 1036.51, 1343.69 |
| Lungs | 0 | 0.58 | 0 | - | - |
|  |  |  |  |  |  |
| Sacrum | 1000 | 0.81 | 809.987 | -0.547,  2.423, 0 | 219.38, 1480.05, 1683.55 |
|  |  |  |  |  |  |
| Tail | 1000 | 1.335 | 1334.563 | -1.956, 2.474, 0 | 175.63, 3098.95, 3234.3 |
|  |  |  |  |  |  |
| Arm | 1000 | 0.01 | 10.024 | 1.694, 1.252, -0.414 | 12.506, 11.219, 20.151 |
| Digit I | 1000 | 0.0004 | 0.491 | 1.721, 0.919, -0.585 | 1.071, 0.654, 1.389 |
| Digit II | 1000 | 0.001 | 1.207 | 1.814, 0.852, -0.531 | 2.789, 1.770, 3.877 |
| Digit IV | 1000 | 0.001 | 0.639 | 1.815, 0.898, -0.422 | 1.325, 0.872, 1.969 |
| Forelimb | 1000 | 0.012 | 12.361 | 1.713,1.181,0.433 | 17.437, 14.298, 26.805 |
|  |  |  |  |  |  |
| Thigh | 1000 | 0.689 | 688.6 | -0.505, 2.053, 0.385 | 91.970, 99.876, 160.773 |
| Shank | 1000 | 0.162 | 161.884 | -0.258, 0.933, 0.308 | 13.134, 6.478, 15.377 |
| Metatarsus | 1000 | 0.037 | 37.436 | -0.281, 0.234, 0.319 | 0.809, 0.773, 0.881 |
| Digit II | 1000 | 0.002 | 2.257 | 0.023, 0.067, 0.185 | 0.0033, 0.004, 0.004 |
| Digit III | 1000 | 0.003 | 3.647 | 0.105, 0.026, 0.341 | 0.006, 0.008, 0.008 |
| Digit IV | 1000 | 0.002 | 1.767 | 0.019, 0.071, 0.491 | 0.002, 0.002, 0.003 |
| Pes | 1000 | 0.007 | 7.671 | 0.062, 0.049, 0.33 | - |
| Hind limb | 1000 | 0.895 | 895.69 | -0.446, 1.758, 0.368 | - |
|  |  |  |  |  |  |
| HAT | 869.93 | 5.854 | 5089.08 | 0.726, 2.27, 0 | 1224.917, 9567.658, 10306.4 |
| Whole Body | 921.94 | 7.462 | 6879.78 | 0.420966 2.13985 0 |  |

Table S9. Results for the Minus 7.5% best estimate model of *Acrocanthosaurus atokensis* NCSM 14345.

| Segment | Net Density (kg m-3) | Volume (m3) | Mass (kg) | CM (x,y,z) (m) | Ixx Iyy Izz (kg m2) |
| --- | --- | --- | --- | --- | --- |
| Head | 981.64 | 0.405 | 397.566 | 3.437, 2.138, 0 | 45.098, 4347.03, 4378.14 |
| Air sacs | 0 | 0.007 | 0 | - | - |
|  |  |  |  |  |  |
| Neck | 894.06 | 0.28 | 250.337 | 2.345, 2.210, 0 | 28.179, 1234.44, 1254.97 |
| Pharyngeal cavity | 0 | 0.03 | 0 | - | - |
|  |  |  |  |  |  |
| Thorax | 706.43 | 2.319 | 1638.21 | 1.053, 2.180, 0 | 377.962, 1966.48, 2098.81 |
| Lungs | 0 | 0.58 | 0 | - | - |
|  |  |  |  |  |  |
| Sacrum | 1000 | 0.745 | 744.899 | -0.554,  2.41, 0 | 186.516, 514.659, 688.087 |
|  |  |  |  |  |  |
| Tail | 1000 | 0.975 | 975.222 | -2.768, 2.457, 0 | 95.147, 9584.22, 9657.58 |
|  |  |  |  |  |  |
| Arm | 1000 | 0.01 | 10.024 | 1.694, 1.252, -0.414 | 12.6474, 25.6979, 34.7703 |
| Digit I | 1000 | 0.0005 | 0.491 | 1.721, 0.919, -0.585 | 1.080, 1.379, 2.122 |
| Digit II | 1000 | 0.001 | 1.207 | 1.814, 0.852, -0.531 | 2.812, 3.680, 5.810 |
| Digit IV | 1000 | 0.0006 | 0.639 | 1.815, 0.898, -0.422 | 1.337, 1.884, 2.993 |
| Forelimb | 1000 | 0.012 | 12.361 | 1.713, 1.181, 0.433 | 17.877, 32.641, 45.696 |
|  |  |  |  |  |  |
| Thigh | 1000 | 0.611 | 610.558 | -0.505, 2.057, 0.347 | 80.863, 88.150, 143.494 |
| Shank | 1000 | 0.123 | 122.933 | -0.253, 0.938, 0.308 | 9.678, 3.925, 11.166 |
| Metatarsus | 1000 | 0.028 | 28.379 | -0.273, 0.238, 0.321 | 0.546, 0.508, 0.651 |
| Digit II | 1000 | 0.002 | 2.257 | 0.023, 0.067, 0.185 | 0.003, 0.004, 0.004 |
| Digit III | 1000 | 0.003 | 3.647 | 0.105, 0.026, 0.341 | 0.006, 0.008, 0.008 |
| Digit IV | 1000 | 0.002 | 1.767 | 0.019, 0.071, 0.491 | 0.002, 0.002, 0.003 |
| Pes | 1000 | 0.007 | 7.671 | 0.062,0.049,0.33 | - |
| Hind limb | 1000 | 0.770 | 769.64 | -0.451, 1.792, 0.340 | - |
|  |  |  |  |  |  |
| HAT | 848.98 | 4.748675 | 4030.96 | 0.151, 2.281, 0 | 975.996, 20120.7, 20677.1 |
| Whole Body | 914.21 | 6.092207 | 5569.56 | -0.015, 2.146, 0 | - |

Table S10. Results for the Plus 15% best estimate model of *Struthiomimus sedens* BHI 1266.

| Segment | Net Density (kg m-3) | Volume (m3) | Mass (kg) | CM (x,y,z) (m) | Ixx Iyy Izz (kg m2) |
| --- | --- | --- | --- | --- | --- |
| Head | 974.01 | 0.0016 | 1.649 | 1.894, 2.302, 0 | 0.649, 4.078, 4.724 |
| Air sacs | 0 |  | 0 | - | - |
|  |  |  |  |  |  |
| Neck | 930.63 | 0.027 | 25.127 | 1.331, 1.903, 0 | 2.524, 26.388, 28.956 |
| Pharyngeal cavity | 0 | 0.002 | 0 | - | - |
|  |  |  |  |  |  |
| Thorax | 854.14 | 0.183 | 156.308 | 0.649, 1.654, 0 | 9.362, 24.826, 30.577 |
| Lungs | 0 |  | 0 | - | - |
|  |  |  |  |  |  |
| Sacrum | 1000 | 0.087 | 87.097 | -0.019, 1.627, 0 | 3.484, 14.635, 17.510 |
|  |  |  |  |  |  |
| Tail | 1000 | 0.049 | 49.185 | -0.858, 1.818, 0 | 5.056, 78.521, 83.285 |
|  |  |  |  |  |  |
| Arm | 1000 | 0.008 | 8.042 | 0.887, 1.384, 0.227 | 1.352, 3.032, 3.504 |
| Digit I | 1000 | 0.0001 | 0.157 | 0.887, 1.384, 0.227 | 0.100, 0.101, 0.166 |
| Digit II | 1000 | 0.0001 | 0.141 | 1.053, 0.953, 0.335 | 0.104, 0.084, 0.155 |
| Digit III | 1000 | 0.0001 | 0.132 | 0.981, 0.904, 0.326 | 0.049, 0.039, 0.073 |
| Fore limb | 1000 | 0.008 | 8.472 | 0.894,1.36,0.232 | 1.606, 3.256, 3.897 |
|  |  |  |  |  |  |
| Thigh | 1000 | 0.058 | 57.575 | 0.064, 1.607, 0.191 | 2.233, 1.666, 3.537 |
| Shank | 1000 | 0.028 | 27.984 | 0.194, 0.973, 0.15 | 1.186, 0.297, 1.344 |
| Metatarsus | 1000 | 0.006 | 6.015 | 0.216, 0.397, 0.11 | 0.067, 0.046, 0.086 |
| Digit II | 1000 | 0.001 | 0.656 | 0.444, 0.159, 0.051 | 0.001, 0.003, 0.004 |
| Digit III | 1000 | 0.001 | 0.807 | 0.51, 0.157, 0.106 | 0.003, 0.006, 0.009 |
| Digit IV | 1000 | 0.001 | 0.799 | 0.441, 0.157, 0.142 | 0.002, 0.004, 0.005 |
| Pes | 1000 | 0.003 | 2.262 | 0.466,0.158,0.103 | - |
| Hind limb | 1000 | 0.095 | 93.836 | 0.093, 1.160, 0.171 | - |
|  |  |  |  |  |  |
| HAT | 924.36 | 0.364 | 336.467 | 0.324, 1.677, 0 | 24.287, 154.96, 172.847 |
| Whole Body | 946.55 | 0.554 | 524.139 | 0.241, 1.492, 0 | - |

Table S11. Results for the Plus 7.5% best estimate model of *Struthiomimus sedens* BHI 1266.

| Segment | Net Density (kg m-3) | Volume (m3) | Mass (kg) | CM (x,y,z) (m) | Ixx Iyy Izz (kg m2) |
| --- | --- | --- | --- | --- | --- |
| Head | 974.01 | 0.0016 | 1.649 | 1.894, 2.302, 0 | 0.645, 4.124, 4.767 |
| Air sacs | 0 |  | 0 | - | - |
|  |  |  |  |  |  |
| Neck | 920.04 | 0.023 | 21.161 | 1.338, 1.913, 0 | 2.172, 22.893, 25.152 |
| Pharyngeal cavity | 0 | 0.002 | 0 | - | - |
|  |  |  |  |  |  |
| Thorax | 830.5 | 0.161 | 133.771 | 0.649, 1.657, 0 | 7.106, 21.870, 26.220 |
| Lungs | 0 |  | 0 | - | - |
|  |  |  |  |  |  |
| Sacrum | 1000 | 0.083 | 83.445 | -0.024, 1.637, 0 | 3.036, 13.594, 16.072 |
|  |  |  |  |  |  |
| Tail | 1000 | 0.043 | 42.91 | -0.855, 1.822, 0 | 4.335, 67.247, 71.359 |
|  |  |  |  |  |  |
| Arm | 1000 | 0.008 | 8.042 | 0.887, 1.384, 0.227 | 1.361, 3.113, 3.594 |
| Digit I | 1000 | 0.0001 | 0.157 | 0.887, 1.384, 0.227 | 0.100, 0.103, 0.168 |
| Digit II | 1000 | 0.0001 | 0.141 | 1.053, 0.953, 0.335 | 0.105, 0.09, 0.157 |
| Digit III | 1000 | 0.0001 | 0.132 | 0.981, 0.904, 0.326 | 0.049, 0.04, 0.074 |
| Fore limb | 1000 | 0.008 | 8.472 | 0.894,1.36,0.232 | 1.615, 3.342, 3.993 |
|  |  |  |  |  |  |
| Thigh | 1000 | 0.052 | 51.869 | 0.053, 1.612, 0.184 | 2.071, 1.394, 3.186 |
| Shank | 1000 | 0.024 | 23.51 | 0.191, 0.967, 0.15 | 0.993, 0.218, 1.110 |
| Metatarsus | 1000 | 0.005 | 5.255 | 0.214, 0.395, 0.112 | 0.057, 0.041, 0.077 |
| Digit II | 1000 | 0.001 | 0.656 | 0.444, 0.159, 0.051 | 0.001, 0.003, 0.004 |
| Digit III | 1000 | 0.001 | 0.807 | 0.51, 0.157, 0.106 | 0.003, 0.006, 0.009 |
| Digit IV | 1000 | 0.001 | 0.799 | 0.441, 0.157, 0.142 | 0.002, 0.004, 0.005 |
| Pes | 1000 | 0.003 | 2.262 | 0.466,0.158,0.103 | - |
| Hind limb | 1000 | 0.084 | 82.896 | 0.087, 1.175, 0.168 | - |
|  |  |  |  |  |  |
| HAT | 914.75 | 0.328 | 300.037 | 0.315, 1.678, 0 | 20.525, 136.411, 151.557 |
| Whole Body | 939.29 | 0.496 | 465.829 | 0.234, 1.499, 0 | - |

Table S12. Results for the Minus 7.5% best estimate model of *Struthiomimus sedens* BHI 1266.

| Segment | Net Density (kg m-3) | Volume (m3) | Mass (kg) | CM (x,y,z) (m) | Ixx Iyy Izz (kg m2) |
| --- | --- | --- | --- | --- | --- |
| Head | 974.01 | 0.0016 | 1.649 | 1.894, 2.302, 0 | 0.655, 4.128, 4.781 |
| Air sacs | 0 |  | 0 | - | - |
|  |  |  |  |  |  |
| Neck | 903.53 | 0.017 | 15.136 | 1.342, 1.924, 0 | 1.995, 16.659, 17.745 |
| Pharyngeal cavity | 0 | 0.002 | 0 | - | - |
|  |  |  |  |  |  |
| Thorax | 794.76 | 0.133 | 105.703 | 0.653, 1.660, 0 | 4.812, 17.494, 20.591 |
| Lungs | 0 |  | 0 | - | - |
|  |  |  |  |  |  |
| Sacrum | 1000 | 0.08 | 80.052 | -0.018, 1.641, 0 | 2.707, 12.519, 14.702 |
|  |  |  |  |  |  |
| Tail | 1000 | 0.032 | 31.601 | -0.853, 1.832, 0 | 3.265, 49.407, 52.550 |
|  |  |  |  |  |  |
| Arm | 1000 | 0.008 | 8.042 | 0.887, 1.384, 0.227 | 1.338, 3.120, 3.578 |
| Digit I | 1000 | 0.0001 | 0.157 | 0.887, 1.384, 0.227 | 0.099, 0.103, 0.167 |
| Digit II | 1000 | 0.0001 | 0.141 | 1.053, 0.953, 0.335 | 0.104, 0.086, 0.156 |
| Digit III | 1000 | 0.0001 | 0.132 | 0.981, 0.904, 0.326 | 0.049, 0.040, 0.074 |
| Fore limb | 1000 | 0.008 | 8.472 | 0.894,1.36,0.232 | 1.590, 3.349, 3.975 |
|  |  |  |  |  |  |
| Thigh | 1000 | 0.042 | 41.81 | 0.061, 1.617, 0.173 | 1.633, 1.008, 2.436 |
| Shank | 1000 | 0.017 | 17.02 | 0.189, 0.983, 0.152 | 0.726, 0.127, 0.798 |
| Metatarsus | 1000 | 0.004 | 4.009 | 0.222, 0.4, 0.116 | 0.047, 0.031, 0.066 |
| Digit II | 1000 | 0.001 | 0.656 | 0.444, 0.159, 0.051 | 0.001, 0.003, 0.004 |
| Digit III | 1000 | 0.001 | 0.807 | 0.51, 0.157, 0.106 | 0.003, 0.006, 0.009 |
| Digit IV | 1000 | 0.001 | 0.799 | 0.441, 0.157, 0.142 | 0.002, 0.004, 0.005 |
| Pes | 1000 | 0.003 | 2.262 | 0.466, 0.158, 0.103 | - |
| Hind limb | 1000 | 0.066 | 65.101 | 0.0938, 1.197, 0.162 | - |
|  |  |  |  |  |  |
| HAT | 897.29 | 0.28 | 251.242 | 0.314, 1.674, 0 | 16.615, 106.906, 118.318 |
| Whole Body | 925.83 | 0.412 | 381.444 | 0.239, 1.511, 0 | - |

Table S13. Results for the Plus 15% best estimate model of *Edmontosaurus annectens* BHI 126950.

| Segment | Net Density (kg m-3) | Volume (m3) | Mass (kg) | CM (x,y,z) (m) | Ixx Iyy Izz (kg m2) |
| --- | --- | --- | --- | --- | --- |
| Head | 962.4 | 0.028 | 27.17 | 1.082, 1.623, 0 | 6.217, 32.468, 38.466 |
| Air sacs | 0 | 0.006 | 0 | - | - |
|  |  |  |  |  |  |
| Neck | 1000 | 0.032 | 32.178 | 0.837, 1.202, 0 | 0.938, 27.506, 28.161 |
|  |  |  |  |  |  |
| Thorax | 814.63 | 0.334 | 272.085 | 0.228, 1.026, 0 | 29.699, 32.160, 48.910 |
| Lungs | 0 | 0.062 | 0 | - | - |
|  |  |  |  |  |  |
| Sacrum | 1000 | 0.196 | 196.416 | -0.596, 1.242, 0 | 12.508, 87.150, 97.297 |
|  |  |  |  |  |  |
| Tail | 1000 | 0.095 | 94.58 | -1.556, 1.346, 0 | 5.931, 14.093, 19.457 |
|  |  |  |  |  |  |
| Fore limb | 1000 | 0.011 | 11.031 | 0.618, 0.578, 0.129 | 4.231, 4.578, 8.398 |
|  |  |  |  |  |  |
| Thigh | 1000 | 0.129 | 129.235 | -0.597, 1.181, 0.213 | 4.726, 7.919, 10.553 |
| Shank | 1000 | 0.03 | 30.259 | -0.341, 0.576, 0.246 | 0.902, 0.411, 1.090 |
| Metatarsus | 1000 | 0.006 | 5.732 | -0.389, 0.2, 0.21 | 0.033, 0.035, 0.0265 |
| Digit II | 1000 | 0.001 | 1.158 | -0.299, 0.084, 0.051 | 0.003, 0.004, 0.003 |
| Digit III | 1000 | 0.001 | 1.457 | -0.296, 0.095, 0.184 | 0.003, 0.007, 0.007 |
| Digit IV | 1000 | 0.001 | 0.925 | -0.296, 0.061, 0.281 | 0.002, 0.003, 0.003 |
| Pes | 1000 | 0.004 | 4.473 | -0.27, 0.077, 0.171 | - |
| Hind limb | 1000 | 0.169 | 169.699 | -0.505, 0.958, 0.218 | - |
|  |  |  |  |  |  |
| HAT | 911.56 | 0.707 | 644.491 | 0.0002, 1.156, 0 | 63.989, 198.190, 244.798 |
| Whole Body | 941.52 | 1.045 | 983.889 | -0.174, 1.088, 0 | - |

Table S14. Results for the Plus 7.5% best estimate model of *Edmontosaurus annectens* BHI 126950.

| Segment | Net Density (kg m-3) | Volume (m3) | Mass (kg) | CM (x,y,z) (m) | Ixx Iyy Izz (kg m2) |
| --- | --- | --- | --- | --- | --- |
| Head | 962.4 | 0.028 | 27.17 | 1.082, 1.623, 0 | 5.768, 32.728, 38.277 |
| Air sacs | 0 | 0.006 | 0 | - | - |
|  |  |  |  |  |  |
| Neck | 1000 | 0.027 | 25.36 | 0.837, 1.202, 0 | 0.938, 27.506, 28.161 |
|  |  |  |  |  |  |
| Thorax | 793.91 | 0.3 | 238.172 | 0.235, 1.049, 0 | 29.699, 32.160, 48.910 |
| Lungs | 0 | 0.062 | 0 | - | - |
|  |  |  |  |  |  |
| Sacrum | 1000 | 0.187 | 187.067 | -0.594, 1.257, 0 | 10.948, 81.265, 89.966 |
|  |  |  |  |  |  |
| Tail | 1000 | 0.083 | 82.836 | -1.559, 1.353, 0 | 4.574, 12.206, 16.344 |
|  |  |  |  |  |  |
| Fore limb | 1000 | 0.011 | 11.031 | 0.618, 0.578, 0.129 | 4.465, 4.638, 8.691 |
|  |  |  |  |  |  |
| Thigh | 1000 | 0.121 | 120.513 | -0.589, 1.182, 0.205 | 4.331, 7.236, 9.828 |
| Shank | 1000 | 0.026 | 26.409 | -0.343, 0.576, 0.242 | 0.776, 0.329, 0.935 |
| Metatarsus | 1000 | 0.005 | 5.216 | -0.392, 0.199, 0.206 | 0.033, 0.035, 0.026 |
| Digit II | 1000 | 0.001 | 1.158 | -0.299, 0.084, 0.051 | 0.028, 0.029, 0.024 |
| Digit III | 1000 | 0.001 | 1.457 | -0.296, 0.095, 0.184 | 0.003, 0.007, 0.007 |
| Digit IV | 1000 | 0.001 | 0.925 | -0.296, 0.061, 0.281 | 0.002, 0.003, 0.003 |
| Pes | 1000 | 0.004 | 4.473 | -0.27, 0.077, 0.171 | - |
| Hind limb | 1000 | 0.157 | 156.611 | -0.503, 0.966, 0.210 | - |
|  |  |  |  |  |  |
| HAT | 902.99 | 0.647 | 584.236 | -0.004, 1.174, 0 | 54.936, 183.743,  224.065 |
| Whole Body | 935.83 | 0.959 | 897.458 | -0.178, 1.101, 0 | - |

Table S15. Results for the Minus 7.5% best estimate model of *Edmontosaurus* BHI 126950.

| Segment | Net Density (kg m-3) | Volume (m3) | Mass (kg) | CM (x,y,z) (m) | Ixx Iyy Izz (kg m2) |
| --- | --- | --- | --- | --- | --- |
| Head | 962.4 | 0.028 | 27.17 | 1.082, 1.623, 0 | 4.878, 44.997, 49.656 |
| Air sacs | 0 | 0.006 | 0 | - | - |
|  |  |  |  |  |  |
| Neck | 1000 | 0.021 | 25.36 | 0.837, 1.202, 0 | 0.938, 27.506, 28.161 |
|  |  |  |  |  |  |
| Thorax | 732.81 | 0.231 | 169.28 | 0.249, 1.098,  0 | 13.923, 43.495, 51.560 |
| Lungs | 0 | 0.062 | 0 | - | - |
|  |  |  |  |  |  |
| Sacrum | 1000 | 0.173 | 172.552 | -0.589, 1.285, 0 | 8.327, 41.404, 47.595 |
|  |  |  |  |  |  |
| Tail | 1000 | 0.06 | 60.26 | -1.556, 1.366, 0 | 2.43366, 118.989, 121.189 |
|  |  |  |  |  |  |
| Fore limb | 1000 | 0.011 | 11.031 | 0.618, 0.578, 0.129 | 4.981, 7.663 12.233 |
|  |  |  |  |  |  |
| Thigh | 1000 | 0.107 | 106.938 | -0.6, 1.191, 0.19 | 3.695, 6.134, 8.538 |
| Shank | 1000 | 0.019 | 19.499 | -0.35, 0.574, 0.233 | 0.552, 0.206, 0.664 |
| Metatarsus | 1000 | 0.004 | 4.128 | -0.397, 0.197, 0.2 | 0.020, 0.019, 0.018 |
| Digit II | 1000 | 0.001 | 1.158 | -0.299, 0.084, 0.051 | 0.028, 0.029, 0.024 |
| Digit III | 1000 | 0.001 | 1.457 | -0.296, 0.095, 0.184 | 0.003, 0.007, 0.007 |
| Digit IV | 1000 | 0.001 | 0.925 | -0.296, 0.061, 0.281 | 0.002, 0.003, 0.003 |
| Pes | 1000 | 0.004 | 4.473 | -0.27, 0.077, 0.171 | - |
| Hind limb | 1000 | 0.135 | 135.038 | -0.521, 0.992, 0.196 | - |
|  |  |  |  |  |  |
| HAT | 883.48 | 0.535 | 472.66 | -0.195, 1.212, 0 | 40.286,  287.616,  318.421 |
| Whole Body | 924.95 | 0.803 | 742.736 | -0.314, 1.132, 0 | - |

Table S16. Results for the Plus 15% body/Minus 7.5% Tail model of *Tyrannosaurus* BHI 3033

| Segment | Net Density (kg m-3) | Volume (m3) | Mass (kg) | CM (x,y,z) (m) | Ixx Iyy Izz (kg m2) |
| --- | --- | --- | --- | --- | --- |
| Head | 990.6 | 0.685 | 678.561 | 2.553, 3.346, 0 | 154.783, 5023.23, 5124.9 |
| Air sacs | 0 | 0.01 |  |  |  |
|  |  |  |  |  |  |
| Neck | 926.17 | 0.472 | 437.151 | 1.701, 3.367, 0 | 111.041, 1531.87, 1602.92 |
| Pharyngeal cavity | 0 | 0.035 | - | - | - |
|  |  |  |  |  |  |
| Thorax | 689.41 | 4.72 | 3254.13 | 0.394, 2.949, 0 | 1520.51, 2321.09, 3051.83 |
| Lungs | - | 0.764 | - | - | - |
|  |  |  |  |  |  |
| Sacrum | 1000 | 1.062 | 1062.439 | -1.319, 3.004, 0 | 269.578, 1700.46, 1937.76 |
|  |  |  |  |  |  |
| Tail | 1000 | 0.942 | 942.361 | -3.526, 3.099, 0 | 106.922, 12112.6, 12198.2 |
|  |  |  |  |  |  |
| Arm | 1000 | 0.011 | 10.931 | 1.138, 2.398, 0.483 | 7.399, 20.745, 22.865 |
| Digit I | 1000 | 0.001 | 0.644 | 1.315, 2.115, 0.62 | 0.811, 1.628, 1.943 |
| Digit II | 1000 | 0.001 | 0.548 | 1.33, 2.197, 0.591 | 0.590, 1.390, 1.597 |
| Fore limb | 1000 | 0.012 | 12.123 | 1.156, 2.374, 0.495 | 8.800, 23.762, 26.405 |
|  |  |  |  |  |  |
| Thigh | 1000 | 0.744 | 743.937 | -1.323, 2.853, 0.408 | 183.542, 78.4534, 226.284 |
| Shank | 1000 | 0.215 | 214.664 | -1.887, 1.687, 0.357 | 13.856, 21.791, 30.382 |
| Metatarsus | 1000 | 0.074 | 73.812 | -2.431, 0.914, 0.298 | 3.380, 1.501, 3.258 |
| Digit II | 1000 | 0.023 | 22.332 | -1.897, 0.11, 0.29 | 0.257, 0.259, 0.499 |
| Digit III | 1000 | 0.027 | 26.564 | -1.811, 0.134, 0.457 | 0.308, 0.540, 0.781 |
| Digit IV | 1000 | 0.021 | 20.966 | -1.998, 0.09, 0.571 | 0.241, 0.242, 0.452 |
| Pes | 1000 | 0.07 | 69.863 | -2.112, 0.681, 0.72 | - |
| Hind limb | 1000 | 1.102 | 1102.276 | -1.373, 2.194, 0.410 | - |
|  |  |  |  |  |  |
| HAT | 888.95 | 7.196 | 6396.88 | -0.148, 3.050, 0 | 2180.44, 22736.86, 23968.5 |
| Whole Body | 915.05 | 9.4 | 8601.43 | -0.462, 2.830, 0 | - |

Table S17. Results for the Minus 7.5% body/Plus 15% Tail model of *Tyrannosaurus* BHI 3033

| Segment | Net Density (kg m-3) | Volume (m3) | Mass (kg) | CM (x,y,z) (m) | Ixx Iyy Izz (kg m2) |
| --- | --- | --- | --- | --- | --- |
| Head | 990.6 | 0.685 | 678.561 | 2.553, 3.346, 0 | 143.752, 7044.53, 7135.17 |
| Air sacs | - | 0.01 | - | - | - |
|  |  |  |  |  |  |
| Neck | 896.83 | 0.342 | 306.716 | 1.707, 3.373, 0 | 56.7751, 1735.53, 1774.28 |
| Pharyngeal cavity | 0 | 0.035 | - | - | - |
|  |  |  |  |  |  |
| Thorax | 719.33 | 2.722 | 1958.02 | 0.372, 2.982, 0 | 660.151, 2802.39, 3127.53 |
| Lungs | 0 | 0.764 | - | - | - |
|  |  |  |  |  |  |
| Sacrum | 1000 | 1.062 | 1062.439 | -1.319, 3.004, 0 | 273.068, 712.617, 953.411 |
|  |  |  |  |  |  |
| Tail | 1000 | 1.468 | 1467.751 | -0.751, 3.079, 0 | 220.031, 14355.4, 14523.7 |
|  |  |  |  |  |  |
| Arm | 1000 | 0.011 | 10.931 | 1.138, 2.398, 0.483 | 7.801, 37.783, 40.305 |
| Digit I | 1000 | 0.001 | 0.644 | 1.315, 2.115, 0.62 | 0.845, 2.747, 3.096 |
| Digit II | 1000 | 0.001 | 0.548 | 1.33, 2.197, 0.591 | 0.616, 2.350, 2.583 |
| Fore limb | 1000 | 0.012 | 12.123 | 1.156, 2.374, 0.495 | 9.262, 42.880, 45.984 |
|  |  |  |  |  |  |
| Thigh | 1000 | 0.744 | 743.937 | -1.323, 2.853, 0.408 | 183.542, 78.4534, 226.284 |
| Shank | 1000 | 0.215 | 214.664 | -1.887, 1.687, 0.357 | 13.856, 21.791, 30.382 |
| Metatarsus | 1000 | 0.074 | 73.812 | -2.431, 0.914, 0.298 | 3.380, 1.501, 3.258 |
| Digit II | 1000 | 0.023 | 22.332 | -1.897, 0.11, 0.29 | 0.257, 0.259, 0.499 |
| Digit III | 1000 | 0.027 | 26.564 | -1.811, 0.134, 0.457 | 0.308, 0.540, 0.781 |
| Digit IV | 1000 | 0.021 | 20.966 | -1.998, 0.09, 0.571 | 0.241, 0.242, 0.452 |
| Pes | 1000 | 0.07 | 69.863 | -2.112, 0.681, 0.72 | - |
| Hind limb | 1000 | 1.102 | 1102.276 | -1.373, 2.194, 0.410 | - |
|  |  |  |  |  |  |
| HAT | 873.03 | 6.295 | 5495.73 | -0.654, 3.077, 0 | 1372.302, 26736.18,  27606.08 |
| Whole Body | 906.02 | 8.499 | 7700.28 | -0.860, 2.824, 0 | - |

Table S18. Results for the Plus 15% body/Minus 7.5% Tail model of *Tyrannosaurus* MOR 555

| Segment | Net Density (kg m-3) | Volume (m3) | Mass (kg) | CM (x,y,z) (m) | Ixx Iyy Izz (kg m2) |
| --- | --- | --- | --- | --- | --- |
| Head | 984.26 | 0.661 | 650.596 | 3.883, 3.063, 0 | 211.279, 4964.13, 5115.48 |
| Air sacs | 0 | 0.011 | 0 | - | - |
|  |  |  |  |  |  |
| Neck | 951.77 | 0.607 | 577.727 | 2.799, 3.189, 0 | 231.799, 1733.32, 1945.45 |
| Pharyngeal cavity | 0 | 0.029 | 0 | - | - |
|  |  |  |  |  |  |
| Thorax | 804.21 | 2.29 | 1841.65 | 1.431, 2.638, 0 | 665.124, 592.215, 1080.78 |
| Lungs | 0 | 0.449 | 0 | - | - |
|  |  |  |  |  |  |
| Sacrum | 1000 | 0.659 | 659.477 | 0.222, 2.272, 0 | 303.713, 613.05, 906.248 |
|  |  |  |  |  |  |
| Tail | 1000 | 0.941 | 940.792 | -1.803, 2.369, 0 | 162.144, 9280.27, 9425.89 |
|  |  |  |  |  |  |
| Arm | 1000 | 0.009 | 8.845 | 1.89, 2.097, 0.277 | 3.656, 5.991, 8.258 |
| Digit I | 1000 | 0.0003 | 0.355 | 2.16, 1.922, 0.202 | 0.207, 0.4, 0.577 |
| Digit II | 1000 | 0.001 | 0.822 | 2.172, 1.846, 0 | 0.576, 0.948, 1.455 |
| Fore limb | 1000 | 0.010 | 10.022 | 1.923, 0.294, 0.268 | 4.439, 7.338, 10.290 |
|  |  |  |  |  |  |
| Thigh | 1000 | 0.689 | 688.552 | -0.114, 2.289, 0.363 | 149.268, 76.017, 194.264 |
| Shank | 1000 | 0.212 | 212.459 | -0.584, 1.114, 0.426 | 13.684, 19.713, 27.550 |
| Metatarsus | 1000 | 0.044 | 44.408 | -0.859, 0.392, 0.417 | 1.204, 0.906, 1.343 |
| Digit II | 1000 | 0.007 | 7.128 | -0.477, 0.077, 0.25 | 0.0375787, 0.165644, 0.155 |
| Digit III | 1000 | 0.01 | 9.765 | -0.432, 0.09, 0.451 | 0.036, 0.336, 0.331 |
| Digit IV | 1000 | 0.009 | 8.814 | -0.489, 0.091, 0.614 | 0.055, 0.227, 0.203 |
| Pes | 1000 | 0.026 | 25.707 | -0.464, 0.086, 0.449 | - |
| Hind limb | 1000 | 0.971 | 971.126 | -0.221, 1.76, 0.382 | - |
|  |  |  |  |  |  |
| HAT | 905.41 | 5.18 | 4690.04 | 1.119, 2.657, 0 | 1582.939,  17197.64, 18494.39 |
| Whole Body | 931.35 | 7.128 | 6638.63 | 0.726, 2.394, 0 |  |

Table S19. Results for the Minus 7.5% body/Plus 15% Tail model of *Tyrannosaurus* MOR 555

| Segment | Net Density (kg m-3) | Volume (m3) | Mass (kg) | CM (x,y,z) (m) | Ixx Iyy Izz (kg m2) |
| --- | --- | --- | --- | --- | --- |
| Head | 984.26 | 0.661 | 650.596 | 3.884, 3.063, 0 | 248.304, 6836.74, 7025.11 |
| Air sacs | 0 | 0.011 | 0 | - | - |
|  |  |  |  |  |  |
| Neck | 931.82 | 0.425 | 396.024 | 2.784, 3.096, 0 | 134.999, 1901.62, 2029.57 |
| Pharyngeal cavity | 0 | 0.029 | 0 | - | - |
|  |  |  |  |  |  |
| Thorax | 718.6 | 1.594 | 1145.45 | 1.361, 2.646, 0 | 280.373, 825.611, 1090.02 |
| Lungs | 0 | 0.449 | 0 | - | - |
|  |  |  |  |  |  |
| Sacrum | 1000 | 0.659 | 659.477 | 0.222, 2.272, 0 | 273.625, 195.185, 458.295 |
|  |  |  |  |  |  |
| Tail | 1000 | 1.421 | 1420.774 | -1.848, 2.178, 0 | 267.204, 10642.7, 10872.1 |
|  |  |  |  |  |  |
| Arm | 1000 | 0.009 | 8.845 | 1.89, 2.097, 0.277 | 3.054, 14.647, 16.310 |
| Digit I | 1000 | 0.0003 | 0.355 | 2.16, 1.922, 0.202 | 0.174, 0.840, 0.985 |
| Digit II | 1000 | 0.001 | 0.822 | 2.172, 1.846, 0 | 0.493, 1.976, 2.401 |
| Fore limb | 1000 | 0.010 | 10.022 | 1.923, 0.294, 0.268 | 3.7199, 17.4633, 19.696 |
|  |  |  |  |  |  |
| Thigh | 1000 | 0.689 | 688.552 | -0.114, 2.289, 0.363 | 149.268, 76.017, 194.264 |
| Shank | 1000 | 0.212 | 212.459 | -0.584, 1.114, 0.426 | 13.684, 19.713, 27.550 |
| Metatarsus | 1000 | 0.044 | 44.408 | -0.859, 0.392, 0.417 | 1.204, 0.906, 1.343 |
| Digit II | 1000 | 0.007 | 7.128 | -0.477, 0.077, 0.25 | 0.038, 0.166, 0.155 |
| Digit III | 1000 | 0.01 | 9.765 | -0.432, 0.09, 0.451 | 0.036, 0.336, 0.331 |
|  |  |  |  |  |  |
| HAT | 897.74 | 4.781 | 4292.13 | 0.636, 2.593, 0 | 1211.94,  20436.76,  21514.5 |
| Whole Body | 927.3 | 6.73 | 6240.72 | 0.368, 2.333, 0 |  |

Table S20. Results for the Plus 15% body/Minus 7.5% Tail model of *Acrocanthosaurus* NCSM 14345

| Segment | Net Density (kg m-3) | Volume (m3) | Mass (kg) | CM (x,y,z) (m) | Ixx Iyy Izz (kg m2) |
| --- | --- | --- | --- | --- | --- |
| Head | 981.64 | 0.405 | 397.566 | 3.437, 2.138, 0 | 40.5736, 3809.36, 3835.94 |
| Air sacs | 0 | 0.007 | 0 | - | - |
|  |  |  |  |  |  |
| Neck | 926.31 | 0.407 | 377.011 | 2.341, 2.184, 0 | 54.4236, 1521.48, 1560.66 |
| Pharyngeal cavity | 0 | 0.03 | 0 | - | - |
|  |  |  |  |  |  |
| Thorax | 819.61 | 3.214 | 2542.25 | 1.051, 2.127, 0 | 941.09, 2146.63, 2568.06 |
| Lungs | 0 | 0.58 | 0 | - | - |
|  |  |  |  |  |  |
| Sacrum | 1000 | 0.768 | 768.158 | -0.54,  2.41, 0 | 205.502, 783.674, 974.855 |
|  |  |  |  |  |  |
| Tail | 1000 | 0.975 | 975.222 | -2.768, 2.457, 0 | 113.662, 10838.9, 10930.7 |
|  |  |  |  |  |  |
| Arm | 1000 | 0.01 | 10.024 | 1.694, 1.252, -0.414 | 11.6891, 19.573, 27.687 |
| Digit I | 1000 | 0.0005 | 0.491 | 1.721, 0.919, -0.585 | 1.017, 1.073, 1.754 |
| Digit II | 1000 | 0.001 | 1.207 | 1.814, 0.852, -0.531 | 2.651, 2.881, 4.850 |
| Digit III | 1000 | 0.0006 | 0.639 | 1.815, 0.898, -0.422 | 1.255, 1.461, 2.487 |
| Forelimb | 1000 | 0.012 | 12.361 | 1.713, 1.181, 0.433 | 16.613, 24.988, 36.778 |
|  |  |  |  |  |  |
| Thigh | 1000 | 0.664 | 663.709 | -0.495, 2.067, 0.35 | 91.336, 97.4778, 159.109 |
| Shank | 1000 | 0.142 | 142.12 | -0.255, 0.936, 0.308 | 11.385, 5.102, 13.239 |
| Metatarsus | 1000 | 0.033 | 32.925 | -0.277, 0.236, 0.32 | 0.670, 0.633, 0.762 |
| Digit II | 1000 | 0.002 | 2.257 | 0.023, 0.067, 0.185 | 0.003, 0.004, 0.004 |
| Digit III | 1000 | 0.003 | 3.647 | 0.105, 0.026, 0.341 | 0.006, 0.008, 0.008 |
| Digit IV | 1000 | 0.002 | 1.767 | 0.019, 0.071, 0.491 | 0.002, 0.002, 0.003 |
| Pes | 1000 | 0.007 | 7.671 | 0.062, 0.049, 0.33 | - |
| Hind limb | 1000 | 0.847 | 846.524 | -0.441, 1.788, 0.342 | - |
|  |  |  |  |  |  |
| HAT | 891.78 | 5.702 | 5084.93 | 0.364, 2.234, 0 | 1388.473, 19149.99, 19943.78 |
| Whole Body | 916.59 | 7.394 | 6777.3 | 0.163, 2.122, 0 | - |

Table S21. Results for the Minus 7.5% body/Plus 15% Tail model of *Acrocanthosaurus* NCSM 14345

| Segment | Net Density (kg m-3) | Volume (m3) | Mass (kg) | CM (x,y,z) (m) | Ixx Iyy Izz (kg m2) |
| --- | --- | --- | --- | --- | --- |
| Head | 981.64 | 0.405 | 397.566 | 3.437, 2.138, 0 | 48.5967, 5379.39, 5414 |
| Air sacs | 0 | 0.007034873 | 0 | - | - |
|  |  |  |  |  |  |
| Neck | 894.06 | 0.28 | 250.337 | 2.345, 2.210, 0 | 29.3763, 1680.03, 1701.76 |
| Pharyngeal cavity | 0 | 0.03 | 0 | - | - |
|  |  |  |  |  |  |
| Thorax | 749.91 | 2.319 | 1638.21 | 1.053, 2.180, 0 | 388.489, 3299.74, 3442.6 |
| Lungs | 0 | 0.58 | 0 | - | - |
|  |  |  |  |  |  |
| Sacrum | 1000 | 0.768 | 768.158 | -0.54,  2.41, 0 | 189.416, 233.826, 408.921 |
|  |  |  |  |  |  |
| Tail | 1000 | 1.57 | 1570.46 | -1.956, 2.471, 0 | 270.304, 12192.7, 12330.1 |
|  |  |  |  |  |  |
| Arm | 1000 | 0.01 | 10.024 | 1.694, 1.252, -0.414 | 13.232, 38.663, 48.320 |
| Digit I | 1000 | 0.00049069 | 0.491 | 1.721, 0.919, -0.585 | 1.118, 2.024, 2.805 |
| Digit II | 1000 | 0.00120746 | 1.207 | 1.814, 0.852, -0.531 | 2.910, 5.350, 7.577 |
| Digit IV | 1000 | 0.00063918 | 0.639 | 1.815, 0.898, -0.422 | 1.387, 2.769, 3.927 |
| Forelimb | 1000 | 0.012337327 | 12.361 | 1.713, 1.181, 0.433 | 18.647, 48.805, 62.629 |
|  |  |  |  |  |  |
| Thigh | 1000 | 0.664 | 663.709 | -0.495, 2.067, 0.35 | 91.336, 97.4778, 159.109 |
| Shank | 1000 | 0.142 | 142.12 | -0.255, 0.936, 0.308 | 11.3851, 5.102, 13.239 |
| Metatarsus | 1000 | 0.033 | 32.925 | -0.277, 0.236, 0.32 | 0.670, 0.633, 0.762 |
| Digit II | 1000 | 0.002118381 | 2.257 | 0.023, 0.067, 0.185 | 0.003, 0.004, 0.004 |
| Digit III | 1000 | 0.00348997 | 3.647 | 0.105, 0.026, 0.341 | 0.006, 0.008, 0.008 |
| Digit IV | 1000 | 0.001676117 | 1.767 | 0.019, 0.071, 0.491 | 0.002, 0.002, 0.003 |
| Pes | 1000 | 0.007284468 | 7.671 | 0.062, 0.049, 0.33 | - |
| Hind limb | 1000 | 0.847 | 846.524 | -0.441, 1.788, 0.342 | - |
|  |  |  |  |  |  |
| HAT | 882.92 | 5.266 | 4649.46 | -0.223, 2.309, 0 | 963.4759, 22883.3, 23422.63 |
| Whole Body | 911.3 | 6.959 | 6341.82 | -0.281, 2.170, 0 | - |

Table S22. Results for the Plus 15% body/Minus 7.5% Tail model of *Struthiomimus* *sedens* BHI 1266

| Segment | Net Density (kg m-3) | Volume (m3) | Mass (kg) | CM (x,y,z) (m) | Ixx Iyy Izz (kg m2) |
| --- | --- | --- | --- | --- | --- |
| Head | 974.01 | 0.0016 | 1.649 | 1.894, 2.302, 0 | 0.654, 3.710, 4.362 |
| Air sacs | 0 | 0.004 | 0 | - | - |
|  |  |  |  |  |  |
| Neck | 930.63 | 0.027 | 25.127 | 1.331, 1.903, 0 | 2.553, 22.843, 25.440 |
| Pharyngeal cavity | 0 | 0.002 | 0 | - | - |
|  |  |  |  |  |  |
| Thorax | 854.54 | 0.183 | 156.308 | 0.649, 1.654, 0 | 9.345, 18.278, 24.012 |
| Lungs | 0 | 0.027 | 0 | - | - |
|  |  |  |  |  |  |
| Sacrum | 1000 | 0.082 | 81.723 | -0.019, 1.639, 0 | 2.822, 17.985, 20.258 |
|  |  |  |  |  |  |
| Tail | 1000 | 0.032 | 31.601 | -0.853, 1.832, 0 | 3.261, 55.694, 58.833 |
|  |  |  |  |  |  |
| Arm | 1000 | 0.008 | 8.042 | 0.887, 1.384, 0.227 | 1.340, 2.416, 2.876 |
| Digit I | 1000 | 0.0001 | 0.157 | 0.887, 1.384, 0.227 | 0.099, 0.085, 0.150 |
| Digit II | 1000 | 0.0001 | 0.141 | 1.053, 0.953, 0.335 | 0.104, 0.070, 0.141 |
| Digit III | 1000 | 0.000132 | 0.132 | 0.981, 0.904, 0.326 | 0.049, 0.032, 0.066 |
| Fore limb | 1000 | 0.008332 | 8.472 | 0.894, 1.36, 0.232 | 1.592, 2.604, 3.233 |
|  |  |  |  |  |  |
| Thigh | 1000 | 0.049 | 49.349 | 0.057, 1.604, 0.175 | 1.975, 1.273, 2.979 |
| Shank | 1000 | 0.02 | 19.988 | 0.188, 0.966, 0.15 | 0.864, 0.165, 0.953 |
| Metatarsus | 1000 | 0.004 | 4.446 | 0.221, 0.393, 0.114 | 0.082, 0.089, 0.066 |
| Digit II | 1000 | 0.001 | 0.656 | 0.444, 0.159, 0.051 | 0.001, 0.003, 0.004 |
| Digit III | 1000 | 0.001 | 0.807 | 0.51, 0.157, 0.106 | 0.003, 0.006, 0.009 |
| Digit IV | 1000 | 0.001 | 0.799 | 0.441, 0.157, 0.142 | 0.002, 0.004, 0.005 |
| Pes | 1000 | 0.003 | 2.262 | 0.466,0.158,0.103 | - |
| Hind limb | 1000 | 0.076 | 76.045 | 0.088, 1.196, 0.163 | - |
|  |  |  |  |  |  |
| HAT | 883.12 | 0.355 | 313.509 | 0.397, 1.674, 0 | 21.821, 123.719, 139.372 |
| Whole Body | 918.34 | 0.507 | 465.599 | 0.296, 1.518, 0 | - |

Table S23. Results for the Minus 7.5% body/Plus 15% Tail model of *Struthiomimus* *sedens* BHI 1266.

| Segment | Net Density (kg m-3) | Volume (m3) | Mass (kg) | CM (x,y,z) (m) | Ixx Iyy Izz (kg m2) |
| --- | --- | --- | --- | --- | --- |
| Head | 974.01 | 0.0016 | 1.649 | 1.894, 2.302, 0 | 0.641, 4.551, 5.189 |
| Air sacs | 0 | 0.004 | 0 | - | - |
|  |  |  |  |  |  |
| Neck | 890.35 | 0.017 | 15.136 | 1.342, 1.924, 0 | 1.943, 19.217, 20.250 |
| Pharyngeal cavity | 0 | 0.002 | 0 | - | - |
|  |  |  |  |  |  |
| Thorax | 794.76 | 0.133 | 105.703 | 0.653, 1.660, 0 | 4.838, 23.819, 26.941 |
| Lungs | 0 | 0.027 | 0 | - | - |
|  |  |  |  |  |  |
| Sacrum | 1000 | 0.082 | 81.723 | -0.019, 1.639, 0 | 2.863, 9.146, 11.460 |
|  |  |  |  |  |  |
| Tail | 1000 | 0.049 | 49.185 | -0.858, 1.818, 0 | 5.00, 68.586, 73.294 |
|  |  |  |  |  |  |
| Arm | 1000 | 0.008 | 8.042 | 0.887, 1.384, 0.227 | 1.371, 3.899, 4.390 |
| Digit I | 1000 | 0.0001 | 0.157 | 0.887, 1.384, 0.227 | 0.101, 0.123, 0.188 |
| Digit II | 1000 | 0.0001 | 0.141 | 1.053, 0.953, 0.335 | 0.105, 0.102, 0.174 |
| Digit III | 1000 | 0.000132 | 0.132 | 0.981, 0.904, 0.326 | 0.050, 0.048, 0.083 |
| Fore limb | 1000 | 0.008332 | 8.472 | 0.894,1.36,0.232 | 1.627, 4.173, 4.836 |
|  |  |  |  |  |  |
| Thigh | 1000 | 0.049 | 49.349 | 0.057, 1.604, 0.175 | 1.975, 1.273, 2.979 |
| Shank | 1000 | 0.02 | 19.988 | 0.188, 0.966, 0.15 | 0.864, 0.165, 0.953 |
| Metatarsus | 1000 | 0.004 | 4.446 | 0.221, 0.393, 0.114 | 0.082, 0.089, 0.066 |
| Digit II | 1000 | 0.001 | 0.656 | 0.444, 0.159, 0.051 | 0.001, 0.003, 0.004 |
| Digit III | 1000 | 0.001 | 0.807 | 0.51, 0.157, 0.106 | 0.003, 0.006, 0.009 |
| Digit IV | 1000 | 0.001 | 0.799 | 0.441, 0.157, 0.142 | 0.002, 0.004, 0.005 |
| Pes | 1000 | 0.003 | 2.262 | 0.466, 0.158, 0.103 | - |
| Hind limb | 1000 | 0.076 | 76.045 | 0.088, 1.196, 0.163 | - |
|  |  |  |  |  |  |
| HAT | 866.98 | 0.312 | 270.497 | 0.235, 1.681, 0 | 18.539, 133.664, 146.806 |
| Whole Body | 910.75 | 0.464 | 422.587 | 0.182406 1.50601 0 | - |

Table S24. Results for the Plus 15% body/Minus 7.5% Tail model of *Edmontosaurus* *annectens* BHI 126950

| Segment | Net Density (kg m-3) | Volume (m3) | Mass (kg) | CM (x,y,z) (m) | Ixx Iyy Izz (kg m2) |
| --- | --- | --- | --- | --- | --- |
| Head | 962.4 | 0.028 | 27.17 | 1.082, 1.623, 0 | 5.288, 44.997, 50.065 |
| Air sacs | 0 | 0.006 | 0 | - | - |
|  |  |  |  |  |  |
| Neck | 1000 | 0.032 | 32.178 | 0.837, 1.202, 0 | 1.173, 29.605, 30.317 |
|  |  |  |  |  |  |
| Thorax | 814.63 | 0.334 | 272.085 | 0.228, 1.026, 0 | 29.561, 49.436, 66.047 |
| Lungs | 0 | 0.062 | 0 | - | - |
|  |  |  |  |  |  |
| Sacrum | 1000 | 0.177 | 176.939 | -0.589, 1.275, 0 | 10.798, 55.372, 64.007 |
|  |  |  |  |  |  |
| Tail | 1000 | 0.06 | 60.26 | -1.556, 1.366, 0 | 3.715, 133.099, 136.581 |
|  |  |  |  |  |  |
| Fore limb | 1000 | 0.011 | 11.031 | 0.618, 0.578, 0.129 | 4.206, 6.242, 10.037 |
|  |  |  |  |  |  |
| Thigh | 1000 | 0.112 | 112..431 | -0.588, 1.184, 0.197 | 3.984, 6.623, 9.137 |
| Shank | 1000 | 0.23 | 22.757 | -0.346, 0.575, 0.237 | 0.655, 0.261, 0.788 |
| Metatarsus | 1000 | 0.005 | 4.582 | -0.396, 0.2, 0.203 | 0.023, 0.023, 0.020 |
| Digit II | 1000 | 0.001 | 1.158 | -0.299, 0.084, 0.051 | 0.003, 0.004, 0.003 |
| Digit III | 1000 | 0.001 | 1.457 | -0.296, 0.095, 0.184 | 0.003, 0.007, 0.007 |
| Digit IV | 1000 | 0.001 | 0.925 | -0.296, 0.061, 0.281 | 0.002, 0.003, 0.003 |
| Pes | 1000 | 0.004 | 4.473 | -0.27, 0.077, 0.171 | - |
| Hind limb | 1000 | 0.144 | 144.243 | -0.507, 0.976, 0.203 | - |
|  |  |  |  |  |  |
| HAT | 896.35 | 0.659 | 590.694 | -0.112, 1.154, 0 | 59.927, 319.389, 362.466 |
| Whole Body | 928.38 | 0.947 | 879.18 | -0.241, 1.095, 0 | - |

Table S25. Results for the Minus 7.5% body/Plus 15% Tail model of *Edmontosaurus* BHI 126950.

| Segment | Net Density (kg m-3) | Volume (m3) | Mass (kg) | CM (x,y,z) (m) | Ixx Iyy Izz (kg m2) |
| --- | --- | --- | --- | --- | --- |
| Head | 962.4 | 0.028 | 27.17 | 1.082, 1.623, 0 | 4.794, 34.331, 38.905 |
| Air sacs | 0 | 0.006 | 0 | - | - |
|  |  |  |  |  |  |
| Neck | 1000 | 0.021 | 21.336 | 0.837, 1.202, 0 | 0.759, 16.704, 17.252 |
|  |  |  |  |  |  |
| Thorax | 732.81 | 0.231 | 169.28 | 0.249, 1.098,  0 | 14.072, 23.381, 31.595 |
| Lungs | 0 | 0.062 | 0 | - | - |
|  |  |  |  |  |  |
| Sacrum | 1000 | 0.177 | 176.939 | -0.589, 1.275, 0 | 8.823, 70.163, 76.823 |
|  |  |  |  |  |  |
| Tail | 1000 | 0.095 | 94.58 | -1.556, 1.346, 0 | 4.115, 13.263, 16.811 |
|  |  |  |  |  |  |
| Fore limb | 1000 | 0.011 | 11.031 | 0.618, 0.578, 0.129 | 5.035, 5.014, 9.637 |
|  |  |  |  |  |  |
| Thigh | 1000 | 0.112 | 112..431 | -0.588, 1.184, 0.197 | 3.984, 6.623, 9.137 |
| Shank | 1000 | 0.23 | 22.757 | -0.346, 0.575, 0.237 | 0.655, 0.261, 0.788 |
| Metatarsus | 1000 | 0.005 | 4.582 | -0.396, 0.2, 0.203 | 0.023, 0.023, 0.020 |
| Digit II | 1000 | 0.001 | 1.158 | -0.299, 0.084, 0.051 | 0.003, 0.004, 0.003 |
| Digit III | 1000 | 0.001 | 1.457 | -0.296, 0.095, 0.184 | 0.003, 0.007, 0.007 |
| Digit IV | 1000 | 0.001 | 0.925 | -0.296, 0.061, 0.281 | 0.002, 0.003, 0.003 |
| Pes | 1000 | 0.004 | 4.473 | -0.27, 0.077, 0.171 | - |
| Hind limb | 1000 | 0.144 | 144.243 | -0.507, 0.976, 0.203 | - |
|  |  |  |  |  |  |
| HAT | 883.19 | 0.579 | 511.367 | -0.261, 1.216, 0 | 42.633, 167.869, 200.660 |
| Whole Body | 921.49 | 0.868 | 799.853 | -0.343, 1.129, 0 | - |

Table S26. Results for the best estimate model of *Tyrannosaurus rex* BHI 3033 with enlarged body air sacs.

| Segment | Net Density (kg m-3) | Volume (m3) | Mass (kg) | CM (x,y,z) (m) | Ixx Iyy Izz (kg m2) |
| --- | --- | --- | --- | --- | --- |
| Head | 990.6 | 0.685 | 678.561 | 2.553, 3.346, 0 | 68.175, 1463.9, 1509.02 |
| Air sacs | 0 | 0.01 | 0 | - | - |
|  |  |  |  |  |  |
| Neck |  |  | 323.798 | 1.699, 3.394, 0 | 815.795, 2193.91, 2617.13 |
| Pharyngeal cavity | 0 | 0.045 | 0 | - | - |
|  |  |  |  |  |  |
| Thorax |  | 3.01 | 2128.95 | 0.387, 2.974, 0 | 831.162, 2220.43, 2646.88 |
| Lungs | 0 | 0.881 | 0 | - | - |
|  |  |  |  |  |  |
| Sacrum | 1000 | 1.062 | 1062.439 | -1.319, 3.004, 0 | 272.875, 1122.44, 1363.04 |
|  |  |  |  |  |  |
| Tail | 1000 | 1.106 | 1106.037 | -3.530, 3.092, 0 | 294.969, 12198.3, 12463.7 |
|  |  |  |  |  |  |
| Arm | 1000 | 0.011 | 10.931 | 1.138, 2.398, 0.483 | 7.78227, 28.8406, 31.3441 |
| Digit I | 1000 | 0.001 | 0.644 | 1.315, 2.115, 0.62 | 0.843, 2.164, 2.512 |
| Digit II | 1000 | 0.001 | 0.548 | 1.33, 2.197, 0.591 | 0.615, 1.851, 2.082 |
| Fore limb | 1000 | 0.013 | 12.123 | 1.156, 2.374, 0.495 | 9.241, 32.856, 35.938 |
|  |  |  |  |  |  |
| Thigh | 1000 | 0.744 | 743.937 | -1.323, 2.853, 0.408 | 183.542, 78.453, 226.284 |
| Shank | 1000 | 0.215 | 214.664 | -1.887, 1.687, 0.357 | 13.856, 21.791, 30.382 |
| Metatarsus | 1000 | 0.074 | 73.812 | -2.431, 0.914, 0.298 | 3.380, 1.501, 3.258 |
| Digit II | 1000 | 0.023 | 22.332 | -1.897, 0.11, 0.29 | 0.257, 0.259, 0.499 |
| Digit III | 1000 | 0.027 | 26.564 | -1.811, 0.134, 0.457 | 0.308, 0.540, 0.781 |
| Digit IV | 1000 | 0.021 | 20.966 | -1.998, 0.09, 0.571 | 0.241, 0.242, 0.452 |
| Pes | 1000 | 0.07 | 69.863 | -2.112, 0.681, 0.72 | - |
| Hind limb | 1000 | 1.102 | 1102.276 | -1.37, 2.194, 0.410 | - |
|  |  |  |  |  |  |
| HAT | 850.43 | 6.258 | 5322.03 | -0.409, 3.076, 0 | 1614.521, 23067.66, 24139.34 |
| Whole Body | 889.35 | 8.463 | 7526.58 | -0.692, 2.818, 0 | - |

Table S27. Results for the best estimate model of *Tyrannosaurus rex* BHI 3033 with reduced body air sacs.

| Segment | Net Density (kg m-3) | Volume (m3) | Mass (kg) | CM (x,y,z) (m) | Ixx Iyy Izz (kg m2) |
| --- | --- | --- | --- | --- | --- |
| Head | 990.6 | 0.685 | 678.561 | 2.553, 3.346, 0 | 151.755, 5831.7, 5930.34 |
| Air sacs | 0 | 0.01 | 0 | - | - |
|  |  |  |  |  |  |
| Neck |  | 0.369 | 344.244 | 1.683, 3.366, 0 | 73.832, 1465.05, 1515.48 |
| Pharyngeal cavity | 0 | 0.025 | 0 | - | - |
|  |  |  |  |  |  |
| Thorax |  | 3.01 | 2430 | 0.385, 2.944, 0 | 842.915, 2271.49, 2689.48 |
| Lungs | 0 | 0.58 | 0 | - | - |
|  |  |  |  |  |  |
| Sacrum | 1000 | 1.062 | 1062.439 | -1.319, 3.004, 0 | 270.346, 1218.29, 1456.37 |
|  |  |  |  |  |  |
| Tail | 1000 | 1.106 | 1106.037 | -3.530, 3.092, 0 | 296.062, 12534.2, 12800.8 |
|  |  |  |  |  |  |
| Arm | 1000 | 0.011 | 10.931 | 1.138, 2.398, 0.483 | 7.504, 27.232, 29.458 |
| Digit I | 1000 | 0.001 | 0.644 | 1.315, 2.115, 0.62 | 0.820, 2.059, 2.383 |
| Digit II | 1000 | 0.001 | 0.548 | 1.33, 2.197, 0.591 | 0.597, 1.760, 1.973 |
| Fore limb | 1000 | 0.013 | 12.123 | 1.156, 2.374, 0.495 | 8.921, 31.051, 33.814 |
|  |  |  |  |  |  |
| Thigh | 1000 | 0.744 | 743.937 | -1.323, 2.853, 0.408 | 183.542, 78.453, 226.284 |
| Shank | 1000 | 0.215 | 214.664 | -1.887, 1.687, 0.357 | 13.856, 21.791, 30.382 |
| Metatarsus | 1000 | 0.074 | 73.812 | -2.431, 0.914, 0.298 | 3.380, 1.501, 3.258 |
| Digit II | 1000 | 0.023 | 22.332 | -1.897, 0.11, 0.29 | 0.257, 0.259, 0.499 |
| Digit III | 1000 | 0.027 | 26.564 | -1.811, 0.134, 0.457 | 0.308, 0.540, 0.781 |
| Digit IV | 1000 | 0.021 | 20.966 | -1.998, 0.09, 0.571 | 0.241, 0.242, 0.452 |
| Pes | 1000 | 0.07 | 69.863 | -2.112, 0.681, 0.72 | - |
| Hind limb | 1000 | 1.102 | 1102.276 | -1.373, 2.19, 0.410 | - |
|  |  |  |  |  |  |
| HAT | 901.66 | 6.259 | 5643.52 | -0.361, 3.057, 0 | 1652.751, 23382.85, 24460.01 |
| Whole Body | 927.33 | 8.463 | 7848.07 | -0.645, 2.815, 0 | - |

Table S28. Results for the Plus 15% best estimate model of *Tyrannosaurus rex* BHI 3033 with reduced body air sacs.

| Segment | Net Density (kg m-3) | Volume (m3) | Mass (kg) | CM (x,y,z) (m) | Ixx Iyy Izz (kg m2) |
| --- | --- | --- | --- | --- | --- |
| Head | 990.6 | 0.685 | 678.561 | 2.553, 3.346, 0 | 159.623, 5986.66, 6093.16 |
| Air sacs | 0 | 0.01 | 0 | - | - |
|  |  |  |  |  |  |
| Neck |  | 0.472 | 447.339 | 1.695, 3.359, 0 | 114.931, 2002.6, 2077.38 |
| Pharyngeal cavity | 0 | 0.025 | 0 | - | - |
|  |  |  |  |  |  |
| Thorax |  | 4.72 | 3437.3 | 0.395, 2.940, 0 | 1527.83, 3579.82, 4297.66 |
| Lungs | 0 | 0.58 | 0 | - | - |
|  |  |  |  |  |  |
| Sacrum | 1000 | 1.166 | 1166.031 | -1.335, 2.984, 0 | 349.467, 1304.59, 1616.24 |
|  |  |  |  |  |  |
| Tail | 1000 | 1.468 | 1467.751 | -0.751, 3.079, 0 | 222.504, 16605.3, 16776.1 |
|  |  |  |  |  |  |
| Arm | 1000 | 0.011 | 10.931 | 1.138, 2.398, 0.483 | 7.238, 28.530, 30.490 |
| Digit I | 1000 | 0.001 | 0.644 | 1.315, 2.115, 0.62 | 0.798, 2.144, 2.446 |
| Digit II | 1000 | 0.001 | 0.548 | 1.33, 2.197, 0.591 | 0.580, 1.833, 2.029 |
| Total Fore limb | 1000 | 0.013 | 12.123 | 1.156, 2.374, 0.495 | 8.616, 32.507, 34.965 |
|  |  |  |  |  |  |
| Thigh | 1000 | 1.004 | 1004.155 | -1.337, 2.857, 0.46 | 263.403, 129.471, 319.34 |
| Shank | 1000 | 0.287 | 287.174 | -1.899, 1.69, 0.366 | 20.447, 32.873, 43.870 |
| Metatarsus | 1000 | 0.096 | 96.095 | -0.244, 0.921, 0.307 | 4.693, 2.604, 4.378 |
| Digit II | 1000 | 0.023 | 22.332 | -1.897, 0.11, 0.29 | 0.257, 0.259, 0.499 |
| Digit III | 1000 | 0.027 | 26.564 | -1.811, 0.134, 0.457 | 0.308, 0.540, 0.781 |
| Digit IV | 1000 | 0.021 | 20.966 | -1.998, 0.09, 0.571 | 0.241, 0.242, 0.452 |
| Pes | 1000 | 0.07 | 69.863 | -2.112, 0.681, 0.72 | - |
| Hind limb | 1000 |  | 1457.29 | -1.226, 2.229, 0.444 | - |
|  |  |  |  |  |  |
| HAT | 977.68 | 7.384 | 7219.22 | -0.400, 3.039, 0 | 2391.586, 29544.01, 30930.46 |
| Whole Body | 942.68 | 10.75 | 10133.8 | -0.637, 2.805, 0 | - |

Table S29. Results for the Minus 7.5% best estimate model of *Tyrannosaurus rex* BHI 3033 with enlarged air sacs.

| Segment | Net Density (kg m-3) | Volume (m3) | Mass (kg) | CM (x,y,z) (m) | Ixx Iyy Izz (kg m2) |
| --- | --- | --- | --- | --- | --- |
| Head | 990.6 | 0.685 | 678.561 | 2.553, 3.346, 0 | 138.39, 5831.79, 5917.06 |
| Air sacs | 0 | 0.01 | 0 | - | - |
|  |  |  |  |  |  |
| Neck |  | 0.342 | 296.458 | 1.717, 3.392, 0 | 53.0941, 1304.58, 1339.83 |
| Pharyngeal cavity | 0 | 0.045 | 0 | - | - |
|  |  |  |  |  |  |
| Thorax |  | 2.722 | 1840.15 | 0.376, 3.002, 0 | 644.22, 1706.37, 2027.71 |
| Lungs | 0 | 0.881 | 0 | - | - |
|  |  |  |  |  |  |
| Sacrum | 1000 | 1.044 | 1044.22 | -1.315, 3.003, 0 | 268.531, 1182.39, 1420.24 |
|  |  |  |  |  |  |
| Tail | 1000 | 0.942 | 942.361 | -3.526, 3.099, 0 | 104.647, 10798.4, 10881.7 |
|  |  |  |  |  |  |
| Arm | 1000 | 0.011 | 10.931 | 1.138, 2.398, 0.483 | 8.018, 27.233, 29.972 |
| Digit I | 1000 | 0.001 | 0.644 | 1.315, 2.115, 0.62 | 0.863, 2.059, 2.426 |
| Digit II | 1000 | 0.001 | 0.548 | 1.33, 2.197, 0.591 | 0.630, 1.760, 2.007 |
| Total Fore limb | 1000 | 0.013 | 12.123 | 1.156, 2.374, 0.495 | 9.511, 31.052, 34.405 |
|  |  |  |  |  |  |
| Thigh | 1000 | 0.661 | 660.703 | -1.329, 2.851, 0.386 | 163.536, 65.890, 198.758 |
| Shank | 1000 | 0.184 | 183.605 | -1.881, 1.684, 0.352 | 11.389, 17.820, 25.388 |
| Metatarsus | 1000 | 0.062 | 62.105 | -2.427, 0.911, 0.293 | 2.677, 1.095, 2.599 |
| Digit II | 1000 | 0.023 | 22.332 | -1.897, 0.11, 0.29 | 0.257, 0.259, 0.499 |
| Digit III | 1000 | 0.027 | 26.564 | -1.811, 0.134, 0.457 | 0.308, 0.540, 0.781 |
| Digit IV | 1000 | 021 | 20.966 | -1.998, 0.09, 0.571 | 0.241, 0.242, 0.452 |
| Pes | 1000 | 0.07 | 69.863 | -2.112, 0.681, 0.72 | - |
| Hind limb | 1000 | 1.103 | 976.276 | -1.382, 2.195, 0.398 | - |
|  |  |  |  |  |  |
| HAT | 837.5 | 5.76 | 4823.98 | -0.361, 3.091, 0 | 1227.901, 20885.65, 21655.4 |
| Whole Body | 878.59 | 7.713 | 6776.54 | -0.655, 2.833, 0 | - |

Table S30. Results for the best estimate model of *Tyrannosaurus rex* MOR 555 with enlarged body air sacs.

| Segment | Net Density (kg m-3) | Volume (m3) | Mass (kg) | CM (x,y,z) (m) | Ixx Iyy Izz (kg m2) |
| --- | --- | --- | --- | --- | --- |
| Head | 984.26 | 0.661 | 650.596 | 3.884, 3.063, 0 | 232.671, 5795.22, 5967.96 |
| Air sacs | 0 | 0.011 | 0 | - | - |
|  |  |  |  |  |  |
| Neck |  | 0.471 | 434.905 | 2.785, 3.107, 0 | 144.447, 1633.31, 1770.17 |
| Pharyngeal cavity | 0 | 0.036 | 0 | - | - |
|  |  |  |  |  |  |
| Thorax |  | 1.721 | 1243.21 | 1.388, 2.630, 0 | 338.386, 547.946, 858.659 |
| Lungs | 0 | 0.478 | 0 | - | - |
|  |  |  |  |  |  |
| Sacrum | 1000 | 0.659 | 659.477 | 0.222, 2.272, 0 | 285.134, 380.858, 655.477 |
|  |  |  |  |  |  |
| Tail | 1000 | 1.079 | 1078.774 | -1.828, 2.363, 0 | 177.253, 9426.57, 9581.97 |
|  |  |  |  |  |  |
| Arm | 1000 | 0.009 | 8.845 | 1.89, 2.097, 0.277 | 3.288, 9.494, 11.392 |
| Digit I | 1000 | 0.0003 | 0.355 | 2.16, 1.922, 0.202 | 0.187, 0.583, 0.741 |
| Digit II | 1000 | 0.001 | 0.822 | 2.172, 1.846, 0 | 0.525, 1.377, 1.835 |
| Fore limb | 1000 | 0.010 | 10.022 | 1.923, 0.294, 0.268 | 4.001, 11.454, 13.968 |
|  |  |  |  |  |  |
| Thigh | 1000 | 0.689 | 688.552 | -0.114, 2.289, 0.363 | 149.268, 76.0171, 194.264 |
| Shank | 1000 | 0.212 | 212.459 | -0.584, 1.114, 0.426 | 13.684, 19.713, 27.550 |
| Metatarsus | 1000 | 0.044 | 44.408 | -0.859, 0.392, 0.417 | 1.204, 0.906, 1.343 |
| Digit II | 1000 | 0.007 | 7.128 | -0.477, 0.077, 0.25 | 0.038, 0.166, 0.155 |
| Digit III | 1000 | 0.01 | 9.765 | -0.432, 0.09, 0.451 | 0.036, 0.336, 0.331 |
| Digit IV | 1000 | 0.009 | 8.814 | -0.489, 0.091, 0.614 | 0.054, 0.227, 0.203 |
| Pes | 1000 | 0.026 | 25.707 | -0.464, 0.086, 0.449 | - |
| Hind limb | 1000 | 0.971 | 971.126 | -0.220, 1.760, 0.382 | - |
|  |  |  |  |  |  |
| HAT | 885.94 | 4.612 | 4086.77 | 0.895, 2.619, 0 | 1185.891, 17806.85, 18862.17 |
| Whole Body | 920 | 6.56 | 6035.35 | 0.535, 2.342, 0 | - |

Table S31. Results for the best estimate model of *Tyrannosaurus rex* MOR 555 with reduced body air sacs.

| Segment | Net Density (kg m-3) | Volume (m3) | Mass (kg) | CM (x,y,z) (m) | Ixx Iyy Izz (kg m2) |
| --- | --- | --- | --- | --- | --- |
| Head | 984.26 | 0.661 | 650.596 | 3.884, 3.063, 0 | 239.349, 5709.24, 5888.65 |
| Air sacs | 0 | 0.011 | 0 | - | - |
|  |  |  |  |  |  |
| Neck |  | 0.471 | 447.943 | 2.783, 3.099, 0 | 151.675, 1643.39, 1778.63 |
| Pharyngeal cavity | 0 | 0.023 | 0 | - | - |
|  |  |  |  |  |  |
| Thorax |  | 1.721 | 1361.36 | 1.397, 2.592, 0 | 352.84, 634.544, 827.169 |
| Lungs | 0 | 0.36 | 0 | - | - |
|  |  |  |  |  |  |
| Sacrum | 1000 | 0.659 | 659.477 | 0.222, 2.272, 0 | 280.027, 401.077, 670.589 |
|  |  |  |  |  |  |
| Tail | 1000 | 1.079 | 1078.774 | -1.828, 2.363, 0 | 171.132, 9558.77, 9708.05 |
|  |  |  |  |  |  |
| Arm | 1000 | 0.009 | 8.845 | 1.89, 2.097, 0.277 | 3.184, 9.104, 10.898 |
| Digit I | 1000 | 0.0003 | 0.355 | 2.16, 1.922, 0.202 | 0.182, 0.563, 0.716 |
| Digit II | 1000 | 0.001 | 0.822 | 2.172, 1.846, 0 | 0.511, 1.331, 1.774 |
| Fore limb | 1000 | 0.010 | 10.022 | 1.923, 0.294, 0.268 | 3.877, 10.997, 13.387 |
|  |  |  |  |  |  |
| Thigh | 1000 | 0.689 | 688.552 | -0.114, 2.289, 0.363 | 149.268, 76.017, 194.264 |
| Shank | 1000 | 0.212 | 212.459 | -0.584, 1.114, 0.426 | 13.684, 19.713, 27.550 |
| Metatarsus | 1000 | 0.044 | 44.408 | -0.859, 0.392, 0.417 | 1.204, 0.906, 1.343 |
| Digit II | 1000 | 0.007 | 7.128 | -0.477, 0.077, 0.25 | 0.038, 0.166, 0.155 |
| Digit III | 1000 | 0.01 | 9.765 | -0.432, 0.09, 0.451 | 0.036, 0.336, 0.331 |
| Digit IV | 1000 | 0.009 | 8.814 | -0.489, 0.091, 0.614 | 0.054, 0.227, 0.203 |
| Pes | 1000 | 0.026 | 25.707 | -0.464, 0.086, 0.449 | - |
| Hind limb | 1000 | 0.971 | 971.126 | -0.220, 1.760, 0.382 | - |
|  |  |  |  |  |  |
| HAT | 914.56 | 4.612 | 4217.95 | 0.917, 2.607, 0 | 1202.777, 17969, 18899.89 |
| Whole Body | 939.88 | 6.561 | 6166.54 | 0.558, 2.340, 0 | - |

Table S32. Results for the Plus 15% best estimate model of *Tyrannosaurus rex* MOR 555 with reduced air sacs.

| Segment | Net Density (kg m-3) | Volume (m3) | Mass (kg) | CM (x,y,z) (m) | Ixx Iyy Izz (kg m2) |
| --- | --- | --- | --- | --- | --- |
| Head | 984.26 | 0.661 | 650.596 | 3.883, 3.063, 0 | 294.188, 7650.37, 7848.75 |
| Air sacs | 0 | 0.011 | 0 | - | - |
|  |  |  |  |  |  |
| Neck | 951.77 | 0.607 | 583.799 | 2.799, 3.186, 0 | 248.817, 2060.07, 2282.49 |
| Pharyngeal cavity | 0 | 0.023 | 0 | - | - |
|  |  |  |  |  |  |
| Thorax | 804.21 | 2.29 | 1930.3 | 1.436, 2.621, 0 | 675.62, 901.146, 1278.6 |
| Lungs | 0 | 0.36 | 0 | - | - |
|  |  |  |  |  |  |
| Sacrum | 1000 | 0.679 | 659.477 | 0.214, 2.26, 0 | 317.366, 476.017, 782.734 |
|  |  |  |  |  |  |
| Tail | 1000 | 1.421 | 1420.774 | -1.848, 2.178, 0 | 296.654, 13155.4, 13414.2 |
|  |  |  |  |  |  |
| Arm | 1000 | 0.009 | 8.845 | 1.89, 2.097, 0.277 | 3.412, 8.230, 10.252 |
| Digit I | 1000 | 0.00025458 | 0.355 | 2.16, 1.922, 0.202 | 0.194, 0.518, 0.683 |
| Digit II | 1000 | 0.001 | 0.822 | 2.172, 1.846, 0 | 0.542, 1.225, 1.699 |
| Total Fore limb | 1000 | 0.010254576 | 10.022 | 1.923, 0.294, 0.268 | 4.148, 9.973, 12.634 |
|  |  |  |  |  |  |
| Thigh | 1000 | 0.885 | 884.663 | -0.106, 2.328, 0.413 | 210.547, 104.1, 263.14 |
| Shank | 1000 | 0.285 | 284.567 | -0.596, 1.107, 0.432 | 20.668, 29.298, 39.617 |
| Metatarsus | 1000 | 0.057 | 56.684 | -0.866, 0.382, 0.423 | 1.617, 1.391, 1.726 |
| Digit II | 1000 | 0.007 | 7.128 | -0.477, 0.077, 0.25 | 0.038, 0.166, 0.155 |
| Digit III | 1000 | 0.01 | 9.765 | -0.432, 0.09, 0.451 | 0.036, 0.336, 0.331 |
| Digit IV | 1000 | 0.009 | 8.814 | -0.489, 0.091, 0.614 | 0.055, 0.227, 0.203 |
| Pes | 1000 | 0.026 | 25.707 | -0.464, 0.086, 0.449 | - |
| Hind limb | 1000 | 0.971 | 1251.62 | -0.210, 1.786, 0.419 | - |
|  |  |  |  |  |  |
| HAT | 933.02 | 5.881 | 5487.07 | 0.970, 2.632, 0 | 1840.938, 24262.97, 25632.03 |
| Whole Body | 953 | 8.391 | 7996.65 | 0.970, 2.632, 0 | - |

Table S33. Results for the Minus 7.5% best estimate model of *Tyrannosaurus rex* MOR 555 with enlarged air sacs.

| Segment | Net Density (kg m-3) | Volume (m3) | Mass (kg) | CM (x,y,z) (m) | Ixx Iyy Izz (kg m2) |
| --- | --- | --- | --- | --- | --- |
| Head | 984.26 | 0.661 | 650.596 | 3.884, 3.063, 0 | 224.462, 5574, 5738.53 |
| Air sacs | 0 | 0.011 | 0 | - | - |
|  |  |  |  |  |  |
| Neck | 931.82 | 0.425 | 389.058 | 2.790, 3.102, 0 | 118.441, 1380.81, 1494.31 |
| Pharyngeal cavity | 0 | 0.036 | 0 | - | - |
|  |  |  |  |  |  |
| Thorax | 718.6 | 1.594 | 1115.95 | 1.362, 2.664, 0 | 271.808, 402.631, 669.416 |
| Lungs | 0 | 0.478 | 0 | - | - |
|  |  |  |  |  |  |
| Sacrum | 1000 | 0.669 | 669.434 | 0.23, 2.279, 0 | 292.735, 435.245, 716.196 |
|  |  |  |  |  |  |
| Tail | 1000 | 0.941 | 940.792 | -1.803, 2.369, 0 | 149.605, 8392.19, 8525.27 |
|  |  |  |  |  |  |
| Arm | 1000 | 0.009 | 8.845 | 1.89, 2.097, 0.277 | 3.422, 8.502, 10.535 |
| Digit I | 1000 | 0.0003 | 0.355 | 2.16, 1.922, 0.202 | 0.194, 0.532, 0.697 |
| Digit II | 1000 | 0.001 | 0.822 | 2.172, 1.846, 0 | 0.544, 1.258, 1.734 |
| Total Fore limb | 1000 | 0.010 | 10.022 | 1.923, 0.294, 0.268 | 4.161, 10.293, 12.966 |
|  |  |  |  |  |  |
| Thigh | 1000 | 0.63 | 629.98 | -0.116, 2.305, 0.349 | 134.877, 68.013, 177.073 |
| Shank | 1000 | 0.181 | 181.07 | -0.576, 1.119, 0.423 | 10.990, 16.075, 22.792 |
| Metatarsus | 1000 | 0.039 | 38.872 | -0.855, 0.396, 0.413 | 1.041, 0.726, 1.186 |
| Digit II | 1000 | 0.007 | 7.128 | -0.477, 0.077, 0.25 | 0.038, 0.166, 0.155 |
| Digit III | 1000 | 0.01 | 9.765 | -0.432, 0.09, 0.451 | 0.036, 0.336, 0.331 |
| Digit IV | 1000 | 0.009 | 8.814 | -0.489, 0.091, 0.614 | 0.055, 0.227, 0.203 |
| Pes | 1000 | 0.026 | 25.707 | -0.464, 0.086, 0.449 | - |
| Hind limb | 1000 | 0.876 | 875.629 | -0.221, 1.789, 0.370 | - |
|  |  |  |  |  |  |
| HAT | 870.863 | 4.347 | 3785.64 | 0.953, 2.633, 0 | 1065.371, 16205.49, 17169.67 |
| Whole Body | 907.982 | 6.105 | 5543.23 | 0.581, 2.365, 0 | - |

Table S34. Results for the best estimate model of *Acrocanthosaurus atokensis* NCSM 14345 with enlarged body air sacs.

| Segment | Net Density (kg m-3) | Volume (m3) | Mass (kg) | CM (x,y,z) (m) | Ixx Iyy Izz (kg m2) |
| --- | --- | --- | --- | --- | --- |
| Head | 981.63 | 0.405 | 397.566 | 3.437, 2.138, 0 | 45.8983, 4427.07, 4458.98 |
| Air sacs | 0 | 0.007 | 0 | - | - |
|  |  |  |  |  |  |
| Neck | 891.015 | 0.336 | 299.381 | 2.327, 2.181, 0 | 38.3099, 1499.01, 1526.19 |
| Pharyngeal cavity | 0 | 0.037 | 0 | - | - |
|  |  |  |  |  |  |
| Thorax | 723.351 | 2.42 | 1750.51 | 1.062, 2.181, 0 | 518.572, 2479.95, 2660.6 |
| Lungs | 0 | 0.669 | 0 | - | - |
|  |  |  |  |  |  |
| Sacrum | 1000 | 0.768 | 768.158 | -0.54,  2.41, 0 | 193.405, 455.636, 634.72 |
|  |  |  |  |  |  |
| Tail | 1000 | 1.149 | 1148.734 | -2.77, 2.465, 0 | 127.772, 10862.9, 10960.7 |
|  |  |  |  |  |  |
| Arm | 1000 | 0.01 | 10.024 | 1.694, 1.252, -0.414 | 12.7481, 27.814, 36.987 |
| Digit I | 1000 | 0.0005 | 0.491 | 1.721, 0.919, -0.585 | 1.086, 1.484, 2.235 |
| Digit II | 1000 | 0.001 | 1.207 | 1.814, 0.852, -0.531 | 2.829, 3.954, 6.101 |
| Digit IV | 1000 | 0.0006 | 0.639 | 1.815, 0.898, -0.422 | 1.346, 2.030, 3.147 |
| Forelimb | 1000 | 0.012 | 12.361 | 1.713, 1.181, 0.433 | 17.807, 34.521, 47.505 |
|  |  |  |  |  |  |
| Thigh | 1000 | 0.664 | 663.709 | -0.495, 2.067, 0.35 | 91.336, 97.478, 159.109 |
| Shank | 1000 | 0.142 | 142.12 | -0.255, 0.936, 0.308 | 11.385, 5.102, 13.239 |
| Metatarsus | 1000 | 0.033 | 32.925 | -0.277, 0.236, 0.32 | 0.670, 0.633, 0.762 |
| Digit II | 1000 | 0.002 | 2.257 | 0.023, 0.067, 0.185 | 0.003, 0.004, 0.004 |
| Digit III | 1000 | 0.003 | 3.647 | 0.105, 0.026, 0.341 | 0.006, 0.008, 0.008 |
| Digit IV | 1000 | 0.002 | 1.767 | 0.019, 0.071, 0.491 | 0.002, 0.002, 0.003 |
| Pes | 1000 | 0.007 | 7.671 | 0.062, 0.049, 0.33 | - |
| Hind limb | 1000 | 0.847 | 846.524 | -0.441, 1.788, 0.342 | - |
|  |  |  |  |  |  |
| HAT | 860.26 | 5.102 | 4389.07 | 0.083, 2.286, 0 | 905.991, 19816.48, 20362.47 |
| Whole Body | 895.12 | 6.794 | 6081.44 | -0.062, 2.147, 0 | - |

Table S35. Results for the best estimate model of *Acrocanthosaurus atokensis* NCSM 14345 with reduced body air sacs.

| Segment | Net Density (kg m-3) | Volume (m3) | Mass (kg) | CM (x,y,z) (m) | Ixx Iyy Izz (kg m2) |
| --- | --- | --- | --- | --- | --- |
| Head | 981.63 | 0.405 | 397.566 | 3.437, 2.138, 0 | 43.9586, 4401.24, 4431.21 |
| Air sacs | 0 | 0.007 | 0 | - | - |
|  |  |  |  |  |  |
| Neck | 932.631 | 0.336 | 313.364 | 2.322, 2.180, 0 | 39.1631, 1547.21, 1575.02 |
| Pharyngeal cavity | 0 | 0.023 | 0 | - | - |
|  |  |  |  |  |  |
| Thorax | 810.12 | 2.42 | 1960.49 | 1.051, 2.158, 0 | 526.037, 2364.23, 2593.12 |
| Lungs | 0 | 0.459 | 0 | - | - |
|  |  |  |  |  |  |
| Sacrum | 1000 | 0.768 | 768.158 | -0.54,  2.41, 0 | 196.485, 501.629, 683.793 |
|  |  |  |  |  |  |
| Tail | 1000 | 1.149 | 1148.734 | -2.77, 2.465, 0 | 134.302, 11168.8, 11273.1 |
|  |  |  |  |  |  |
| Arm | 1000 | 0.01 | 10.024 | 1.694, 1.252, -0.414 | 12.434, 26.342, 35.202 |
| Digit I | 1000 | 0.0005 | 0.491 | 1.721, 0.919, -0.585 | 1.066, 1.411, 2.141 |
| Digit II | 1000 | 0.001 | 1.207 | 1.814, 0.852, -0.531 | 2.777, 3.764, 5.858 |
| Digit IV | 1000 | 0.0006 | 0.639 | 1.815, 0.898, -0.422 | 1.319, 1.929, 3.019 |
| Forelimb | 1000 | 0.012 | 12.361 | 1.713, 1.181, 0.433 | 17.597, 33.445, 46.220 |
|  |  |  |  |  |  |
| Thigh | 1000 | 0.664 | 663.709 | -0.495, 2.067, 0.35 | 91.336, 97.478, 159.109 |
| Shank | 1000 | 0.142 | 142.12 | -0.255, 0.936, 0.308 | 11.385, 5.102, 13.239 |
| Metatarsus | 1000 | 0.033 | 32.925 | -0.277, 0.236, 0.32 | 0.670, 0.633, 0.762 |
| Digit II | 1000 | 0.002 | 2.257 | 0.023, 0.067, 0.185 | 0.003, 0.004, 0.004 |
| Digit III | 1000 | 0.003 | 3.647 | 0.105, 0.026, 0.341 | 0.006, 0.008, 0.008 |
| Digit IV | 1000 | 0.002 | 1.767 | 0.019, 0.071, 0.491 | 0.002, 0.002, 0.003 |
| Pes | 1000 | 0.007 | 7.671 | 0.062,0.049,0.33 | - |
| Hind limb | 1000 | 0.847 | 846.524 | -0.441, 1.788, 0.342 | - |
|  |  |  |  |  |  |
| HAT | 904.16 | 5.102 | 4613.03 | 0.130, 2.271, 0 | 975.139, 20049.95, 20648.72 |
| Whole Body | 928.08 | 6.794 | 6305.39 | -0.023, 2.141, 0 | - |

Table S36. Results for the Plus 15% best estimate model of *Acrocanthosaurus atokensis* NCSM 14345 with reduced body air sacs.

| Segment | Net Density (kg m-3) | Volume (m3) | Mass (kg) | CM (x,y,z) (m) | Ixx Iyy Izz (kg m2) |
| --- | --- | --- | --- | --- | --- |
| Head | 981.64 | 0.405 | 397.566 | 3.437, 2.138, 0 | 42.8993, 4616.4, 4645.31 |
| Air sacs | 0 | 0.007 | 0 | - | - |
|  |  |  |  |  |  |
| Neck | 944.12 | 0.407 | 384.257 | 2.338, 2.183, 0 | 53.229, 2063.99, 2101.83 |
| Pharyngeal cavity | 0 | 0.023 | 0 | - | - |
|  |  |  |  |  |  |
| Thorax | 828.678 | 3.214 | 2663.37 | 1.049, 2.121, 0 | 993.201, 3618.12, 4082.79 |
| Lungs | 0 | 0.459 | 0 | - | - |
|  |  |  |  |  |  |
| Sacrum | 1000 | 0.831 | 831.492 | -0.565,  2.435, 0 | 258.323, 486.04, 729.185 |
|  |  |  |  |  |  |
| Tail | 1000 | 1.57 | 1570.46 | -1.956, 2.471, 0 | 298.85, 14483., 14649 |
|  |  |  |  |  |  |
| Arm | 1000 | 0.01 | 10.024 | 1.694, 1.252, -0.414 | 12.223, 28.92, 37.590 |
| Digit I | 1000 | 0.0005 | 0.491 | 1.721, 0.919, -0.585 | 1.052, 1.540, 2.256 |
| Digit II | 1000 | 0.001 | 1.207 | 1.814, 0.852, -0.531 | 2.741, 4.101, 6.159 |
| Digit IV | 1000 | 0.0006 | 0.639 | 1.815, 0.898, -0.422 | 1.301, 2.107, 3.179 |
| Forelimb | 1000 | 0.012 | 12.361 | 1.713, 1.181, 0.433 | 17.318, 36.689, 49.184 |
|  |  |  |  |  |  |
| Thigh | 1000 | 0.77 | 770.088 | -0.478, 2.048, 0.382 | 109.384, 122.51, 187.825 |
| Shank | 1000 | 0.185 | 185.173 | -0.259, 0.934, 0.308 | 15.431, 8.203, 18.217 |
| Metatarsus | 1000 | 0.041 | 40.864 | -0.289, 0.238, 0.319 | 0.903, 0.892, 0.947 |
| Digit II | 1000 | 0.002 | 2.257 | 0.023, 0.067, 0.185 | 0.003, 0.004, 0.004 |
| Digit III | 1000 | 0.003 | 3.647 | 0.105, 0.026, 0.341 | 0.006, 0.008, 0.008 |
| Digit IV | 1000 | 0.002 | 1.767 | 0.019, 0.071, 0.491 | 0.002, 0.002, 0.003 |
| Pes | 1000 | 0.007 | 7.671 | 0.062, 0.049, 0.33 | - |
| Hind limb | 1000 | 1.003 | 1003.89 | 0.426, 1.754, 0.365 | - |
|  |  |  |  |  |  |
| HAT | 923.1 | 6.361 | 5871.86 | 0.049, 2.260, 0 | 1681.138, 25340.99, 26306.48 |
| Whole Body | 941.56 | 8.368 | 7878.97 | -0.072, 2.131, 0 | - |

Table S37. Results for the Minus 7.5% best estimate model of *Acrocanthosaurus atokensis* NCSM 14345 with enlarged body air sacs.

| Segment | Net Density (kg m-3) | Volume (m3) | Mass (kg) | CM (x,y,z) (m) | Ixx Iyy Izz (kg m2) |
| --- | --- | --- | --- | --- | --- |
| Head | 981.64 | 0.405 | 397.566 | 3.437, 2.138, 0 | 46.082, 4399.56, 4431.65 |
| Air sacs | 0 | 0.007 | 0 | - | - |
|  |  |  |  |  |  |
| Neck | 870 | 0.28 | 243.6 | 2.348, 2.211, 0 | 24.154, 1229.87, 1246.48 |
| Pharyngeal cavity | 0 | 0.037 | 0 | - | - |
|  |  |  |  |  |  |
| Thorax | 668.111 | 2.319 | 1549.35 | 1.062, 2.195, 0 | 349.881, 1968.22, 2079.81 |
| Lungs | 0 | 0.669 | 0 | - | - |
|  |  |  |  |  |  |
| Sacrum | 1000 | 0.745 | 744.899 | -0.554,  2.41, 0 | 184.958, 493.913, 665.784 |
|  |  |  |  |  |  |
| Tail | 1000 | 0.975 | 975.222 | -2.768, 2.457, 0 | 92.339, 9470.52, 9541.08 |
|  |  |  |  |  |  |
| Arm | 1000 | 0.01 | 10.024 | 1.694, 1.252, -0.414 | 12.821, 26.322, 35.568 |
| Digit I | 1000 | 0.0005 | 0.491 | 1.721, 0.919, -0.585 | 1.091, 1.410, 2.165 |
| Digit II | 1000 | 0.001 | 1.207 | 1.814, 0.852, -0.531 | 2.841, 3.761, 5.920 |
| Digit IV | 1000 | 0.0006 | 0.639 | 1.815, 0.898, -0.422 | 1.352, 1.927, 3.051 |
| Forelimb | 1000 | 0.012 | 12.361 | 1.713, 1.181, 0.433 | 18.106, 33.420, 46.704 |
|  |  |  |  |  |  |
| Thigh | 1000 | 0.611 | 610.558 | -0.505, 2.057, 0.347 | 80.863, 88.150, 143.494 |
| Shank | 1000 | 0.123 | 122.933 | -0.253, 0.938, 0.308 | 9.678, 3.925, 11.166 |
| Metatarsus | 1000 | 0.028 | 28.379 | -0.273, 0.238, 0.321 | 0.546, 0.508, 0.651 |
| Digit II | 1000 | 0.002 | 2.257 | 0.023, 0.067, 0.185 | 0.003, 0.004, 0.004 |
| Digit III | 1000 | 0.003 | 3.647 | 0.105, 0.026, 0.341 | 0.006, 0.008, 0.008 |
| Digit IV | 1000 | 0.002 | 1.767 | 0.019, 0.071, 0.491 | 0.002, 0.002, 0.003 |
| Pes | 1000 | 0.007 | 7.671 | 0.062, 0.049, 0.33 | - |
| Hind limb | 1000 | 0.770 | 769.64 | -0.451, 1.792, 0.340 | - |
|  |  |  |  |  |  |
| HAT | 846.68 | 4.648 | 3935.36 | 0.131, 2.290, 0. | 733.627, 17628.92, 18058.2 |
| Whole Body | 884.75 | 6.187 | 5473.96 | -0.033, 2.150, 0 | - |

Table S38. Results for the best estimate model of *Struthiomimus sedens* 1266 with enlarged body air sacs.

| Segment | Net Density (kg m-3) | Volume (m3) | Mass (kg) | CM (x,y,z) (m) | Ixx Iyy Izz (kg m2) |
| --- | --- | --- | --- | --- | --- |
| Head | 974.01 | 0.0016 | 1.649 | 1.894, 2.302, 0 | 0.649, 4.153, 4.799 |
| Air sacs | 0 | 0.004 | 0 | - | - |
|  |  |  |  |  |  |
| Neck | 857.45 | 0.02 | 17.419 | 1.341, 1.921, 0 | 1.812, 19.460, 21.467 |
| Pharyngeal cavity | 0 | 0.003 | 0 | - | - |
|  |  |  |  |  |  |
| Thorax | 778.07 | 0.142 | 110.486 | 0.647, 1.662, 0 | 5.378, 19.070, 22.383 |
| Lungs | 0 | 0.031 | 0 | - | - |
|  |  |  |  |  |  |
| Sacrum | 1000 | 0.082 | 81.723 | -0.019, 1.639, 0 | 2.839, 12.688, 14.979 |
|  |  |  |  |  |  |
| Tail | 1000 | 0.037 | 37.067 | -0.854, 1.827, 0 | 3.736, 56.745, 60.314 |
|  |  |  |  |  |  |
| Arm | 1000 | 0.008 | 8.042 | 0.887, 1.384, 0.227 | 1.359, 3.250, 3.729 |
| Digit I | 1000 | 0.0001 | 0.157 | 0.887, 1.384, 0.227 | 0.100, 0.107, 0.172 |
| Digit II | 1000 | 0.0001 | 0.141 | 1.053, 0.953, 0.335 | 0.105, 0.088, 0.160 |
| Digit III | 1000 | 0.0001 | 0.132 | 0.981, 0.904, 0.326 | 0.049, 0.042, 0.075 |
| Fore limb | 1000 | 0.008 | 8.472 | 0.894, 1.36, 0.232 | 1.614, 3.486, 4.136 |
|  |  |  |  |  |  |
| Thigh | 1000 | 0.049 | 49.349 | 0.057, 1.604, 0.175 | 1.974, 1.273, 2.979 |
| Shank | 1000 | 0.02 | 19.988 | 0.188, 0.966, 0.15 | 0.864, 0.165, 0.953 |
| Metatarsus | 1000 | 0.004 | 4.446 | 0.221, 0.393, 0.114 | 0.0820494, 0.089, 0.066 |
| Digit II | 1000 | 0.001 | 0.656 | 0.444, 0.159, 0.051 | 0.001, 0.003, 0.004 |
| Digit III | 1000 | 0.001 | 0.807 | 0.51, 0.157, 0.106 | 0.003, 0.006, 0.009 |
| Digit IV | 1000 | 0.001 | 0.799 | 0.441, 0.157, 0.142 | 0.002, 0.004, 0.005 |
| Pes | 1000 | 0.003 | 2.262 | 0.466, 0.158, 0.103 | - |
| Hind limb | 1000 | 0.076 | 76.045 | 0.088, 1.196, 0.163 | - |
|  |  |  |  |  |  |
| HAT | 873.17 | 0.304 | 265.445 | 0.300, 1.678, 0 | 17.647, 118.652, 131.783 |
| Whole Body | 915.65 | 0.456 | 417.535 | 0.223241 1.50233 0 | - |

Table S39. Results for the best estimate model of *Struthiomimus sedens* 1266 with reduced body air sacs.

| Segment | Net Density (kg m-3) | Volume (m3) | Mass (kg) | CM (x,y,z) (m) | Ixx Iyy Izz (kg m2) |
| --- | --- | --- | --- | --- | --- |
| Head | 974.01 | 0.0016 | 1.649 | 1.894, 2.302, 0 | 0.648, 4.109, 4.755 |
| Air sacs | 0 | 0.0004 | 0 | - | - |
|  |  |  |  |  |  |
| Neck | 928.85 | 0.02 | 18.577 | 1.337, 1.916, 0 | 1.914, 19.960, 21.939 |
| Pharyngeal cavity | 0 | 0.001 | 0 | - | - |
|  |  |  |  |  |  |
| Thorax | 841 | 0.142 | 119.422 | 0.653, 1.659, 0 | 4.581, 20.304, 22.745 |
| Lungs | 0 | 0.022 | 0 | - | - |
|  |  |  |  |  |  |
| Sacrum | 1000 | 0.082 | 81.723 | -0.019, 1.639, 0 | 2.840, 13.147, 15.438 |
|  |  |  |  |  |  |
| Tail | 1000 | 0.037 | 37.067 | -0.854, 1.827, 0 | 3.748, 58.2560, 61.841 |
|  |  |  |  |  |  |
| Arm | 1000 | 0.008 | 8.042 | 0.887, 1.384, 0.227 | 1.354, 3.086, 3.560 |
| Digit I | 1000 | 0.0001 | 0.157 | 0.887, 1.384, 0.227 | 0.100, 0.103, 0.167 |
| Digit II | 1000 | 0.0001 | 0.141 | 1.053, 0.953, 0.335 | 0.104, 0.084, 0.156 |
| Digit III | 1000 | 0.000132 | 0.132 | 0.981, 0.904, 0.326 | 0.049, 0.040, 0.073 |
| Fore limb | 1000 | 0.008332 | 8.472 | 0.894, 1.36, 0.232 | 1.608, 3.314, 3.957 |
|  |  |  |  |  |  |
| Thigh | 1000 | 0.049 | 49.349 | 0.057, 1.604, 0.175 | 1.974, 1.273, 2.979 |
| Shank | 1000 | 0.02 | 19.988 | 0.188, 0.966, 0.15 | 0.863, 0.165, 0.953 |
| Metatarsus | 1000 | 0.004 | 4.446 | 0.221, 0.393, 0.114 | 0.082, 0.089, 0.066 |
| Digit II | 1000 | 0.001 | 0.656 | 0.444, 0.159, 0.051 | 0.002, 0.003, 0.004 |
| Digit III | 1000 | 0.001 | 0.807 | 0.51, 0.157, 0.106 | 0.003, 0.006, 0.009 |
| Digit IV | 1000 | 0.001 | 0.799 | 0.441, 0.157, 0.142 | 0.002, 0.004, 0.005 |
| Pes | 1000 | 0.003 | 2.262 | 0.466, 0.158, 0.103 | - |
| Hind limb | 1000 | 0.076 | 76.045 | 0.088, 1.196, 0.163 | - |
|  |  |  |  |  |  |
| HAT | 909.37 | 0.303 | 275.539 | 0.318, 1.677, 0 | 16.947, 122.407, 134.632 |
| Whole Body | 939.84 | 0.455 | 427.629 | 0.236, 1.506, 0 | - |

Table S40. Results for the Plus 15% best estimate model of *Struthiomimus sedens* 1266 with reduced body air sacs.

| Segment | Net Density (kg m-3) | Volume (m3) | Mass (kg) | CM (x,y,z) (m) | Ixx Iyy Izz (kg m2) |
| --- | --- | --- | --- | --- | --- |
| Head | 974.01 | 0.0016 | 1.649 | 1.894, 2.302, 0 | 0.649, 4.043, 4.690 |
| Air sacs | 0 | 0.00042 | 0 | - | - |
|  |  |  |  |  |  |
| Neck | 930.63 | 0.027 | 25.675 | 1.329, 1.901, 0 | 2.561, 26.536, 29.090 |
| Pharyngeal cavity | 0 | 0.001 | 0 | - | - |
|  |  |  |  |  |  |
| Thorax | 854.14 | 0.183 | 160.742 | 0.650, 1.654, 0 | 8.599, 25.756, 30.706 |
| Lungs | 0 | 0.022 | 0 | - | - |
|  |  |  |  |  |  |
| Sacrum | 1000 | 0.087 | 87.097 | -0.019, 1.627, 0 | 3.485, 15.033, 17.908 |
|  |  |  |  |  |  |
| Tail | 1000 | 0.049 | 49.185 | -0.858, 1.818, 0 | 5.055, 79.290, 84.052 |
|  |  |  |  |  |  |
| Arm | 1000 | 0.008 | 8.042 | 0.887, 1.384, 0.227 | 1.352, 2.973, 3.445 |
| Digit I | 1000 | 0.0001 | 0.157 | 0.887, 1.384, 0.227 | 0.100, 0.010, 0.164 |
| Digit II | 1000 | 0.0001 | 0.141 | 1.053, 0.953, 0.335 | 0.104, 0.08, 0.154 |
| Digit III | 1000 | 0.0001 | 0.132 | 0.981, 0.904, 0.326 | 0.049, 0.039, 0.072 |
| Fore limb | 1000 | 0.008 | 8.472 | 0.894, 1.36, 0.232 | 1.606, 3.193, 3.835 |
|  |  |  |  |  |  |
| Thigh | 1000 | 0.058 | 57.575 | 0.064, 1.607, 0.191 | 2.233, 1.666, 3.537 |
| Shank | 1000 | 0.028 | 27.984 | 0.194, 0.973, 0.15 | 1.186, 0.297, 1.344 |
| Metatarsus | 1000 | 0.006 | 6.015 | 0.216, 0.397, 0.11 | 0.067, 0.046, 0.086 |
| Digit II | 1000 | 0.001 | 0.656 | 0.444, 0.159, 0.051 | 0.001, 0.003, 0.004 |
| Digit III | 1000 | 0.001 | 0.807 | 0.51, 0.157, 0.106 | 0.003, 0.006, 0.009 |
| Digit IV | 1000 | 0.001 | 0.799 | 0.441, 0.157, 0.142 | 0.002, 0.004, 0.005 |
| Pes | 1000 | 0.003 | 2.262 | 0.466, 0.158, 0.103 | - |
| Hind limb | 1000 | 0.095 | 93.836 | 0.093, 1.160, 0.171 | - |
|  |  |  |  |  |  |
| HAT | 925.34 | 0.369 | 341.449 | 0.331, 1.677, 0 | 23.561, 157.045, 174.118 |
| Whole Body | 951.66 | 0.556 | 529.121 | 0.246, 1.494, 0 |  |

Table S41. Results for the Minus 7.5% best estimate model of *Struthiomimus sedens* 1266 with enlarged body air sacs.

| Segment | Net Density (kg m-3) | Volume (m3) | Mass (kg) | CM (x,y,z) (m) | Ixx Iyy Izz (kg m2) |
| --- | --- | --- | --- | --- | --- |
| Head | 974.01 | 0.0016 | 1.649 | 1.894, 2.302, 0 | 0.653, 4.179, 4.829 |
| Air sacs | 0 | 0.0004 | 0 | - | - |
|  |  |  |  |  |  |
| Neck | 903.53 | 0.017 | 14.526 | 1.344, 1.927, 0 | 1.931, 16.310, 17.411 |
| Pharyngeal cavity | 0 | 0.003 | 0 | - | - |
|  |  |  |  |  |  |
| Thorax | 794.76 | 0.133 | 101.201 | 0.650, 1.663, 0 | 4.848, 17.242, 20.412 |
| Lungs | 0 | 0.031 | 0 | - | - |
|  |  |  |  |  |  |
| Sacrum | 1000 | 0.08 | 80.052 | -0.018, 1.641, 0 | 2.713, 12.008, 14.197 |
|  |  |  |  |  |  |
| Tail | 1000 | 0.032 | 31.601 | -0.853, 1.832, 0 | 3.253, 48.690, 51.821 |
|  |  |  |  |  |  |
| Arm | 1000 | 0.008 | 8.042 | 0.887, 1.384, 0.227 | 1.344, 3.210, 3.675 |
| Digit I | 1000 | 0.0001 | 0.157 | 0.887, 1.384, 0.227 | 0.010, 0.106, 0.170 |
| Digit II | 1000 | 0.0001 | 0.141 | 1.053, 0.953, 0.335 | 0.104, 0.088, 0.159 |
| Digit III | 1000 | 0.0001 | 0.132 | 0.981, 0.904, 0.326 | 0.049, 0.041, 0.075 |
| Fore limb | 1000 | 0.0083 | 8.472 | 0.894,1.36,0.232 | 1.597, 3.445, 4.078 |
|  |  |  |  |  |  |
| Thigh | 1000 | 0.042 | 41.81 | 0.061, 1.617, 0.173 | 1.633, 1.008, 2.436 |
| Shank | 1000 | 0.017 | 17.02 | 0.189, 0.983, 0.152 | 0.726, 0.127, 0.798 |
| Metatarsus | 1000 | 0.004 | 4.009 | 0.222, 0.4, 0.116 | 0.047, 0.031, 0.066 |
| Digit II | 1000 | 0.001 | 0.656 | 0.444, 0.159, 0.051 | 0.001, 0.003, 0.004 |
| Digit III | 1000 | 0.001 | 0.807 | 0.51, 0.157, 0.106 | 0.003, 0.006, 0.009 |
| Digit IV | 1000 | 0.001 | 0.799 | 0.441, 0.157, 0.142 | 0.002, 0.004, 0.005 |
| Pes | 1000 | 0.003 | 2.262 | 0.466, 0.158, 0.103 | - |
| Hind limb | 1000 | 0.066 | 65.101 | 0.094, 1.197, 0.162 | - |
|  |  |  |  |  |  |
| HAT | 866.65 | 0.284 | 246.13 | 0.305, 1.674, 0 | 16.591, 105.319, 116.826 |
| Whole Body | 906.8 | 0.415 | 376.332 | 0.232, 1.510, 0 | - |

Table S42. Results for the best estimate model of *Edmontosaurus annectens* 126950 with an enlarged lung.

| Segment | Net Density (kg m-3) | Volume (m3) | Mass (kg) | CM (x,y,z) (m) | Ixx Iyy Izz (kg m2) |
| --- | --- | --- | --- | --- | --- |
| Head | 962.4 | 0.028 | 27.17 | 1.082, 1.623, 0 | 5.176, 45.539, 50.496 |
| Air sacs | 0 | 0.006 | 0 | - | - |
|  |  |  |  |  |  |
| Neck | 1000 | 0.025 | 25.36 | 0.837, 1.202, 0 | 0.936, 27.915, 28.570 |
|  |  |  |  |  |  |
| Thorax | 764.97 | 0.264 | 191.005 | 0.244, 1.079, 0 | 18.140, 50.197, 60.652 |
| Lungs | 0 | 0.073 | 0 | - | - |
|  |  |  |  |  |  |
| Sacrum | 1000 | 0.177 | 176.939 | -0.589, 1.275, 0 | 9.229, 41.433, 48.499 |
|  |  |  |  |  |  |
| Tail | 1000 | 0.071 | 71.281 | -1.556, 1.36, 0 | 3.256, 139.304, 142.245 |
|  |  |  |  |  |  |
| Fore limb | 1000 | 0.011 | 11.031 | 0.618, 0.578, 0.129 | 4.79973, 7.80381, 12.192 |
|  |  |  |  |  |  |
| Thigh | 1000 | 0.112 | 112..431 | -0.588, 1.184, 0.197 | 3.984, 6.623, 9.137 |
| Shank | 1000 | 0.23 | 22.757 | -0.346, 0.575, 0.237 | 0.655, 0.261, 0.788 |
| Metatarsus | 1000 | 0.005 | 4.582 | -0.396, 0.2, 0.203 | 0.023, 0.023, 0.020 |
| Digit II | 1000 | 0.001 | 1.158 | -0.299, 0.084, 0.051 | 0.003, 0.004, 0.003 |
| Digit III | 1000 | 0.001 | 1.457 | -0.296, 0.095, 0.184 | 0.003, 0.007, 0.007 |
| Digit IV | 1000 | 0.001 | 0.925 | -0.296, 0.061, 0.281 | 0.002, 0.003, 0.003 |
| Pes | 1000 | 0.004 | 4.473 | -0.27, 0.077, 0.171 | - |
| Hind limb | 1000 | 0.144 | 144.243 | -0.507, 0.976, 0.203 | - |
|  |  |  |  |  |  |
| HAT | 875.33 | 0.587 | 513.817 | -0.203, 1.199, 0 | 46.337, 319.996, 354.845 |
| Whole Body | 916.92 | 0.875 | 802.303 | -0.312191 1.11852 0 | - |

Table S43. Results for the best estimate model of *Edmontosaurus annectens* 126950 with reduced body air sacs.

| Segment | Net Density (kg m-3) | Volume (m3) | Mass (kg) | CM (x,y,z) (m) | Ixx Iyy Izz (kg m2) |
| --- | --- | --- | --- | --- | --- |
| Head | 962.4 | 0.028 | 27.17 | 1.082, 1.623, 0 | 5.357, 44.457, 49.594 |
| Air sacs | 0 | 0.006 | 0 | - | - |
|  |  |  |  |  |  |
| Neck | 1000 | 0.025 | 25.36 | 0.837, 1.202, 0 | 0.939, 27.099, 27.756 |
|  |  |  |  |  |  |
| Thorax | 764.97 | 0.264 | 211.646 | 0.239, 1.071, 0 | 18.402, 51.691, 62.122 |
| Lungs | 0 | 0.052 | 0 | - | - |
|  |  |  |  |  |  |
| Sacrum | 1000 | 0.177 | 176.939 | -0.589, 1.275, 0 | 9.449, 43.606, 50.892 |
|  |  |  |  |  |  |
| Tail | 1000 | 0.071 | 71.281 | -1.556, 1.36, 0 | 3.439, 142.329, 145.453 |
|  |  |  |  |  |  |
| Fore limb | 1000 | 0.011 | 11.031 | 0.618, 0.578, 0.129 | 4.694, 7.524, 11.806 |
|  |  |  |  |  |  |
| Thigh | 1000 | 0.112 | 112..431 | -0.588, 1.184, 0.197 | 3.984, 6.623, 9.137 |
| Shank | 1000 | 0.23 | 22.757 | -0.346, 0.575, 0.237 | 0.655, 0.261, 0.788 |
| Metatarsus | 1000 | 0.005 | 4.582 | -0.396, 0.2, 0.203 | 0.023, 0.023, 0.020 |
| Digit II | 1000 | 0.001 | 1.158 | -0.299, 0.084, 0.051 | 0.003, 0.004, 0.003 |
| Digit III | 1000 | 0.001 | 1.457 | -0.296, 0.095, 0.184 | 0.003, 0.007, 0.007 |
| Digit IV | 1000 | 0.001 | 0.925 | -0.296, 0.061, 0.281 | 0.002, 0.003, 0.003 |
| Pes | 1000 | 0.004 | 4.473 | -0.27,0.077,0.171 | - |
| Hind limb | 1000 | 0.144 | 144.243 | -0.507, 0.976, 0.203 | - |
|  |  |  |  |  |  |
| HAT | 910.49 | 0.587 | 534.458 | -0.187, 1.191, 0 | 46.974, 324.23, 359.429 |
| Whole Body | 940.51 | 0.875 | 822.944 | -0.299, 1.115, 0 | - |

Table S44. Results for the Plus 15% best estimate model of *Edmontosaurus annectens* 12695 with reduced body air sacs.

| Segment | Net Density (kg m-3) | Volume (m3) | Mass (kg) | CM (x,y,z) (m) | Ixx Iyy Izz (kg m2) |
| --- | --- | --- | --- | --- | --- |
| Head | 962.4 | 0.028 | 27.17 | 1.082, 1.623, 0 | 6.264, 32.264, 38.309 |
| Air sacs | 0 | 0.006 | 0 | - | - |
|  |  |  |  |  |  |
| Neck | 1000 | 0.032 | 32.178 | 0.837, 1.202, 0 | 1.173, 22.976, 23.688 |
|  |  |  |  |  |  |
| Thorax | 814.63 | 0.334 | 281.779 | 0.228, 1.026, 0 | 29.649, 32.781, 49.351 |
| Lungs | 0 | 0.052 | 0 | - | - |
|  |  |  |  |  |  |
| Sacrum | 1000 | 0.196 | 196.416 | -0.596, 1.242, 0 | 12.572, 87.967, 98.176 |
|  |  |  |  |  |  |
| Tail | 1000 | 0.095 | 94.58 | -1.556, 1.346, 0 | 5.998, 14.197, 19.628 |
|  |  |  |  |  |  |
| Fore limb | 1000 | 0.011 | 11.031 | 0.618, 0.578, 0.129 | 4.208, 4.530, 8.327 |
|  |  |  |  |  |  |
| Thigh | 1000 | 0.129 | 129.235 | -0.597, 1.181, 0.213 | 4.726, 7.919, 10.553 |
| Shank | 1000 | 0.03 | 30.259 | -0.341, 0.576, 0.246 | 0.902, 0.411, 1.090 |
| Metatarsus | 1000 | 0.006 | 5.732 | -0.389, 0.2, 0.21 | 0.033, 0.035, 0.026 |
| Digit II | 1000 | 0.001 | 1.158 | -0.299, 0.084, 0.051 | 0.003, 0.004, 0.003 |
| Digit III | 1000 | 0.001 | 1.457 | -0.296, 0.095, 0.184 | 0.003, 0.007, 0.007 |
| Digit IV | 1000 | 0.001 | 0.925 | -0.296, 0.061, 0.281 | 0.002, 0.003, 0.003 |
| Pes | 1000 | 0.004 | 4.473 | -0.27, 0.077, 0.171 | - |
| Hind limb | 1000 | 0.169 | 169.699 | -0.505, 0.958, 0.218 | - |
|  |  |  |  |  |  |
| HAT | 926.61 | 0.706 | 654.185 | 0.004, 1.154, 0 | 64.071, 199.247, 245.806 |
| Whole Body | 949.89 | 1.046 | 993.583 | -0.170, 1.087, 0 | - |

Table S45. Results for the Minus 7.5% best estimate model of *Edmontosaurus annectens* 126950 with an enlarged lung.

| Segment | Net Density (kg m-3) | Volume (m3) | Mass (kg) | CM (x,y,z) (m) | Ixx Iyy Izz (kg m2) |
| --- | --- | --- | --- | --- | --- |
| Head | 962.4 | 0.028 | 27.17 | 1.082, 1.624, 0 | 4.750, 45.601, 50.131 |
| Air sacs | 0 | 0.006 | 0 | - | - |
|  |  |  |  |  |  |
| Neck | 1000 | 0.021 | 25.36 | 0.837, 1.202, 0 | 0.759, 23.791, 24.339 |
|  |  |  |  |  |  |
| Thorax | 732.81 | 0.231 | 158.333 | 0.254, 1.107, 0 | 13.590, 42.829, 50.716 |
| Lungs | 0 | 0.073 | 0 | - | - |
|  |  |  |  |  |  |
| Sacrum | 1000 | 0.173 | 172.552 | -0.589, 1.285, 0 | 8.187, 40.238, 46.289 |
|  |  |  |  |  |  |
| Tail | 1000 | 0.06 | 60.26 | -1.556, 1.366, 0 | 2.328, 117.57, 119.666 |
|  |  |  |  |  |  |
| Fore limb | 1000 | 0.011 | 11.031 | 0.618, 0.578, 0.129 | 5.06259, 7.820, 12.471 |
|  |  |  |  |  |  |
| Thigh | 1000 | 0.107 | 106.938 | -0.6, 1.191, 0.19 | 3.695, 6.134, 8.538 |
| Shank | 1000 | 0.019 | 19.499 | -0.35, 0.574, 0.233 | 0.552, 0.206, 0.664 |
| Metatarsus | 1000 | 0.004 | 4.128 | -0.397, 0.197, 0.2 | 0.020, 0.019, 0.018 |
| Digit II | 1000 | 0.001 | 1.158 | -0.299, 0.084, 0.051 | 0.028, 0.029, 0.024 |
| Digit III | 1000 | 0.001 | 1.457 | -0.296, 0.095, 0.184 | 0.003, 0.007, 0.007 |
| Digit IV | 1000 | 0.001 | 0.925 | -0.296, 0.061, 0.281 | 0.002, 0.003, 0.003 |
| Pes | 1000 | 0.004 | 4.473 | -0.27, 0.077, 0.171 | - |
| Hind limb | 1000 | 0.135 | 135.038 | -0.521, 0.992, 0.196 | - |
|  |  |  |  |  |  |
| HAT | 863.01 | 0.535 | 461.713 | -0.204, 1.218, 0 | 39.739, 285.669, 316.084 |
| Whole Body | 909.05 | 0.805 | 731.789 | -0.321, 1.134, 0 | - |

Table S46. Results for the best estimate model of *Tyrannosaurus rex* BHI 3033 with an abdominal air sac..

| Segment | Net Density (kg m-3) | Volume (m3) | Mass (kg) | CM (x,y,z) (m) | Ixx Iyy Izz (kg m2) |
| --- | --- | --- | --- | --- | --- |
| Head | 990.6 | 0.685 | 678.561 | 2.553, 3.346, 0 | 171.259, 8133.03, 8212.11 |
| Air sacs | 0 | 0.01 | 0 | - | - |
|  |  |  |  |  |  |
| Neck | 905.3 | 0.369 | 334.056 | 1.690, 3.376, 0 | 56.872, 1287, 1320.64 |
| Pharyngeal cavity | 0 | 0.035 | 0 | - | - |
|  |  |  |  |  |  |
| Thorax | 746.46 | 3.01 | 2108.44 | 0.384, 3.003, 0 | 776.246, 1707.48, 2081.24 |
| Lungs | 0 | 0.764 | 0 | - | - |
| Abdominal air sacs | 0 | 0.138 | 0 | - | - |
|  |  |  |  |  |  |
| Sacrum | 1000 | 1.062 | 1062.439 | -1.319, 3.004, 0 | 288.692, 1450.87, 1707.28 |
|  |  |  |  |  |  |
| Tail | 1000 | 1.106 | 1106.037 | -3.530, 3.092, 0 | 297.803, 13305.1, 13573.4 |
|  |  |  |  |  |  |
| Arm | 1000 | 0.011 | 10.931 | 1.138, 2.398, 0.483 | 8.868, 23.817, 27.406 |
| Digit I | 1000 | 0.001 | 0.644 | 1.315, 2.115, 0.62 | 0.933, 1.833, 2.269 |
| Digit II | 1000 | 0.001 | 0.548 | 1.33, 2.197, 0.591 | 0.684957, 1.566, 1.867 |
| Fore limb | 1000 | 0.013 | 12.123 | 1.156, 2.374, 0.495 | 10.486, 27.215, 31.543 |
|  |  |  |  |  |  |
| Thigh | 1000 | 0.744 | 743.937 | -1.323, 2.853, 0.408 | 183.542, 78.453, 226.284 |
| Shank | 1000 | 0.215 | 214.664 | -1.887, 1.687, 0.357 | 13.8558, 21.791, 30.382 |
| Metatarsus | 1000 | 0.074 | 73.812 | -2.431, 0.914, 0.298 | 3.380, 1.501, 3.258 |
| Digit II | 1000 | 0.023 | 22.332 | -1.897, 0.11, 0.29 | 0.257, 0.259, 0.499 |
| Digit III | 1000 | 0.027 | 26.564 | -1.811, 0.134, 0.457 | 0.308, 0.540, 0.781 |
| Digit IV | 1000 | 0.021 | 20.966 | -1.998, 0.09, 0.571 | 0.241, 0.242, 0.452 |
| Pes | 1000 | 0.07 | 69.863 | -2.112, 0.681, 0.72 | - |
| Hind limb | 1000 | 1.102 | 1102.276 | -1.373, 2.194, 0.410 | - |
|  |  |  |  |  |  |
| HAT | 855.98 | 6.257 | 5570.72 | -0.253, 3.146, 0 | 1585.788, 25933.72, 26928.41 |
| Whole Body | 918.8 | 8.462 | 7775.27 | -0.570, 2.876, 0 | - |

Table S47. Results for the best estimate model of *Tyrannosaurus rex* MOR 555 with an abdominal air sac..

| Segment | Net Density (kg m-3) | Volume (m3) | Mass (kg) | CM (x,y,z) (m) | Ixx Iyy Izz (kg m2) |
| --- | --- | --- | --- | --- | --- |
| Head | 984.26 | 0.661 | 650.596 | 3.884, 3.063, 0 | 226.136, 5818.47, 5984.67 |
| Air sacs | 0 | 0.011 | 0 | - | - |
|  |  |  |  |  |  |
| Neck | 938.15 | 0.471 | 441.871 | 2.783, 3.101, 0 | 140.729, 1666.04, 1797.07 |
| Pharyngeal cavity | 0 | 0.029 | 0 | - | - |
|  |  |  |  |  |  |
| Thorax | 739.52 | 1.721 | 1171.51 | 1.387, 2.669, 0 | 290.265, 531.646, 805.356 |
| Lungs | 0 | 0.449 | 0 | - | - |
| Abdominal air sacs | 0 | 0.101 | 0 | - | - |
|  |  |  |  |  |  |
| Sacrum | 1000 | 0.659 | 659.477 | 0.222, 2.272, 0 | 290.432, 375.532, 655.449 |
|  |  |  |  |  |  |
| Tail | 1000 | 1.079 | 1078.774 | -1.828, 2.363, 0 | 183.677, 9391.19, 9553.01 |
|  |  |  |  |  |  |
| Arm | 1000 | 0.009 | 8.845 | 1.89, 2.097, 0.277 | 3.394, 9.600, 11.605 |
| Digit I | 1000 | 0.0003 | 0.355 | 2.16, 1.922, 0.202 | 0.193, 0.589, 0.752 |
| Digit II | 1000 | 0.001 | 0.822 | 2.172, 1.846, 0 | 0.540, 1.890, 1.862 |
| Fore limb | 1000 | 0.010 | 10.022 | 1.923, 0.294, 0.268 | 4.127, 11.579, 14.219 |
|  |  |  |  |  |  |
| Thigh | 1000 | 0.689 | 688.552 | -0.114, 2.289, 0.363 | 149.268, 76.0171, 194.264 |
| Shank | 1000 | 0.212 | 212.459 | -0.584, 1.114, 0.426 | 13.684, 19.713, 27.550 |
| Metatarsus | 1000 | 0.044 | 44.408 | -0.859, 0.392, 0.417 | 1.204, 0.906, 1.343 |
| Digit II | 1000 | 0.007 | 7.128 | -0.477, 0.077, 0.25 | 0.038, 0.166, 0.155 |
| Digit III | 1000 | 0.01 | 9.765 | -0.432, 0.09, 0.451 | 0.036, 0.336, 0.331 |
| Digit IV | 1000 | 0.009 | 8.814 | -0.489, 0.091, 0.614 | 0.054, 0.227, 0.203 |
| Pes | 1000 | 0.026 | 25.707 | -0.464, 0.086, 0.449 | - |
| Hind limb | 1000 | 0.971 | 971.126 | -0.221, 1.760, 0.382 | - |
|  |  |  |  |  |  |
| HAT | 872.08 | 4.612 | 4022.03 | 0.889, 2.630, 0 | 1138.764,  17804.1,  18821.4 |
| Whole Body | 910.99 | 6.554 | 5970.62 | 0.527, 2.346, 0 | - |

Table S48. Results for the best estimate model of *Acrocanthosaurus atokensis* NCSM 14345 with an abdominal air sac..

| Segment | Net Density (kg m-3) | Volume (m3) | Mass (kg) | CM (x,y,z) (m) | Ixx Iyy Izz (kg m2) |
| --- | --- | --- | --- | --- | --- |
| Head | 981.63 | 0.405 | 397.566 | 3.437, 2.138, 0 | 46.228, 4523.74, 4555.97 |
| Air sacs | 0 | 0.007 | 0 | - | - |
|  |  |  |  |  |  |
| Neck | 911.07 | 0.336 | 306.118 | 2.325, 2.181, 0 | 42.832, 1575.6, 1607.21 |
| Pharyngeal cavity | 0 | 0.03 | 0 | - | - |
|  |  |  |  |  |  |
| Thorax | 760.07 | 2.42 | 1759.34 | 1.049, 2.19, 0 | 450.958, 2320.28, 2500.98 |
| Lungs | 0 | 0.58 | 0 | - | - |
| Abdominal air sacs | 0 | 0.08 | 0 | - | - |
|  |  |  |  |  |  |
| Sacrum | 1000 | 0.768 | 768.158 | -0.54,  2.41, 0 | 192.522, 455.641, 633.843 |
|  |  |  |  |  |  |
| Tail | 1000 | 1.149 | 1148.734 | -2.77, 2.465, 0 | 125.855, 10863., 10958.9 |
|  |  |  |  |  |  |
| Arm | 1000 | 0.01 | 10.024 | 1.694, 1.252, -0.414 | 12.846, 27.814, 37.085 |
| Digit I | 1000 | 0.0005 | 0.491 | 1.721, 0.919, -0.585 | 1.093, 1.484, 2.241 |
| Digit II | 1000 | 0.001 | 1.207 | 1.814, 0.852, -0.531 | 2.846, 3.954, 6.118 |
| Digit IV | 1000 | 0.0006 | 0.639 | 1.815, 0.898, -0.422 | 1.354, 2.030, 3.155 |
| Forelimb | 1000 | 0.012 | 12.361 | 1.713, 1.181, 0.433 | 18.139, 35.281, 48.599 |
|  |  |  |  |  |  |
| Thigh | 1000 | 0.664 | 663.709 | -0.495, 2.067, 0.35 | 91.336, 97.478, 159.109 |
| Shank | 1000 | 0.142 | 142.12 | -0.255, 0.936, 0.308 | 11.385, 5.102, 13.239 |
| Metatarsus | 1000 | 0.033 | 32.925 | -0.277, 0.236, 0.32 | 0.670387, 0.632758, 0.762 |
| Digit II | 1000 | 0.002 | 2.257 | 0.023, 0.067, 0.185 | 0.003, 0.004, 0.004 |
| Digit III | 1000 | 0.003 | 3.647 | 0.105, 0.026, 0.341 | 0.006, 0.008, 0.008 |
| Digit IV | 1000 | 0.002 | 1.767 | 0.019, 0.071, 0.491 | 0.002, 0.002, 0.003 |
| Pes | 1000 | 0.007 | 7.671 | 0.062, 0.049, 0.33 | - |
| Hind limb | 1000 | 0.847 | 846.524 | -0.441, 1.788, 0.342 | - |
|  |  |  |  |  |  |
| HAT | 842.67 | 5.227 | 4404.64 | 0.084, 2.291, 0 | 889.381, 19801.31, 20342.59 |
| Whole Body | 882.09 | 6.912 | 6097 | -0.062, 2.151, 0 | - |

Table S49. Results for the best estimate model of *Struthiomimus sedens* 1266 with an abdominal air sac.

| Segment | Net Density (kg m-3) | Volume (m3) | Mass (kg) | CM (x,y,z) (m) | Ixx Iyy Izz (kg m2) |
| --- | --- | --- | --- | --- | --- |
| Head | 974.01 | 0.002 | 1.649 | 1.894, 2.302, 0 | 0.626, 4.217, 4.840 |
| Air sacs | 0 | 0.0042 | 0 | - | - |
|  |  |  |  |  |  |
| Neck | 901.81 | 0.02 | 18.029 | 1.340, 1.919, 0 | 1.781, 20.235, 22.131 |
| Pharyngeal cavity | 0 | 0.0021 | 0 | - | - |
|  |  |  |  |  |  |
| Thorax | 809.72 | 0.142 | 102.402 | 0.661, 1.685, 0 | 4.282, 19.378, 21.618 |
| Lungs | 0 | 0.027 | 0 | - | - |
| Abdominal air sacs | 0 | 0.013 | 0 | - | - |
|  |  |  |  |  |  |
| Sacrum | 1000 | 0.082 | 81.723 | -0.019, 1.639, 0 | 2.919, 12.044, 14.415 |
|  |  |  |  |  |  |
| Tail | 1000 | 0.037 | 37.067 | -0.854, 1.827, 0 | 3.629, 56.482, 59.943 |
|  |  |  |  |  |  |
| Arm | 1000 | 0.008 | 8.042 | 0.887, 1.384, 0.227 | 1.408, 3.279, 3.806 |
| Digit I | 1000 | 0.0001 | 0.157 | 0.887, 1.384, 0.227 | 0.103, 0.107, 0.175 |
| Digit II | 1000 | 0.0001 | 0.141 | 1.053, 0.953, 0.335 | 0.106, 0.0890, 0.163 |
| Digit III | 1000 | 0.0001 | 0.132 | 0.981, 0.904, 0.326 | 0.051, 0.042, 0.077 |
| Fore limb | 1000 | 0.008 | 8.472 | 0.894, 1.36, 0.232 | 1.668, 3.517, 4.221 |
|  |  |  |  |  |  |
| Thigh | 1000 | 0.049 | 49.349 | 0.057, 1.604, 0.175 | 1.975, 1.273, 2.979 |
| Shank | 1000 | 0.02 | 19.988 | 0.188, 0.966, 0.15 | 0.864, 0.165, 0.953 |
| Metatarsus | 1000 | 0.004 | 4.446 | 0.221, 0.393, 0.114 | 0.082, 0.089, 0.066 |
| Digit II | 1000 | 0.001 | 0.656 | 0.444, 0.159, 0.051 | 0.001, 0.003, 0.004 |
| Digit III | 1000 | 0.001 | 0.807 | 0.51, 0.157, 0.106 | 0.003, 0.006, 0.009 |
| Digit IV | 1000 | 0.001 | 0.799 | 0.441, 0.157, 0.142 | 0.002, 0.004, 0.005 |
| Pes | 1000 | 0.003 | 2.262 | 0.466, 0.158, 0.103 | - |
| Hind limb | 1000 | 0.076 | 76.045 |  | - |
|  |  |  |  |  |  |
| HAT | 862.78 | 0.23 | 257.971 | 0.297, 1.688, 0 | 16.572, 119.390, 131.389 |
| Whole Body | 907.21 | 0.452 | 410.061 | 0.220, 1.505, 0 | - |
